# IRF7 links HK1-dependent histone lactylation to fibroblast activation and cardiac fibrosis

**DOI:** 10.1038/s44321-026-00444-2

**Published:** 2026-05-21

**Authors:** Ming Kong, Chenghao Zhu, Yujia Xue, Wenxuan Hong, Guoqing Zhang, Dingsheng Jiang, Yong Xu, Junli Guo

**Affiliations:** 1 State Key Laboratory of Natural Medicines, Department of Pharmacology, China Pharmaceutical University, Nanjing, China; 2 Department of Cardiology, Affiliated Hospital of Jiangnan University, Wuxi, China; 3 Department of Geriatric Nephrology, Jiangsu Province People’s Hospital, the First Affiliated Hospital of Nanjing Medical University, Nanjing, China; 4 Division of Cardiovascular Surgery, Tongji Hospital, Tongji Medical College, Huazhong University of Science and Technology, Wuhan, China; 5 Institute of Biomedical Research, College of Agriculture and Biology, Liaocheng University, Liaocheng, China; 6 Hainan Provincial Key Laboratory for Tropical Cardiovascular Diseases Research and Key Laboratory of Emergency and Trauma of Ministry of Education, Institute of Cardiovascular Research, Department of Cardiology, the First Affiliated Hospital, Hainan Medical University, Haikou, China

**Keywords:** Cardiovascular System

## Abstract

In response to various stimuli, quiescent resident cardiac fibroblasts undergo metabolic, morphological, and functional alterations, transitioning into myofibroblasts that mediate cardiac fibrosis. In the present study, we investigated the role of interferon regulatory factor 7 (IRF7) in fibroblast activation and cardiac fibrosis. Knockdown of IRF7 in quiescent cardiac fibroblasts potentiated myofibroblast transition, whereas overexpression of IRF7 suppressed it. Furthermore, targeted deletion of IRF7 in fibroblasts or myofibroblasts exacerbated cardiac fibrosis and impaired heart function in animal models of heart failure. Integrated transcriptomic analysis revealed hexokinase 1 (HK1) as an IRF7 downstream target. Consistently, genetic deletion or pharmacological inhibition of HK1 protected mice from adverse cardiac remodeling. Mechanistically, HK1 contributed to the cellular lactate pool, promoting histone H3K9 lactylation and enabling the transcription of pro-fibrogenic molecules. Finally, the relevance of the IRF7-HK1 axis was verified in human heart specimens. In conclusion, our data unveil an IRF7-HK1 axis that contributes to the metaboloepigenetic reprogramming of fibroblast activation and cardiac fibrosis. Targeting this axis may yield new therapeutic solutions for heart failure intervention.

## Synopsis

The activation of quiescent fibroblasts contributes to adverse ventricular remodeling following myocardial injury, which can lead to heart failure. The transcription factor IRF7 links HK1-dependent histone lactylation to fibroblast activation and cardiac fibrosis.

- IRF7 deletion promotes fibroblast activation and aggravates cardiac fibrosis.
- IRF7 over-expression dampens fibroblast activation and attenuates cardiac fibrosis
- IRF7 binds to the HK1 promoter and represses HK1 transcription.
- Genetic deletion or pharmaceutical inhibition of HK1 mitigates cardiac fibrosis.
- HK1-dependent H3K9 lactylation facilitates recruitment of SMAD3, c-JUN, and NFATC2 to orchestrate pro-fibrogenic transcription.

## Abstract (web summary)

The activation of quiescent fibroblasts contributes to adverse ventricular remodeling following myocardial injury, which can lead to heart failure. The transcription factor IRF7 links HK1-dependent histone lactylation to fibroblast activation and cardiac fibrosis.

## Graphical abstract

**Figure:**
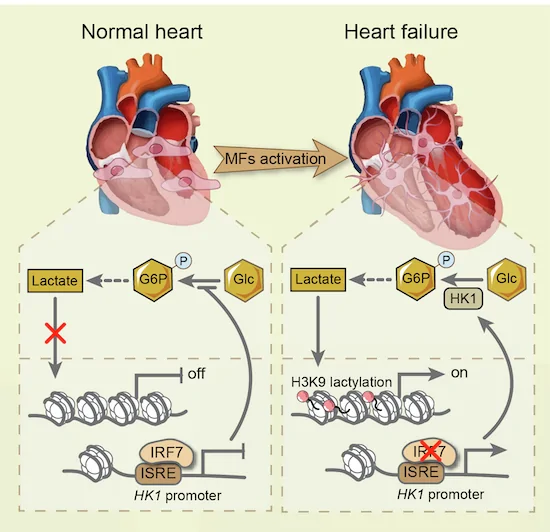

**Figure:**
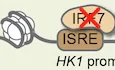

The paper explained **Problem**

Diffuse myocardial fibrosis impairs heart function, leading to heart failure (HF). The trans-differentiation of quiescent cardiac fibroblasts into myofibroblasts is a key process in cardiac fibrosis.

**Results**

We present a new mechanism through which the transcription factor IRF7 connects HK1-mediated histone lactylation to fibroblasts–myofibroblasts transition and cardiac fibrosis. Genetic and/or pharmacological manipulation of the IRF7–HK1 axis in mice mitigates cardiac fibrosis and prevents heart failure. Notably, a correlation between IRF7, HK1, and myofibroblast activation is identified in heart tissue samples taken from patients with heart failure (HF).

**Impact**

These data offer new insights and translational potential for HF intervention.

## Introduction

Heart failure (HF) is a complex syndrome with diverse underlying causes and mechanisms. It affects more than 50 million people worldwide and accounts for over half a million premature deaths annually in the United States alone (Martin et al, 2024). Commonly identified risk factors for HF include senility, physical inactivity, unhealthy diet, substance abuse, hypertension, dyslipidemia, obesity, and diabetes. Driven by the global epidemic of obesity and diabetes and an increasingly aging population, the prevalence of heart failure is expected to rise over the next decade, with total medical costs—both direct and indirect—projected to reach trillions. (Khan et al, 2024). Regardless of etiology, diffuse myocardial fibrosis is both a prominent pathological feature in and a key contributing factor to HF (Lopez et al, 2021). Characterized by increased synthesis and deposition of extracellular matrix (ECM) components that mostly consist of fibrillar collagens (e.g., collagen type I and collagen type III), cardiac fibrosis is part of the wound healing process following myocardial injuries. However, aberrant cardiac fibrosis in response to persistent and chronic injury increases myocardial stiffness and eventually causes the loss of diastolic/systolic functions, leading to heart failure.

Myofibroblasts have long been regarded as the principal effector cells driving cardiac fibrosis. Possessing both the ability to produce ECM proteins and to handle muscle-like contraction, myofibroblasts are absent from the heart under physiological conditions, quickly emerge in response to myocardial injuries, and disappear once fibrosis is resolved (van den Borne et al, 2010). The transient nature of myofibroblasts has driven efforts to identify their cellular origins during cardiac fibrosis, leading to multiple hypotheses that endocardial and epicardial cells, endothelial cells, and myeloid lineages may contribute to the myofibroblast pool (Tallquist and Molkentin, 2017). The Molkentin group has demonstrated, by using genetic lineage tracing techniques, that cardiac resident fibroblasts are the predominant source of myofibroblasts. Whereas quiescent cardiac fibroblasts are faithfully labeled by the transcription factor TCF21, activated fibroblasts (myofibroblasts) can be identified by the matricellular protein Periostin (encoded by *Postn*) during cardiac fibrosis (Kanisicak et al, 2016). This notion is supported by the observation that ablation of Postn^+^ myofibroblasts significantly attenuates cardiac fibrosis and rescues heart function in animal models of cardiomyopathies and heart failure (Kaur et al, 2016).

When quiescent cardiac fibroblasts trans-differentiate into ECM-producing myofibroblasts (i.e., fibroblast activation) in a process now known as fibroblast–myofibroblast transition, they undergo profound morphological, functional, and transcriptomic alterations. Indeed, recent advances in single-cell transcriptomic techniques have documented in great detail various transcription events, orchestrated by sequence-specific transcription factors, taking place during FMyT (Patrick et al, 2024). Interferon regulatory factors (IRFs) are a group of transcription factors that all share an evolutionarily conserved N-terminal DNA binding domain that recognizes and binds to the interferon stimulated response element (ISRE, GAAANNGAAAC)(Weisz et al, 1992). Mounting evidence has implicated members of the IRF family as pivotal regulators of cardiovascular diseases (Sun and Wang, 2014; Zhang et al, 2015). However, there is no direct evidence that links fibroblast/myofibroblast autonomous IRF to cardiac fibrosis. A recent study showed that IRF7 is upregulated in dermal fibroblasts from patients with systemic sclerosis (SSc) and that global IRF7 knockout mice develop less dermal fibrosis than wild-type littermates when exposed to bleomycin (Wu et al, 2019). We hypothesized that IRF7 might play a similar role in cardiac fibrosis. Here, we provide evidence to show that, in contrast to the previous study (Wu et al, 2019), IRF7 is an antagonist of cardiac fibrosis. IRF7 deletion de-represses the transcription of Hexokinase 1 (HK1), which in turn promotes histone lactylation and pro-fibrogenic transcription. Our data unveil a previously unrecognized IRF7-HK1 axis that contributes to metabolic reprogramming of fibroblast activation and cardiac fibrosis.

## Results

### IRF7 manipulation influences fibroblast activation in vitro

**Figure 1:**
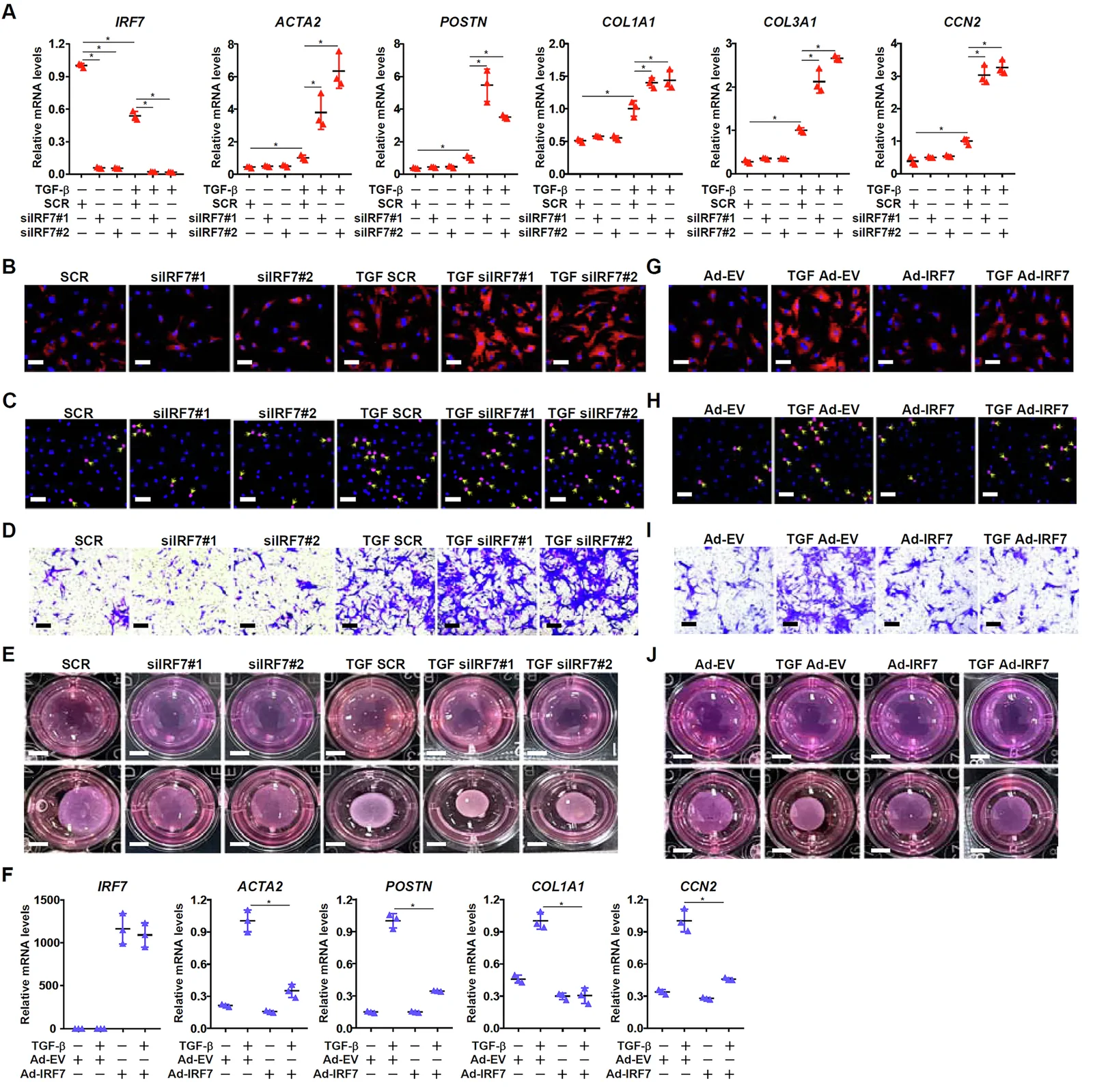
IRF7 manipulation influences fibroblast activation in vitro. (**A**–**E**) Primary human cardiac fibroblasts were transfected with indicated siRNAs followed by treatment with TGF-β (5 ng/ml) for 24 h. Myofibroblast markers were examined by qPCR (**A**) and immunofluorescence staining (**B**). EdU incorporation assay (**C**). Transwell assay (**D**). Collagen contraction assay (**E**). Scale bar = 50 μm (**B**–**D**) and scale bar = 1 cm (**E**). (**F**–**J**) Primary human cardiac fibroblasts were transduced with the indicated adenovirus, followed by treatment with TGF-β (5 ng/ml) for 24 h. Myofibroblast markers were examined by qPCR (**F**) and immunofluorescence staining (**G**). EdU incorporation assay (**H**). Transwell assay (**I**). Collagen contraction assay (**J**). Scale bar = 50 μm (**G**–**I**) and scale bar = 1 cm (**J**). *N* = 3 biological replicates. Data are expressed as mean ± SD. *P <0.05, one-way ANOVA with post hoc Scheffe´s test. Exact *P* values are reported in Appendix Table S4. Source data are available online for this figure.

In an attempt to replicate the finding that IRF7 functions as a pro-fibrogenic factor to promote skin fibrosis (Wu et al, 2019), primary human cardiac fibroblasts were transfected with siRNAs targeting IRF7 to silence endogenous IRF7 expression. IRF7 knockdown by itself did not significantly alter the expression of myofibroblast markers but amplified TGF-β-induced myofibroblast marker expression, as measured by qPCR (Fig. 1A) and α-SMA staining (Fig. 1B). In addition, IRF7 knockdown enhanced TGF-β induced cell proliferation (Fig. 1C), cell migration (Fig. 1D), and cell contraction (Fig. 1E), all indicative of an amplified myofibroblast phenotype. Similarly, Cre-mediated IRF7 deletion in primary murine cardiac fibroblasts isolated from the IRF7^f/f^ mice significantly strengthened the transition to a myofibroblast phenotype induced by TGF-β (Appendix Fig. S1). On the contrary, IRF7 overexpression, while by itself did not alter fibroblast behaviors, significantly attenuated TGF-β-induced fibroblast–myofibroblast transition in both human (Fig. 1F–J) and murine (Appendix Fig. S2) primary cardiac fibroblasts.

In order to determine whether alteration of fibroblast phenotype by IRF7 manipulation was limited to TGF-β, similar experiments were performed with a second stimulus angiotensin II (Ang II) also known to promote FMyT. Indeed, IRF7 deletion accelerated Ang II-induced fibroblast activation in vitro (Appendix Fig. S3). Taken together, these data suggest that IRF7 might play an antagonistic role in cardiac fibrosis.

### IRF7 deletion in fibroblasts/myofibroblasts aggravates cardiac fibrosis

**Figure 2:**
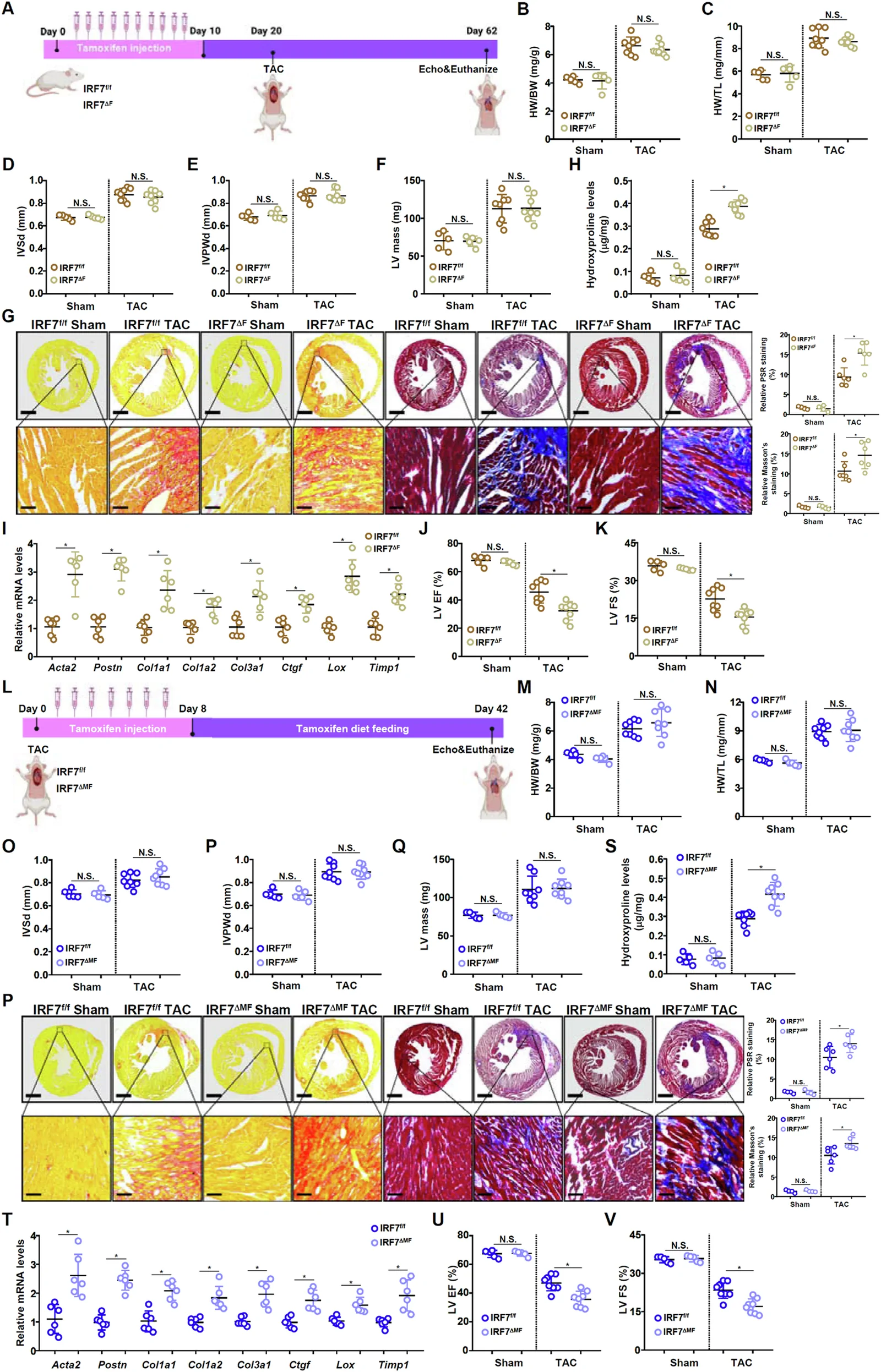
IRF7 deletion in fibroblasts/myofibroblasts aggravates cardiac fibrosis. (**A**–**K**) Fibroblast conditional IRF7 knockout (IRF7^ΔF^) and wild-type mice were subjected to the TAC procedure and euthanized 6 weeks after the surgery. Scheme of protocol (**A**). Heart weight versus body weight ratio (**B**). Heart weight versus tibia length ratio (**C**). IVSd (**D**). IVWPd (**E**). LV mass (**F**). Paraffin sections were stained with PicroSirius Red or Masson’s Trichrome (**G**). Hydroxyproline levels (**H**). Myofibroblast markers were examined by qPCR (**I**). LV EF (**J**). LV FS (**K**). (**L**–**V**) Myofibroblast conditional IRF7 knockout (IRF7^ΔMF^) and wild-type mice were subjected to the TAC procedure and euthanized 6 weeks after the surgery. Scheme of protocol (**L**). Heart weight versus body weight ratio (**M**). Heart weight versus tibia length ratio (**N**). IVSd (**O**). IVWPd (**P**). LV mass (**Q**). Paraffin sections were stained with PicroSirius Red or Masson’s Trichrome (**R**). Hydroxyproline levels (**S**). Myofibroblast markers were examined by qPCR (**T**). LV EF (**U**). LV FS (**V**). *N* = 5 mice for the sham groups and *N* = 8 mice for the TAC groups. Scale bar, 1 mm (upper panel) and 50 μm (bottom panel). Data are expressed as mean ± SD. *P <0.05, two-tailed Student’s test (**I**, **T**) or one-way ANOVA with post hoc Scheffe’s test (the rest). Exact *P* values are reported in Appendix Table S4. Source data are available online for this figure.

To replicate the observation that IRF7 might antagonize fibroblast activation in vivo, the IRF7^f/f^ mice were crossed to the *Col1a2*-Cre^ERT2^ mice to generate fibroblast-specific IRF7 deletion mice (IRF7^ΔF^); both the IRF7^f/f^ mice and the IRF7^ΔF^ mice were subjected to the TAC procedure to induce cardiac fibrosis (Fig. 2A). Compared to the control mice, the IRF7^ΔF^ mice develop equivalent levels of cardiac hypertrophy, as assessed by heart weights (Fig. 2B,C) and Doppler ultrasound measurements of chamber size (Fig. 2D–F), in response to pressure overload. On the other hand, histopathological staining (Fig. 2G), myocardial hydroxyproline quantification (Fig. 2H), and qPCR measurements of myofibroblast markers (Fig. 2I) all pointed to aggravation of cardiac fibrosis in the IRF7^ΔF^ mice compared to the control mice. Consequently, significantly worse post-surgery heart function, as evidenced by LV EF values (Fig. 2J) and LV FS values (Fig. 2K), was recorded in the IRF7^ΔF^ mice than in the control mice. Likewise, when both the IRF7^ΔF^ mice and the IRF7^f/f^ mice were subjected to permanent ligation of the left anterior descending coronary artery to induce myocardial infarction, it was observed that the IRF7^ΔF^ mice developed far more severe cardiac fibrosis with much inferior post-infarct heart function than the IRF7^f/f^ mice (Appendix Fig. S4).

Alternatively, IRF7 was deleted specifically in myofibroblasts (IRF7^ΔΜF^) by crossing the IRF7^f/f^ mice to the *Postn*-Cre^ERT2^ mice followed by the TAC procedure to induce cardiac fibrosis (Fig. 2L). Again, cardiac hypertrophy following the surgery appeared to be comparable in the IRF7^ΔΜF^ mice and the control mice (Fig. 2M–Q). However, cardiac fibrosis, as assessed by histological staining (Fig. 2R), hydroxyproline quantification (Fig. 2S), and expression levels of myofibroblast markers (Fig. 2T), was more severe in the IRF7^ΔΜF^ mice than in the control mice. Concordantly, further dampening of the heart function was observed in the IRF7^ΔΜF^ mice (Fig. 2U,V). The effect of myofibroblast-specific IRF7 on cardiac fibrosis was verified in a second model in which it was observed that the IRF7^ΔΜF^ mice displayed more rampant cardiac fibrosis and worsened heart function following myocardial infarction (Appendix Fig. S5).

### IRF7 overexpression therapeutically corrects cardiac fibrosis and averts heart failure

**Figure 3:**
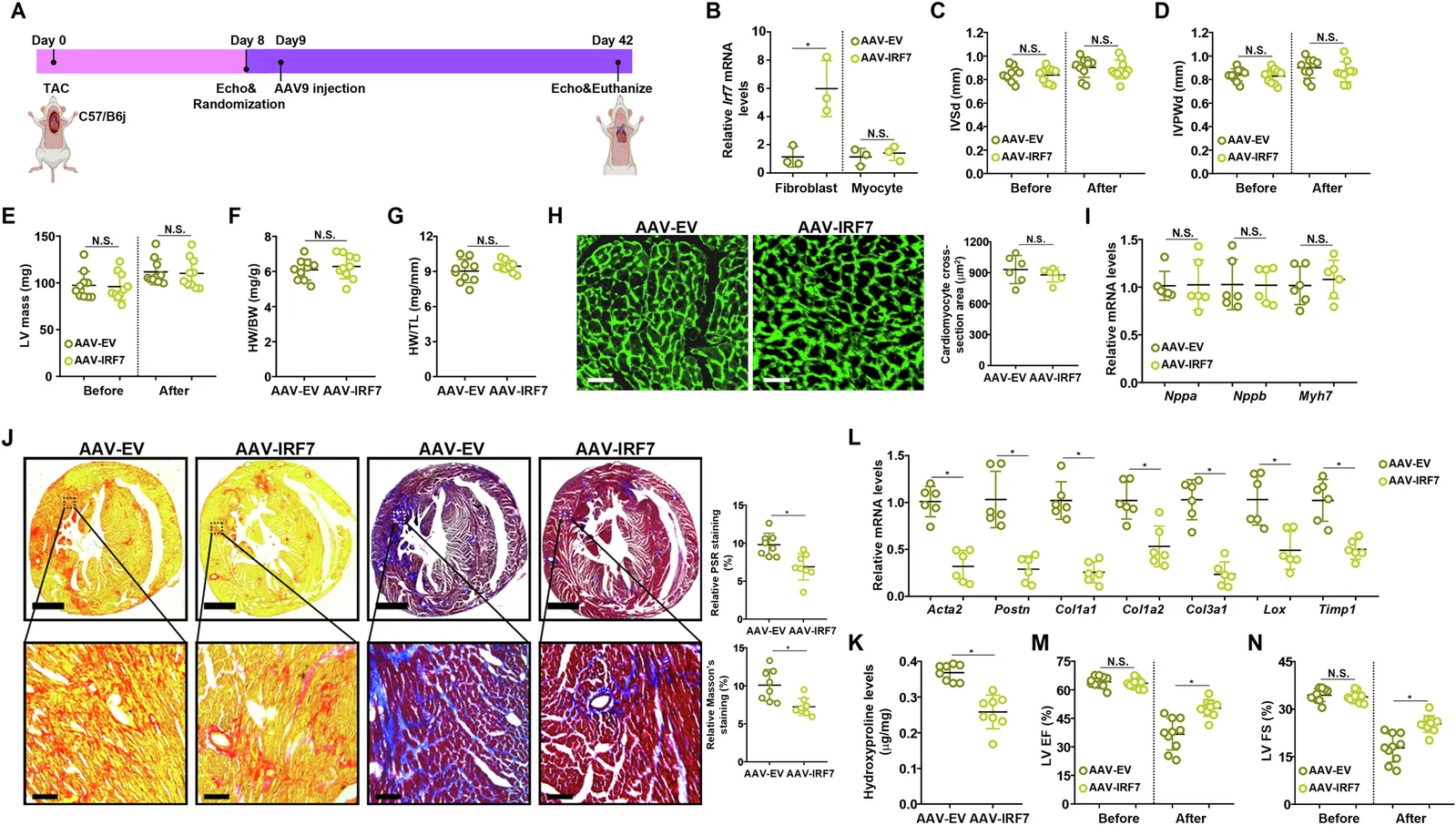
IRF7 overexpression therapeutically corrects cardiac fibrosis and averts heart failure. (**A**–**N**) C57/B6j mice were subjected to the TAC procedure followed by tail vein injection of AAV9 carrying an IRF7 vector (AAV-IRF7) or an empty vector (AAV-EV). Scheme of protocol (**A**). Primary cardiac fibroblasts and myocytes were isolated, and IRF7 expression was examined by qPCR (**B**). IVSd (**C**). IVWPd (**D**). LV mass (**E**). Heart weight versus body weight ratio (**F**). Heart weight versus tibia length ratio (**G**). Paraffin sections were stained with WGA (**H**). Pro-hypertrophic markers were examined by qPCR (**I**). Paraffin sections were stained with PicroSirius Red or Masson’s Trichrome (**J**). Hydroxyproline levels (**K**). Myofibroblast markers were examined by qPCR (**L**). LV EF (**M**). LV FS (**N**). *N* = 10 mice for the TAC groups. Scale bar, 1 mm (upper panel) and 50 μm (bottom panel). Data are expressed as mean ± SD. *P <0.05, two-tailed Student’s test (**J**–**L**) or one-way ANOVA with post hoc Scheffe’s test (the rest). Exact *P* values are reported in Appendix Table S4. Source data are available online for this figure.

In order to provide proof-of-concept for the therapeutic potential of targeting IRF7, the following experiments were performed. C57/B6j mice were subjected to the TAC procedure to induce cardiac fibrosis and heart failure. On day 8 after the surgery when the mice already developed cardiac hypertrophy but preserved heart function echocardiography was conducted for randomization.  Then AAV9 carrying an IRF7 cDNA driven by the *Postn* promoter to ensure myofibroblast-specific expression was injected via tail vein and the mice were sacrificed 5 weeks later (Fig. 3A). QPCR data showed that IRF7 expression was significantly upregulated (over 6xfold) in cardiac fibroblasts isolated from the AAV-IRF7 injected mice compared to the AAV-EV injected mice whereas IRF7 expression was comparable in the cardiomyocytes (Fig. 3B). Western blotting further confirmed the expression of ectopic IRF7 in heart tissues (Appendix Fig. S6). It was evident that IRF7 overexpression did not affect the hypertrophic response as measured by heart chamber size (Fig. 3C–E), normalized heart weight (Fig. 3F,G), WGA staining of paraffin heart section (Fig. 3H), and expression of pro-hypertrophic markers (Fig. 3I). On the contrary, IRF7 overexpression decelerated the development of cardiac fibrosis as assessed by PSR/Masson’s staining (Fig. 3J), cardiac hydroxyproline level (Fig. 3K), and expression of myofibroblast markers (Fig. 3L). Consequently, between the time of AAV9 injection and the time of euthanasia, the  AAV-EV mice developed heart failure as evidenced by a massive decline of LV EF and LV FS, whereas heart failure was largely averted in the AAV-IRF7 mice (Fig. 3M,N). These data collectively suggest that post-injury intervention with IRF7-carrying AAV could potentially rescue heart failure, possibly by averting adverse ventricular remodeling.

### Hexokinase 1 is a novel downstream target for IRF7 in cardiac fibroblasts

**Figure 4:**
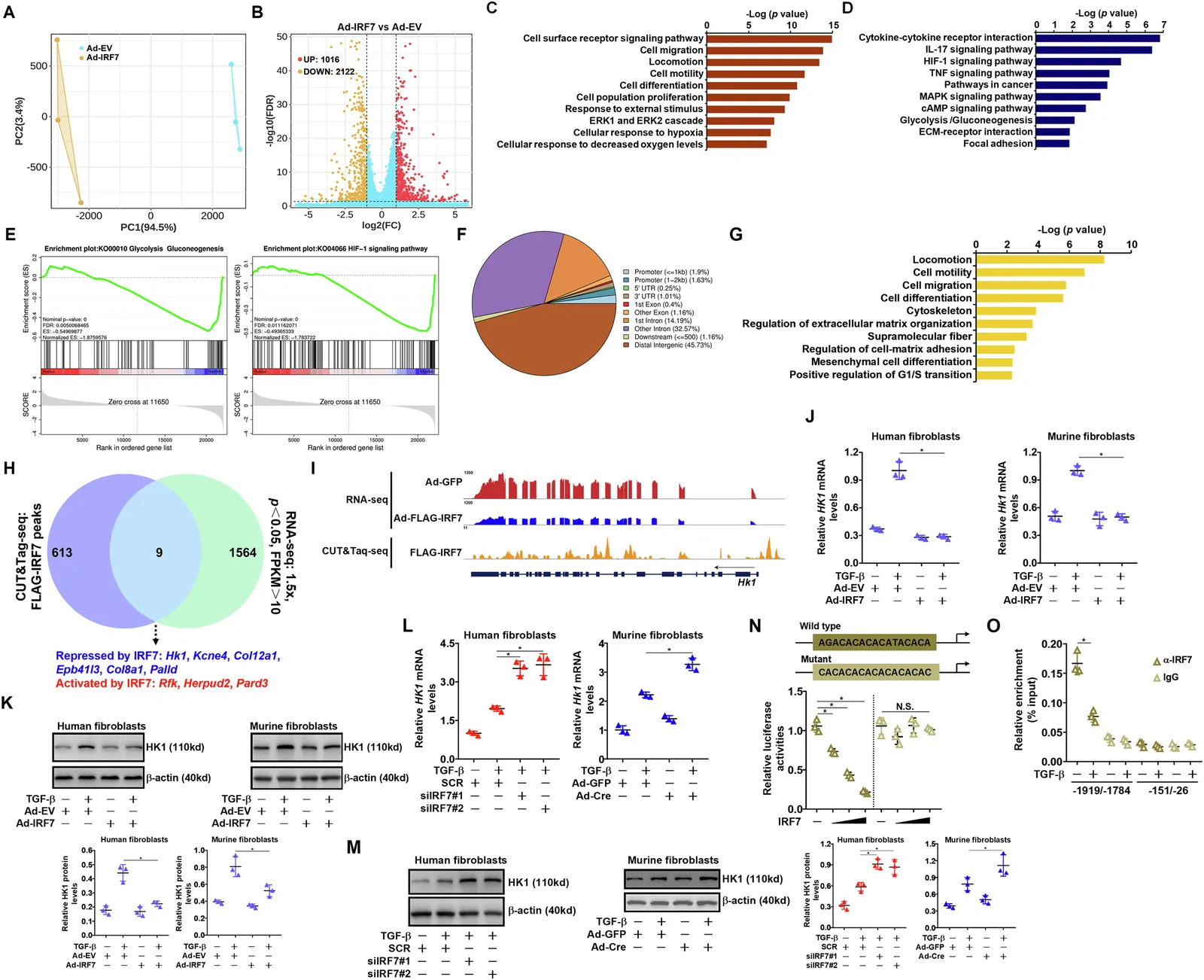
Hexokinase 1 is a novel downstream target for IRF7 in cardiac fibroblasts. (**A**–**E**) Primary murine cardiac fibroblasts were transduced with Ad-EV or Ad-FLAG-IRF7 followed by treatment with TGF-β (5 ng/ml) for 24 h. RNA-seq was performed as described in Methods. PCA plot (**A**). Volcano plot (**B**). GO analysis (**C**). KEGG analysis (**D**). Gene set enrichment analysis (**E**). (**F**–**H**) Primary cardiac fibroblasts were transduced with Ad-FLAG-IRF7 followed by treatment with TGF-β (5 ng/ml) for 24 h. CUT&Taq-seq was performed with anti-FLAG as described in “Methods”. Pie chart of genomic distributions of the FLAG-IRF7 peaks (**F**). GO analysis (**G**). Venn diagram (**H**). (**I**) CUT&Tag tracks of FLAG-IRF7 signals and RNA-Seq tracks of the read coverage surrounding the *Hk1* gene loci. (**J**, **K**) Primary human and murine cardiac fibroblasts were transduced with Ad-EV or Ad-FLAG-IRF7 followed by treatment with TGF-β (5 ng/ml) for 24 h. HK1 expression was examined by qPCR and western blotting. (**L, M**) Primary human cardiac fibroblasts were transfected with indicates siRNAs followed by treatment with TGF-β (5 ng/ml) for 24 h. Alternatively, primary murine cardiac fibroblasts isolated from the IRF7^f/f^ mice were transduced with Ad-GFP or Ad-Cre followed by treatment with TGF-β (5 ng/ml) for 24 h. HK1 expression was examined by qPCR and western blotting. (**N**) Wild-type and mutant HK1 promoter–luciferase constructs were transfected into HEK293 cells with or without IRF7. Luciferase activities were normalized by protein concentration and GFP fluorescence. (**O**) Primary murine cardiac fibroblasts were treated with or without TGF-β (5 ng/ml). ChIP assay was performed with an anti-IRF7 antibody or IgG. *N* = 3 biological replicates. Data are expressed as mean ± SD. *P <0.05, one-way ANOVA with post hoc Scheffe´s test. Exact *P* values are reported in Appendix Table S4. Source data are available online for this figure.

To provide mechanistic insights underlying IRF7-mediated suppression of cardiac fibrosis, we took advantage of an integrated transcriptomic approach combining RNA-seq and CUT&Tag-seq. To this end, primary murine cardiac fibroblasts were transduced with adenoviral IRF7 or an empty vector (EV), treated with TGF-β, and then subjected to RNA-seq analysis. IRF7 overexpression overhauled the cellular transcriptome resulting in more than 2000 genes being down-regulated and more than 1000 genes being upregulated (Fig. 4A,B). GO analysis indicated that the top pathways influenced by IRF7 included not only those might regulate cell behavior characteristic of fibroblast–myofibroblast transition (e.g., “Cell migration” and “Cell population prolferation”) but those involved in cellular response to hypoxia (Fig. 4C). KEGG analysis confirmed that two pathways that regulate cellular response to hypoxia, “HIF-1 signaling pathway” and “glycolysis/gluconeogenesis”, were among the top ten that were influenced by IRF (Fig. 4D). Geneset enrichment analysis also pointed to an inverse correlation between IRF7 overexpression and dampened response to hypoxia (Fig. 4E). Primary murine cardiac fibroblasts were then transduced with adenoviral FLAG-IRF7 followed by CUT&Tag with an anti-FLAG antibody. As shown in Fig. 4F, a majority of the FLAG-IRF7 peaks (45.73%) were detected in the distal intergenic regions, whereas a small fraction of these peaks (3.53%) were detected in the promoter regions. The genes whose loci were occupied by FLAG-IRF7 mostly were involved in regulating characteristic behaviors, proliferation and migration for instance, of fibroblasts undergoing trans-differentiation (Fig. 4G). A combinatorial analysis uncovered a total of 9 genes, whose expression levels were altered by IRF7 overexpression and whose loci were occupied by FLAG-IRF7; Hexokinase 1 (HK1) ranked top of the list and was the only gene with a direct role in cellular response to hypoxia (Fig. 4H,I).

Compared to functionally similar ortholog HK2 that is preferentially expressed in cancer cells, HK1 is thought to be ubiquitously expressed, although a recent study indicates that hepatic stellate cell-derived HK1 can be transmitted via exosomal vesicles to hepatocellular carcinoma cells to fuel malignant expansion (Chen et al, 2022b). HK1 expression was upregulated in cardiac fibroblasts by TGF-β at both mRNA and protein levels (Appendix Fig. S7). IRF7 overexpression repressed (Fig. 4J,K), whereas IRF7 deletion amplified (Fig. 4L,M), HK1 induction by TGF-β treatment in both murine and human cardiac fibroblasts. HK1 expression was also inducible by Ang II treatment, whereas IRF7 deletion further enhanced the induction (Appendix Fig. S8). A conserved IRF binding motif (AGACACACACATACACA) was identified to be located around -1800 relative to the transcription start site of the *Hk1* gene; when the HK1 promoter (−2000/ + 50) was fused to a reporter and transfected into HEK293 cells, IRF7 overexpression significantly repressed the reporter activity only when the IRF7 motif was present (Fig. 4N). More importantly, IRF7 binding was detected surrounding the IRF motif of the *Hk1* promoter in quiescent fibroblasts by ChIP assay, whereas TGF-β treatment significantly dampened IRF7 binding (Fig. 4O).

To further demonstrate the functional interplay between IRF7 and HK1 in regulating fibroblast–myofibroblast transition, the following experiments were performed. In cardiac fibroblasts, IRF7 knockdown enhanced TGF-β-induced transition to a myofibroblast-like phenotype, which was blunted by simultaneous depletion of HK1 (Appendix Fig. S9). In contrast, attenuation of fibroblast–myofibroblast transition by IRF7 overexpression was completely relieved by simultaneous overexpression of HK1 (Appendix Fig. S10).

### HK1 deletion attenuates cardiac fibrosis in mice

**Figure 5:**
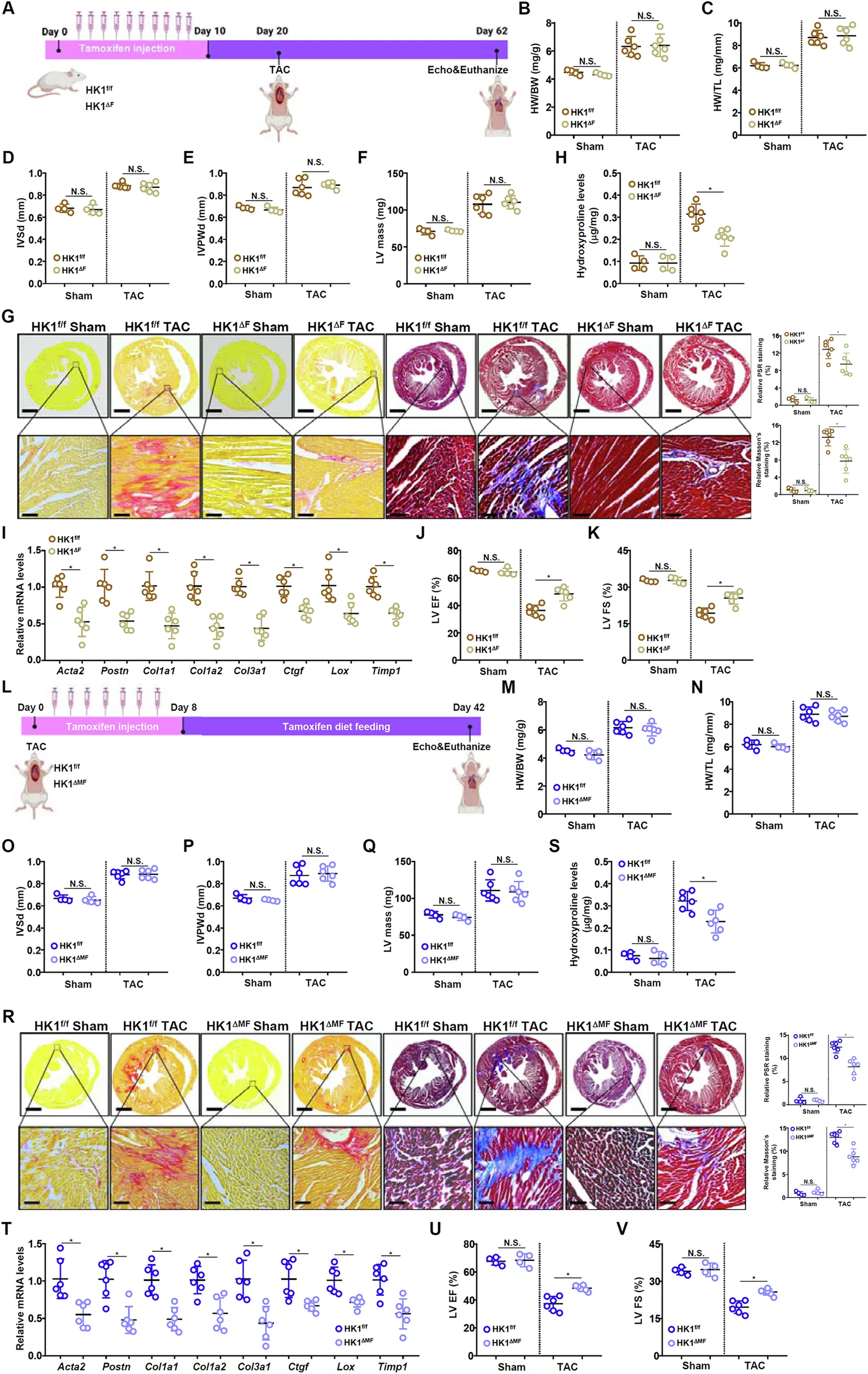
HK1 deletion attenuates cardiac fibrosis in mice. (**A**–**K**) Fibroblast conditional HK1 knockout (HK1^ΔF^) and wild-type mice were subjected to the TAC procedure and euthanized 6 weeks after the surgery. Scheme of protocol (**A**). Heart weight versus body weight ratio (**B**). Heart weight versus tibia length ratio (**C**). IVSd (**D**). IVWPd (**E**). LV mass (**F**). Paraffin sections were stained with PicroSirius Red or Masson’s Trichrome (**G**). Hydroxyproline levels (**H**). Myofibroblast markers were examined by qPCR (**I**). LV EF (**J**). LV FS (**K**). (**L**–**V**) Myofibroblast conditional HK1 knockout (HK1^ΔMF^) and wild-type mice were subjected to the TAC procedure and euthanized 6 weeks after the surgery. Scheme of protocol (**L**). Heart weight versus body weight ratio (**M**). Heart weight versus tibia length ratio (**N**). IVSd (**O**). IVWPd (**P**). LV mass (**Q**). Paraffin sections were stained with PicroSirius Red or Masson’s Trichrome (**R**). Hydroxyproline levels (**S**). Myofibroblast markers were examined by qPCR (**T**). LV EF (**U**). LV FS (**V**). *N* = 4–6 mice for each group. Scale bar, 1 mm (upper panel) and 50 μm (bottom panel). Data are expressed as mean ± SD. *P <0.05, two-tailed Student’s test (**I**, **T**) or one-way ANOVA with post hoc Scheffe’s test (the rest). Exact *P* values are reported in Appendix Table S4. Source data are available online for this figure.

To assign a more definitive role for HK1 in fibroblast activation and cardiac fibrosis, the HK1^f/f^ mice were generated by floxing 2^nd^ exon of the *Hk1* gene. Next, the HK1^f/f^ mice were bred with the *Col1a2*-Cre^ERT2^ mice to generate fibroblast-specific HK1 knockout mice (HK1^ΔF^); both the HK1^ΔF^ mice and the HK1^f/f^ mice were subjected to the TAC procedure to evaluate the effect of HK1 deletion on cardiac fibrosis (Fig. 5A). HK1 deletion in fibroblasts did not significantly alter cardiac hypertrophy (Fig. 5B–F) but substantially dampened cardiac fibrosis (Fig. 5G–I) and rescued heart function (Fig. 5J,K). Similarly, a mouse strain harboring myofibroblast-specific HK1 deletion (HK1^ΔMF^) was generated by crossing the HK1^f/f^ mice with the *Postn*-Cre^ERT2^ mice; both the HK1^ΔMF^ mice and the HK1^f/f^ mice were then subjected to the TAC procedure to induce cardiac fibrosis and heart failure (Fig. 5L). Again, no significant difference in cardiac hypertrophy between the HK1^ΔMF^ mice and the HK1^f/f^ mice was observed (Fig. 5M–Q), indicating that myofibroblast-derived HK1 might be dispensable for the hypertrophic response in cardiomyocytes. However, significantly less extensive cardiac fibrosis was recorded in the HK1^ΔMF^ mice compared to the HK1^f/f^ mice (Fig. 5R–T). As a result, the HK1^ΔMF^ mice displayed better heart function during the decompensatory stage of heart failure (Fig. 5U,V). Taken together, these data support an essential role for HK1 to modulate fibroblast/myofibroblast phenotype and promote cardiac fibrosis.

### HK1 links histone lactylation to fibroblast activation

**Figure 6:**
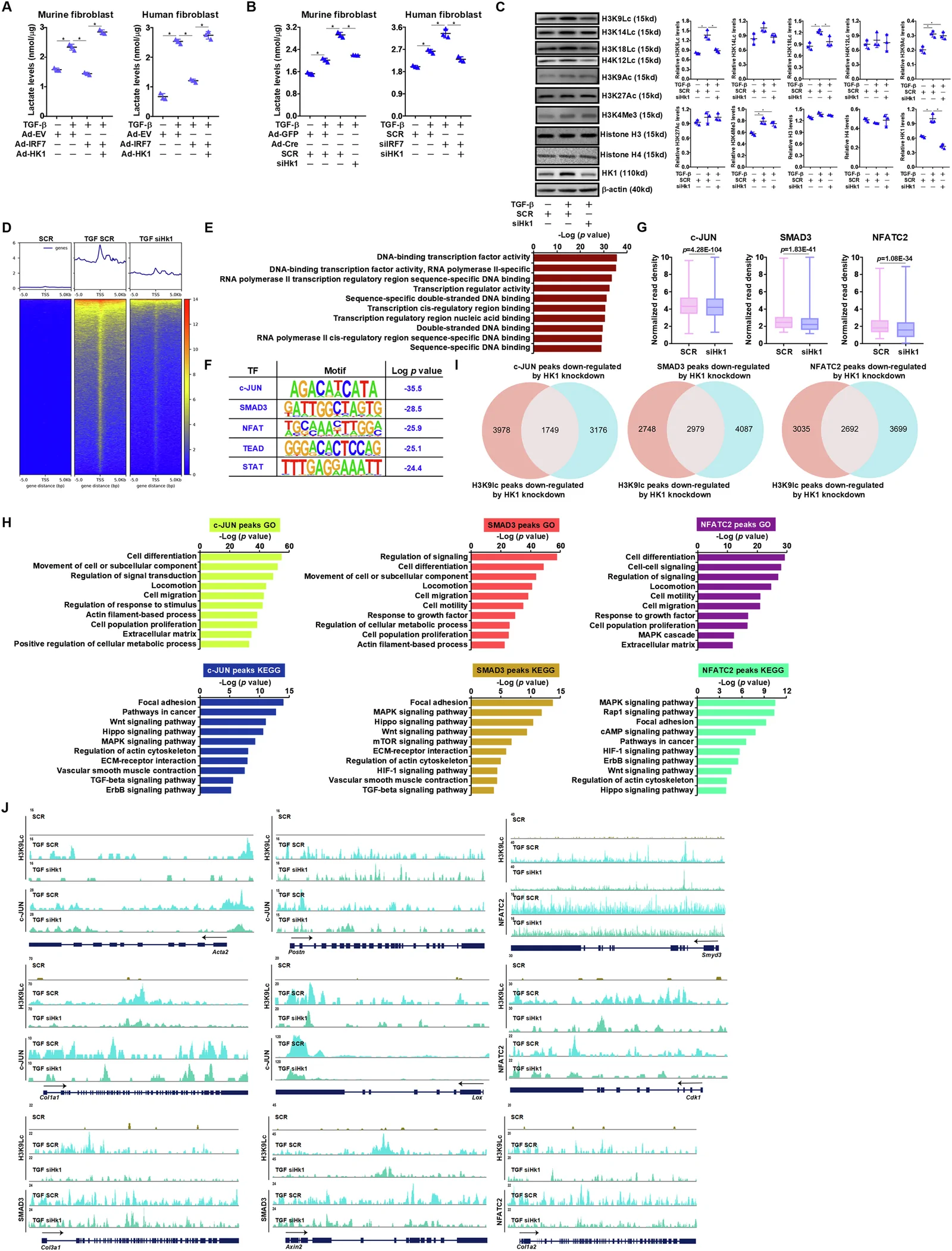
HK1 links histone lactylation to fibroblast activation. (**A**) Murine and human primary cardiac fibroblasts were transduced with indicated adenovirus followed by treatment with TGF-β (5 ng/ml) for 24 h. Cellular lactate levels were measured by coloriometry. (**B**) Murine and human primary cardiac fibroblasts were transfected with indicated siRNAs followed by treatment with TGF-β (5 ng/ml) for 24 h. Cellular lactate levels were measured by coloriometry. (**C**) Murine primary cardiac fibroblasts were transfected with siRNA targeting HK1 followed by treatment with TGF-β (5 ng/ml) for 24 h. Histone lactylation was examined by Western blotting. N = 3 biological replicates. Data are expressed as mean ± SD. *P <0.05, one-way ANOVA with post hoc Scheffe´s test. Exact *P* values are reported in Appendix Table S4. (**D**–**F**) Murine primary cardiac fibroblasts were transfected with siRNA targeting HK1 followed by treatment with TGF-β (5 ng/ml) for 24 h. CUT&Tag-seq was performed with anti-lactyl H3K9 antibody. Heatmap (**D**). KEGG analysis. *P* values were calculated with the hypergeometric test (**E**). HOMER analysis (**F**). (**G**–**J**) Murine primary cardiac fibroblasts were transfected with siRNA targeting HK1, followed by treatment with TGF-β (5 ng/ml) for 24 h. CUT&Tag-seq was performed with anti-c-JUN, anti-SMAD3, and anti-NFATC2. Box-and-whisker plot. The center line represents the median (50th percentile); the box spans the 25th to 75th percentiles; the whiskers extend to the overall minimum and overall maximum of the data. No individual data points are shown. *N* = 1 biological repeat. Two-tailed Student’s test. (**G**). GO and KEGG analyses. *P* values were calculated with the hypergeometric test (**H**). Venn diagram (**I**). CUT&Tag tracks of c-JUN/SMAD3/NFATC2 signals and RNA-Seq tracks of the read coverage surrounding the *Acta2*, *Postn*, Col1a1, Col3a1, Axin2, and *Slc40a1* loci. Source data are available online for this figure.

One of the best characterized functions for HK1 is to control the cellular pool of lactate. Indeed, lactate accumulation in fibroblasts was dually regulated by IRF7 and HK1: IRF7 overexpression depleted cellular lactate, which was offset by HK1 overexpression (Fig. 6A); IRF7 knockdown expanded the cellular pool of lactate, which was negated by HK1 knockdown (Fig. 6B). Histone lactylation is a recently recognized epigenetic mechanism that regulates gene transcription. As shown in Fig. 6C, TGF-β treatment increased lactylation on several different histone H3 lysine residues, all of which were reversed by HK1 knockdown. Acetylated H3K9/H3K27 (Shahbazian and Grunstein, 2007) and trimethylated H3K4 (Shilatifard, 2012) are traditionally considered markers that label actively transcribed chromatin. Notably, whereas both H3K9Ac and H3K4Me3 were significantly upregulated by TGF-β treatment, HK1 knockdown did not alter the status of either modification (Fig. 6B). On the other hand, H3K27Ac status was altered by neither TGF-β treatment nor HK1 knockdown (Fig. 6B). Because H3K9 lactylation (H3K9lc) underwent the most significant changes in response to TGF-β treatment and HK1 knockdown, we focused on this specific form of modification for the remainder of the study.

CUT&Tag-seq showed that TGF-β treatment led to massive increase in genomewide occupancies of H3K9lc, which was significantly dampened by HK1 knockdown (Fig. 6D). KEGG analysis indicated that TGF-β-induced, HK1-dependent, and locus-specific dynamic changes in H3K9 lactylation predominantly regulated transcription by influencing transcription factor activities (Fig. 6E). HOMER analysis further showed that the top three transcription factors influenced by HK1-dependent H3K9lc were c-JUN, SMAD3, and NFATC2 (Fig. 6F). Next, we tackled the question as to whether genomewide binding of c-JUN, SMAD3, and/or NFATC2 would be altered by HK1-dependent H3K9 lactylation. As shown in Fig. 6G, chromatin bindings of c-JUN, SMAD3, and NFATC2, as measured by normalized peak intensity per CUT&Tag-seq, were all significantly weakened by HK1 knockdown. GO and KEGG analyses indicated that differential c-JUN/SMAD3/NFATC2 peaks annotated genes that were involved in signature FMyT-related processes (Fig. 6H). In addition, a significant fraction of overlap was identified between H3K9lc-occupied peaks and c-JUN/SMAD3/NFATC2 peaks (Fig. 6I). Indeed, several myofibroblast markers, including *Acta2*, *Postn*, *Col1a1*, *Col1a2*, *Col3a1*, *Lox*, *Axin2*, *Smyd3*, and *Cdk1*, appeared to be demarcated by H3K9lc, which might serve as prerequisite for the recruitment /binding of c-JUN/SMAD3/NFATC2 (Fig. 6J).

### HK1 inhibition attenuates cardiac fibrosis in mice

**Figure 7:**
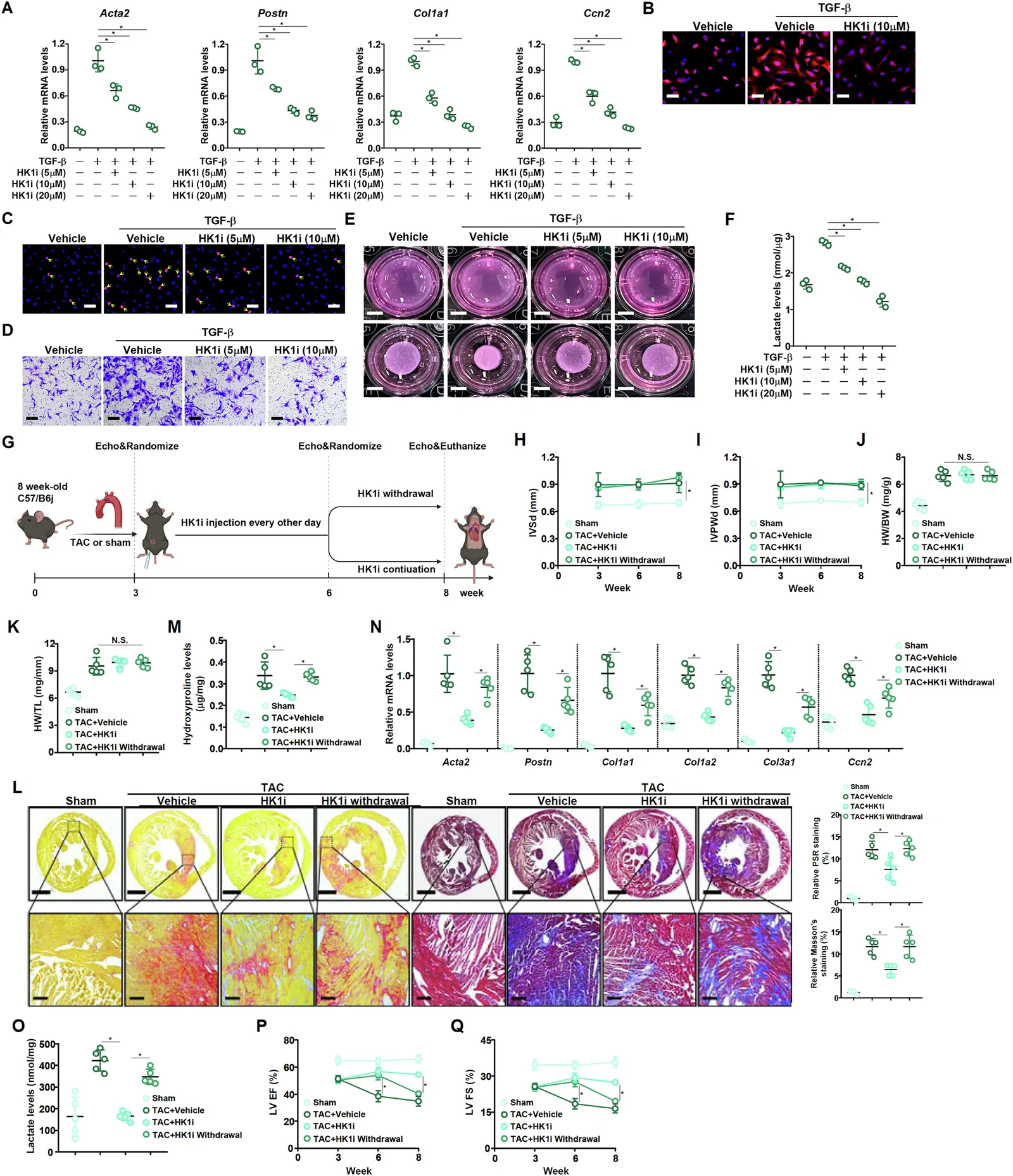
HK1 inhibition attenuates cardiac fibrosis in mice. (**A**–**F**) Primary murine cardiac fibroblasts were treated with TGF-β (5 ng/ml) in the presence or absence of an HK1 inhibitor (HK1i) for 24 h. Myofibroblast markers were examined by qPCR (**A**) and immunofluorescence staining (**B**). EdU incorporation assay (**C**). Transwell assay (**D**). Collagen contraction assay (**E**). Scale bar = 50 μm (**B**–**D**) and Scale bar = 1 cm (**E**). Intracellular Lactate levels (**F**). *N* = 3 biological replicates. Data are expressed as mean ± SD. *P <0.05, one-way ANOVA with post hoc Scheffe´s test. Exact *P* values are reported in Appendix Table S4. (**G**–**Q**) C57/B6j mice were subjected to the TAC procedure followed by administration with HK1i. Scheme of protocol (**G**). Heart weight versus body weight ratio (**H**). Heart weight versus tibia length ratio (**I**). IVSd (**J**). IVWPd (**K**). Paraffin sections were stained with PicroSirius Red or Masson’s Trichrome (**L**). Hydroxyproline levels (**M**). Myofibroblast markers were examined by qPCR (**N**). Cardiac lactate levels (**O**). LV EF (**P**). LV FS (**Q**). *N* = 5 mice for each group. Scale bar, 1 mm (upper panel) and 50 μm (bottom panel). *P <0.05, one-way ANOVA with post hoc Scheffe’s test. Exact *P* values are reported in Appendix Table S4. Source data are available online for this figure.

The translational potential of targeting HK1 for the intervention of cardiac fibrosis and heart failure was explored by harnessing a recently reported small-molecule HK1 inhibitor (HK1i). HK1i treatment dose-dependently repressed the induction of myofibroblast markers (Fig. 7A,B) and blocked the augmentation of proliferation (Fig. 7C), migration (Fig. 7D), and contraction (Fig. 7E) in primary murine cardiac fibroblasts. Of note, HK1i treatment also abolished the accumulation of intracellular lactate (Fig. 7F). Similar observations were made in primary human cardiac fibroblasts (Appendix Fig. S11).

Next, C57/B6j mice were subjected to the TAC procedure followed by administration with HK1i (Fig. 7G). HK1i administration did not significantly alter cardiac hypertrophy (Fig. 7H–K). On the other hand, HK1i administration significantly dampened cardiac fibrosis (Fig. 7L–N). It was noteworthy that the effect of HK1i appears to be reversible because, following its withdrawal, the magnitude of cardiac fibrosis returned to control levels (Fig. 7L–N). Cardiac lactate levels and post-surgical heart function mirrored the pattern of cardiac fibrosis: HK1i administration decreased cardiac lactate levels and improved heart function, both of which were normalized by HK1i withdrawal (Fig. 7O–Q).

### Relevance of the IRF7-HK1 axis in humans

**Figure 8:**
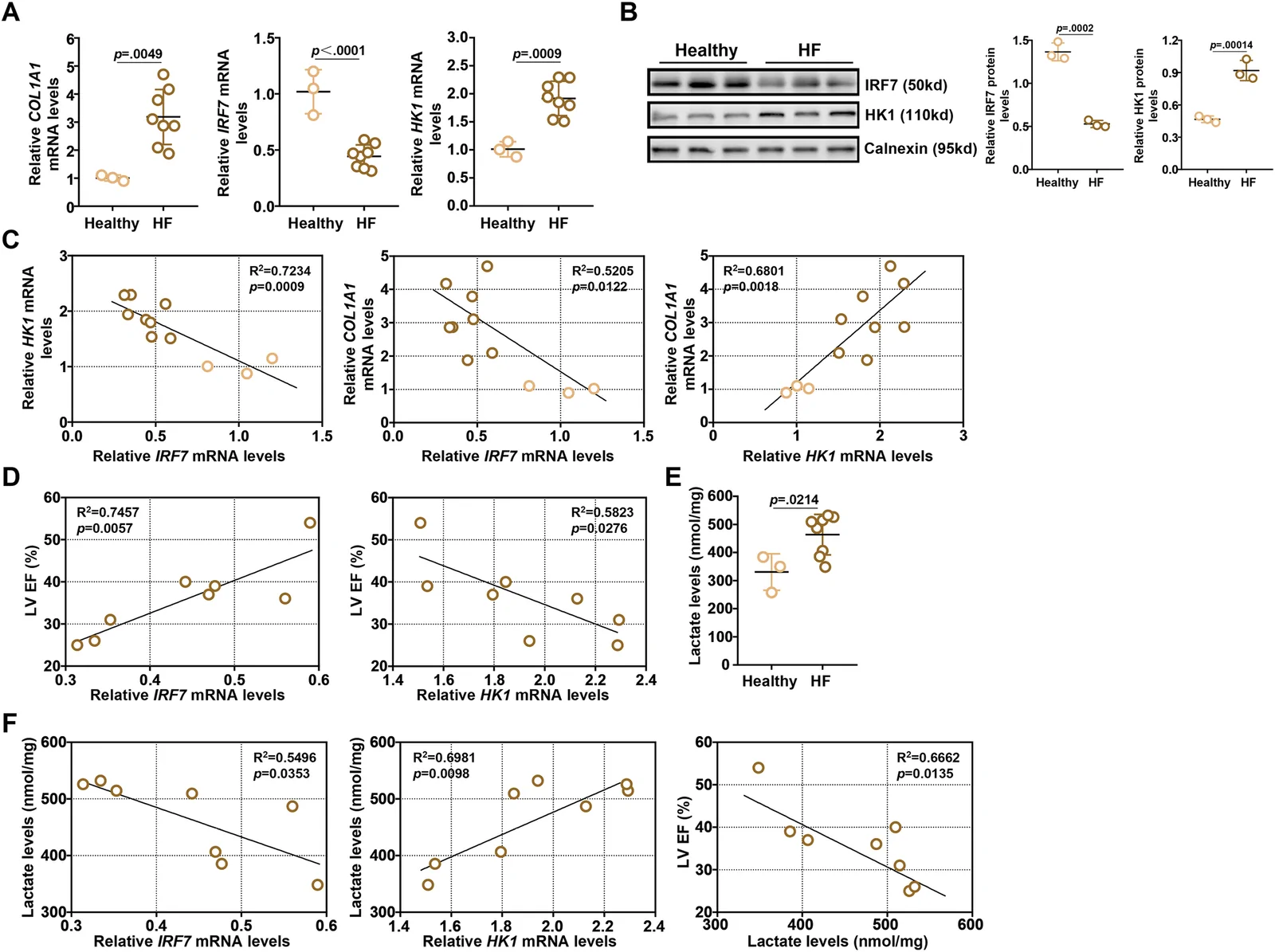
Relevance of the IRF7-HK1 axis in humans. (**A**, **B**) Gene expression levels in heart tissues were examined by qPCR and Western blotting. *N* = 3 for healthy individuals and *N* = 9 for HF patients. Data are expressed as mean ± SD. *P <0.05, two-tailed Student’s test. Exact *P* values are reported in Appendix Table S4. (**C**) Correlation between IRF7 expression, HK1 expression, and myofibroblast marker expression. *P* values were calculated with Pearson’s chi-squared test. (**D**) Correlation between IRF7/HK1 and heart function. *P* values were calculated with Pearson’s chi-squared test. (**E**) Lactate level in heart tissues. *N* = 3 for healthy individuals and *N* = 9 for HF patients. Data are expressed as mean ± SD. *P <0.05, two-tailed Student’s test. Exact *P* values are reported in Appendix Table S4. (**F**) Correlation between lactate level and heart function. *P* values were calculated with Pearson’s chi-squared test. Source data are available online for this figure.

Finally, the relevance of our findings in human pathology was evaluated. An upregulation of HK1 expression and a down-regulation of IRF7 were detected in the cardiac tissues from HF patients compared to the healthy controls (Fig. 8A,B). An inverse correlation was identified between IRF7 expression and HK1 expression (Fig. 8C). Whereas IRF7 was inversely correlated with the myofibroblast marker HK1 was positively correlated with the myofibroblast marker (Fig. 8C). Importantly, low IRF7 expression and high HK1 expression appeared to predict worsened heart function (Fig. 8D). In addition, cardiac lactate levels were elevated in HF patients (Fig. 8E). Further, lactate levels were a function of both IRF7 expression and HK1 expression in the heart and could be used to predict heart function in HF patients (Fig. 8F). These data, when taken together, demonstrate that the IRF7-HK1 axis might be operative in HF pathogenesis in humans.

## Discussion

Fibroblast activation and subsequent excessive production of ECM proteins by myofibroblasts represent a hallmark in myocardial fibrosis that ultimately leads to heart failure. In this report, we detail a fibroblast/myofibroblast-autonomous role for IRF7 in cardiac fibrosis. Our observation is consistent with a previous study showing that mice with cardiomyocyte-specific IRF7 overexpression display attenuated cardiac fibrosis compared to the wild-type littermates in response to pressure overload (Jiang et al, 2014). However, because it has also been observed that IRF7 overexpression in cardiomyocytes antagonizes cardiac hypertrophy, the fibrosis phenotype can be interpreted as secondary to myocardial injury (Jiang et al, 2014). Whereas both our study and the Jiang et al study (Jiang et al, 2014) suggest that boosting IRF7 activity, either in fibroblast/myofibroblast or in cardiomyocyte, may be beneficial for preserving heart function, two recent studies point to the opposite direction. Chen et al propose that the E3 ubiquitin ligase WWP2 promotes cardiac fibrosis through non-degradation ubiquitination of IRF7 in macrophages, which presumably activates the transcription of CCL5 to induce infiltration of Ly6c^high^ monocytes (Chen et al, 2022a). In another study by Allen-Gondringer et al (Allen-Gondringer et al, 2023), deletion of the actin-binding protein Profilin 1 in vascular endothelial cells leads to spontaneous cardiac fibrosis and heart failure accompanied by a gene expression signature reminiscent of hyper-activated IRF7. A causal relationship between macrophage-specific/endothelial-specific IRF7 and cardiac fibrosis, however, has yet to be established. Indeed, both pro-fibrotic and anti-fibrotic roles for IRF7 have previously been documented (Ibrahim et al, 2016; Sermasathanasawadi et al, 2008; Wu et al, 2019). Therefore, IRF7 likely shapes the fibrogenic response in a context- and cell lineage–dependent manner, indicating that any translational strategies targeting IRF7 in heart failure should be carefully and precisely tailored.

Through integrated transcriptomic screening, HK1 was identified as a novel IRF7 target in fibroblasts. Several recent studies have defined HK1 as a potential biomarker and/or regulator of cardiac fibrosis and heart failure. For instance, meta-analysis of multi-omics data points to an association of elevated HK1 expression with adverse ventricular remodeling and heart failure in rodents and in humans (Koop et al, 2019; Rashid et al, 2024). In addition, HK1 upregulation seems to account for, at least in part, the reduction of ejection fraction in COX2^−/−^ rats (Wan et al, 2021). Our observation that genetic and pharmaceutical manipulation of HK1 attenuates cardiac fibrosis and rescues heart failure substantiates these previous findings. The notion that HK1 may represent a bona fide universal orchestrator of tissue fibrosis remains to be ascertained. The Wu group has recently reported that HK1 transferred via exosomal vesicle from hepatic stellate cells (HSCs, or hepatic myofibroblasts) can promote liver tumorigenesis by reprogramming metabolism in hepatocytes; however, targeted deletion of HK1 in HSCs (hepatic myofibroblasts) did not alter liver fibrosis (Chen et al, 2022b). However, the HSC-specific HK1 deletion mice in the Chen et al study were generated using the *Gfap*-Cre driver (Chen et al, 2022b), which might lead to non-specific and inefficient HSC-specific gene targeting, contrary to the *Lrat*-Cre driver. Therefore, the possibility that HK1 functions as a regulator of myofibroblast maturation in the liver cannot be excluded. Further studies are needed to clarify HK1’s role and to establish the broader significance of our findings.

One of the most exciting findings is that HK1-dependent histone H3K9 lactylation functions as a driving force of FMyT and cardiac fibrosis. A landmark study by Rho et al stipulates that HK2-mediated H3K18 lactylation plays a permissive role in trans-activating key pro-fibrogenic genes (e.g., *Acta2*, *Col1a1*, and *Timp1*) to fuel myofibroblast maturation in the course of liver fibrosis (Rho et al, 2023). While our findings corroborate those of Rho et al and suggest that histone lactylation may act as a rate-limiting step in fibrogenesis, we further demonstrate that specific histone states are linked to locus-dependent transcription factor binding and recruitment. The Evans group has previously shown that Vitamin D receptor activation attenuates liver fibrosis by triggering histone deacetylation to remove the pro-fibrogenic transcription factor SMAD3 from the chromatin (Ding et al, 2013). Our finding thus adds support to the notion that chromatin status is bar-coded by differential histone modifications, including acetylation and lactylation, to dictate cue-specific transcription events. However, certain caveats in this model warrant further attention. Although we focused on H3K9Lc in the present study, lactylation of other histone lysine residues underwent similar, albeit less prominent, dynamic changes in an HK1-dependent manner. Of note, bioinformatic analysis indicates that the binding of c-JUN/SMAD3/NFATC2 largely (but not entirely) coincides with the H3K9Lc signals across the chromatin, suggesting that other histone modifications may contribute to this process. In addition, there is a growing list of non-histone proteins (e.g., transcription factors) subjected to lysine lactylation with an increasingly recognized role in regulating gene expression (Shi et al, 2025; Yu et al, 2024). A screening and characterization in cardiac fibroblasts of these lactylated non-histone factors may provide further insights into the mechanism by which HK1 regulates FMyT and cardiac fibrosis.

Despite the advances offered by our study, several limitations may constrain its translational potential. First, chronic heart failure typically develops over the course of decades, the pathogenesis of which may not be fully recapitulated in the rodent models employed here. Additional in vivo studies are needed to further substantiate the main conclusions. Second, although we provide compelling evidence to implicate IRF7 as a pivotal regulator of cardiac fibrosis and heart failure, transcription factors have traditionally been regarded as challenging targets for drug development. Recent studies to target p53 (Duffy et al, 2022) and c-Myc (Rickman et al, 2018) for cancer treatment may serve as examples. Third, our finding that HK1-dependent histone H3K9 lactylation permits pro-fibrogenic transcription in cardiac fibroblasts further supports the idea that lactylation represents an important regulatory layer in transcriptional activation, as first proposed by the pioneering work of the Zhao laboratory (Zhang et al, 2019). Because lysine acetylation (Shahbazian and Grunstein, 2007) and H3K4 trimethylation (Shilatifard, 2012) have traditionally been considered the predominant forms of histone modifications, the relationship between lysine lactylation, lysine acetylation, and H3K4 trimethylation in the context of cardiac fibrosis and beyond remains an open question. Our data suggest that, under conditions in which H3K9 lactylation is altered in an HK1-dependent manner, neither H3K9/H3K27 acetylation nor H3K4 trimethylation is significantly affected. This finding, however, does not exclude the possibility of a crosstalk between lysine lactylation, acetylation, and methylation to program transcription in cardiac fibroblasts. Previous studies have already outlined several key differences between these modifications that include competition (the same lysine residue cannot be modified by acetylation and lactylation simultaneously), kinetics (lactylation occurs typically at a much slower pace than acetylation), and bioavailability of substrates (acetylation relies on intracellular acetyl-CoA, which can be derived from pyruvate at the expense of lactate consumption). These nuances should be further explored to not only conceptually advance the field but also facilitate the development of targeting strategies for disease treatment. Finally, the relatively small sample size of HF specimens used to validate our model introduces uncertainty and leaves room for alternative interpretations. Further validation in larger, independent cohorts across diverse disease contexts will be essential to strengthen these findings and may provide additional insights to inform translational strategies for the treatment of heart failure.

## Methods

**Table:** Reagents and tools table

Reagent/resource	Reference or source	Identifier or catalog number
**Experimental models**		
*Col1a2*-Cre^ERT2^	Jackson Lab	#029567
*Postn*-Cre^ERT2^	Jackson Lab	#029645
*Irf7* ^*f/f*^	Prof. Guoliang Xu	#T010975
*HK1* ^*f/f*^	Cyagen	#02895
**Recombinant DNA**		
Ad5-CMV-Cre-3FLAG-EGFP	Genechem	N/A
Ad5-CMV-3FLAG-EGFP	Genechem	N/A
Ad5-CMV-hHK1-3FLAG-EGFP	Zebrafish Biotech	N/A
Ad5-CMV-hIRF7-3FLAG-EGFP	Zebrafish Biotech	N/A
AAV9[ssAAV.periostin.mouse.Irf7-3FLAG-P2A-EGFP.WPRE3.SV40pA]	PackGene	N/A
AAV9[ssAAV.periostin.EGFP.WPRE3.SV40pA]	PackGene	N/A
**Antibodies**	This study	Table S1
**Oligonucleotides and other sequence-based reagents**		
PCR primers	This study	Table S2
siRNA	This study	Table S2
**Chemicals, enzymes, and other reagents**		
Human Cardiac Fibroblasts	Lifeline	#FC-0060
Dulbecco’s Modified Eagle’s Medium (DMEM)	Gibco	#11965092
Fetal bovine serum (FBS)	Gibco	#10270-106
Opti-MEM	Gibco	#31985070
Trypsin (0.25%)	Gibco	#25200072
Lipofectamine™ RNAiMAX Transfection Reagent	Invitrogen	#13778150
Click-iT™ Plus EdU Cell Proliferation Kit for Imaging, Alexa Fluor™ 594 dye	Invitrogen	#C10639
Oridonin	Selleck	#S2335
Collagen Type I Rat Tail	Corning	#354236
Corning 35 mm TC-treated Culture Dish	Corning	#430165
Corning 60 mm TC-treated Culture Dish	Corning	#430166
Corning 100 mm TC-treated Culture Dish	Corning	#430167
Tissue culture plate-6 well	Jetbiofil	#TCP011006
Tissue culture plate-12 well	Jetbiofil	#TCP011012
Tissue culture plate-48 well	Jetbiofil	#TCP011024
Cell lysis buffer for Western and IP	Beyotime	#P0013
Penicillin-Streptomycin Solution	Beyotime	#C0222
Hydroxyproline assay kit	solarbio	#BC0250
Masson’s Trichrome Stain Kit	solarbio	#G1346
Picrosirius RedStain Kit	Leagene	# DC0040
Recombinant Human TGF-beta 1 Protein	R&D	#7754-BH
Goat anti-Rabbit IgG (H + L) Cross-Adsorbed Secondary Antibody, Alexa Fluor™ 594	Thermofisher	#A-11012
Thermo Scientific PageRuler Plus	Thermofisher	#26620
Pierce BCA Protein Assay Kit	Thermofisher	#23225
Phenylmethylsulfonyl fluoride(PMSF)	Thermofisher	#36978
Tamxifen	Sigma	#T5648
DMSO	Sigma	#D8418
L-Lactate Assay Kit	Abcam	#AB65331
Hyperactive Universal CUT&Tag Assay Kit for Illumina Pro	vazyme	#TD904-01
VAHTS DNA Clean Beads	vazyme	#N411
TruePrep Index Kit V2 for Illumina	vazyme	#TD202
Hyperactive ATAC-Seq Library Prep Kit for Illumina	vazyme	#TD711
FastPure Cell/Tissue Total RNA Isolation Kit	vazyme	#RC101-01
HiScript II Q RT SuperMix for qPCR	vazyme	#R222-01
ChamQ SYBR Color qPCR Master Mix	vazyme	#Q411-02
**Software**		
GraphPad Prism9	GraphPad Software, Inc	RRID:SCR_002798
ImageJ-Fiji	https://imagej.net/software/fiji/	RRID:SCR_002285
Adobe Illustrator	Adobe Systems	RRID:SCR_010279

### Animals

All animal experiments were reviewed and approved by the Ethics Committee on Humane Treatment of Laboratory Animals of Nanjing Medical University (IACUC-2109023) and were performed in accordance with the ethical standards laid down in the 1964 Declaration of Helsinki and its later amendments. Unless specified, the animals were kept under constant environmental conditions with 12 h light/dark cycles and ad libitum access to food and water. *Col1a2*-Cre^ERT2^ mice (Khalil et al, 2017) and *Postn*-Cre^ERT2^ mice (Kanisicak et al, 2016) have been described previously. IRF7^f/f^ mice were generated by inserting LoxP sites to flank exon 1 and exon 4 of the *Irf7* gene. HK1^f/f^ mice were generated by inserting LoxP sites to flank exon 2 of the *Hk1* gene. All the strains were on a C57BL/6 J background. To induce Cre expression in the *Col1a2*-Cre^ERT2^ mice, tamoxifen (T2859, Sigma, St. Louis, MO, USA) was injected peritoneally (50 mg/kg) for 7 consecutive days, followed by a washing phase of 7 days as previously described (Katanasaka et al, 2024; Li and Bian, 2024). To induce Cre expression in the *Postn*-Cre^ERT2^ mice, tamoxifen was injected peritoneally (50 mg/kg) for 5 consecutive days, followed by maintaining the mice on a TAX-containing diet (130855, Inotiv, Lafayette, IN, USA) as previously described (Kanisicak et al, 2016; Khalil et al, 2017).

Cardiac fibrosis was induced by one of the following methods: (1) Permanent ligation of the left anterior descending coronary artery (LAD)(Yang et al, 2017). Briefly, 8–10-week-old male mice (25–27 g) were anesthetized with isoflurane, and a midline incision was created to expose the heart, and the artery was ligated with a 6-0 Prolene thread. The wound was then closed using a subcuticular suture. Infarct size was determined by triphenyltetrazolium chloride (TTC, Sigma-Aldrich) staining. Briefly, the hearts were removed and perfused with saline on a Langendorff system to wash blood from the coronary vasculature and then sliced horizontally. The slices were incubated in 1% TTC prepared with 200 mM Tris buffer (pH 7.8) for 15 min at 37 °C. (2) Trans-aortic constriction (TAC) (Liu et al, 2022). Briefly, 8–10-week-old male mice (25–27 g) were anesthetized, and the hearts were exposed through left thoracotomy in the third intercostal space. A 1.0 mm wire was placed alongside the transverse aorta, which was tied to the wire between the first and second branches of the aortic arch. The wire was quickly removed, leaving the aortic arch constricted to the diameter of the wire. For each type of surgical procedure, the mice were allowed to recover on a heating pad before being returned to the cage. Cardiac functions were evaluated by echocardiography (GE Vivid 7 equipped with a 14-MHz phase array linear transducer, S12, allowing a 150 maximal sweep rate). M-mode imaging was applied at the level of the papillary muscles to assess left ventricular dimensions. Representative echo images can be found in the Appendix Fig. S12. All data were obtained under steady-state conditions with minimal respiratory influence, and values were averaged over at least three consecutive cardiac cycles. Offline analysis was performed using VEVO Lab software (version 5.6.1). The animals were euthanized by carbon dioxide inhalation to obtain their samples.

### Cell culture, plasmids, and transient transfection

Primary murine cardiac fibroblasts were isolated and cultured as previously described (Khalil et al, 2017). Primary human cardiac fibroblasts were purchased from Lonza. Small interfering RNAs were purchased from Dharmacon. Transient transfections were performed with Lipofectamine 2000 or RNAiMax (Thermo Fisher).

### RNA Isolation and Real-time PCR

RNA was extracted with the RNeasy RNA isolation kit (Qiagen) as previously described (Kong et al, 2021a; Kong et al, 2021b). Reverse transcriptase reactions were performed using a SuperScript First-strand Synthesis System (Invitrogen). Real-time PCR reactions were performed on an ABI Prism 7500 system. The primers are listed in the Appendix Table S1. Ct values of target genes were normalized to the Ct values of the housekeeping control gene (18 s, 5’-CGCGGTTCTATTTTGTTGGT-3’ and 5’-TCGTCTTCGAAACTCCGACT-3’ for both human and mouse genes) using the ΔΔCt method and expressed as relative mRNA expression levels compared to the control group, which is arbitrarily set as 1.

### Protein extraction and western blotting

Whole-cell lysates were obtained by re-suspending cell pellets in RIPA buffer (50 mM Tris pH 7.4, 150 mM NaCl, 1% Triton X-100) with freshly added protease and phosphatase inhibitors (Roche) as previously described (Fan et al, 2021). Antibodies used for Western blotting are listed in the Appendix Table S2.

### Chromatin immunoprecipitation (ChIP)

Chromatin immunoprecipitation (ChIP) assays were performed essentially as described before (Li et al, 2023). In brief, chromatin in control and treated cells was cross-linked with 1% formaldehyde. Cells were incubated in lysis buffer (150 mM NaCl, 25 mM Tris pH 7.5, 1% Triton X-100, 0.1% SDS, 0.5% deoxycholate) supplemented with a protease inhibitor tablet. DNA was fragmented into ~200 bp pieces using a Branson 250 sonicator (Emerson, St. Louis, MO, USA). Aliquots of lysates containing 200 μg of protein were used for each immunoprecipitation reaction with 5 μg of anti-MKL1 (21166-1, Proteintech, Wuhan, China) or pre-immune IgG. Precipitated genomic DNA was amplified by real-time PCR. 10% of the starting material was included as the input. Data are expressed as relative enrichment (%) compared to input.

### PicroSirius red staining

PSR staining was performed using a commercially available kit (ab150681, Abcam, Cambridge, UK). Following dewaxing and rehydration, paraffin sections were immersed in PicroSirius Red staining solution and incubated for 1 h at room temperature under light-protected conditions. Subsequently, the sections were rinsed twice with 0.5% acetic acid solution to remove non-specific background staining. After conventional dehydration through a graded ethanol series and clearing, the sections were mounted with neutral gum. Following staining, images were captured using an optical microscope (DMI8, Leica, Wetzlar, Germany) at ×10 magnification, and whole-slide scans were obtained using a stereo microscope (M60, Leica, Wetzlar, Germany) for macroscopic observation.

### Masson’s trichrome staining

Masson’s trichrome staining was performed using a commercial kit (ab150686, Abcam, Cambridge, UK). Following dewaxing and rehydration, paraffin sections were stained sequentially as follows: acid fuchsin solution for 4 min, rinsed with 0.1% acetic acid for 1 min; differentiated with phosphomolybdate solution for 1–2 min, rinsed again with 0.1% acetic acid for 1 min; and counterstained with aniline blue solution for 4 min, followed by a final rinse with 0.1% acetic acid for 1 min. Sections were then dehydrated through a graded ethanol series to remove residual stain, cleared, and mounted with neutral gum. Following staining, images were captured using an optical microscope (DMI8, Leica, Wetzlar, Germany) at 10× magnification, and whole-slide scans were obtained using a stereo microscope (M60, Leica, Wetzlar, Germany) for macroscopic observation.

### Hydroxyproline quantification

Tissue hydroxyproline levels were quantified with a commercially available kit (ab222941, Abcam, Cambridge, UK) per vendor recommendation. Briefly, heart tissues (~10 mg) were homogenized with the BeadBlaster microtube homogenizer (D2400, LABRepCo, Horsham, PA, USA). The homogenates were then mixed with NaOH (10 N) at 120 °C for 1 h followed by neutralization with HCl (10 N). The samples were centrifuged at 10,000 × *g* for 5 min. The supernatants were collected and incubated with the Oxidation Reagent Mix in a 96-well microplate at room temperature for 20 min. Developer solution was added to and incubated with the samples 65 °C for 30 min followed by absorbance measurement at OD 560 nm on a SpectraMax ABS microplate reader (Molecular Devices, San Jose, CA, USA).

### Wheat germ agglutinin (WGA) staining

Paraffin sections were de-waxed, rehydrated, and then blocked with phosphate-buffered saline (PBS) containing 1% bovine serum albumin (BSA) for 30 min. The sections were then incubated with Alexa Fluor 488-conjugated WGA (W11261, Thermo Fisher) for 1 h at room temperature under light-protected conditions to label the cell membrane. After incubation, sections were washed three times with PBS to remove unbound dye. Finally, sections were mounted with an anti-fade mounting medium containing DAPI to counterstain nuclei. Images were captured using a fluorescence microscope (Leica, DMI8) equipped with a ×40 oil immersion objective. The cross-sectional area of cardiomyocytes with clearly defined borders was measured using ImageJ.

### EdU incorporation assay

5-ethynyl-2’-deoxyuridine (EdU) incorporation assay was performed in triplicate wells with a commercially available kit (Thermo Fisher) as previously described (Shao et al, 2021). Briefly, the EdU solution was diluted with the culture media and added to the cells for an incubation period of 2 h at 37 °C. After several washes with 1× PBS, the cells were then fixed with 4% formaldehyde and stained with Alexa Fluor™ 488. The nucleus was counterstained with DAPI. The images were visualized by fluorescence microscopy and analyzed with Image-Pro Plus (Media Cybernetics). For each well, six different fields were randomly chosen, and the positively stained cells were counted and divided by the total number of cells. The average of the six fields for each well was calculated. The averages of each group were then normalized to the averages of the control group. The data are expressed as relative EdU staining compared to the control group, arbitrarily set as 1. All experiments were performed in triplicate wells and repeated three times. One representative experiment is shown in the figures.

### Boyden chamber migration assay

The cells were trypsinized and seeded into Boyden chambers (PET track-etched, 8-μm pores, 24-well format; Becton Dickinson; cat#354597) in serum-free DMEM medium. Complete culture medium containing 10% FBS was added to the lower chamber. The cells migrating from the upper chamber were fixed with 4% paraformaldehyde, stained with 0.1% crystal violet, and counted under a microscope. Cell numbers from five random fields were counted in each well.

### Collagen contraction assay

The cells were trypsinized, mixed with 4× the volume of Collagen Gel Working Solution (Corning; cat#: 354236), and incubated for 1 h at 37 °C. After collagen polymerization, 1.0 mL of culture medium was added atop. The collagen gel size change was measured 24 h later and quantified with Image Pro Plus. Data are expressed as relative contraction normalized to the control group, arbitrarily set as 1.

### RNA sequencing and data analysis

RNA-seq was performed and analyzed as previously described (Wu et al, 2021). Total RNA was extracted using the TRIzol reagent according to the manufacturer’s protocol. RNA purity and quantification were evaluated using the NanoDrop 2000 spectrophotometer (Thermo Scientific, USA). RNA integrity was assessed using the Agilent 2100 Bioanalyzer (Agilent Technologies, Santa Clara, CA, USA). Then the libraries were constructed using TruSeq Stranded mRNA LT Sample Prep Kit (Illumina, San Diego, CA, USA) according to the manufacturer’s instructions and sequenced on an Illumina HiSeq X Ten platform, and 150 bp paired-end reads were generated. Raw data (raw reads) of fastq format were first processed using Trimmomatic, and the low-quality reads were removed to obtain the clean reads. The clean reads were mapped to the mouse genome (Mus_musculus.GRCm38.99) using HISAT2. FPKM of each gene was calculated using Cufflinks, and the read counts of each gene were obtained by HTSeqcount. Differential expression analysis was performed using the DESeq (2012) R package. *P* value < 0.05 and fold change >1.5 or fold change <0.66 were set as the threshold for significantly differential expression. Hierarchical cluster analysis of differentially expressed genes (DEGs) was performed to demonstrate the expression pattern of genes in different groups and samples. GO enrichment and KEGG pathway enrichment analysis of DEGs were performed, respectively, using R based on the hypergeometric distribution.

### CUT&Tag-sequencing and data processing

CUT&Tag assay was performed per vendor recommendations (Vazyme; cat#TD904). For each CUT&Tag experiment, 1 × 10^5^ cells were incubated with 10 µl of ConA Beads. Primary antibodies were then incubated overnight with the chromatin at 4 °C on a shaking platform. Beads-chromatin-antibody mixture was washed once with 200 µl of Dig-wash Buffer, resuspended in 50 µl of Dig-wash Buffer with a mouse secondary antibody, and incubated for 30 min at room temperature on a rotator. The mixture was washed twice with 200 µl of Dig-Wash Buffer and resuspended in 100 µl of Dig-300 Buffer containing 2 µl pA/G-Tnp Pro (protein A–Tn5 transposase fusion protein). After incubation with pA/G-Tnp Pro on a rotator at room temperature, the mixture was washed three times with 200 µl Dig-300 Buffer to remove unbound pA/G-Tnp Pro, and resuspended with 50 μl Tagmentation buffer. Subsequently, the reaction was stopped with 2 μl 10% SDS and 1 pg DNA Spike-in. DNA was extracted with phenol–chloroform and ethanol. Libraries were amplified according to the manufacturer’s instructions. Library quality was evaluated using agarose gel electrophoresis. The libraries were sequenced on an Illumina HiSeq 2500. Quality-filtered reads were mapped to the reference genome(GRCm39) using Bowite2. Masc2 was used to call peaks. Deep tools and Integrative Genomics Viewer (IGV) were used to accomplish data visualization.

### Human heart specimens

Patients with end-stage dilated cardiomyopathy or ischemic cardiomyopathy were evaluated and definitively diagnosed in accordance with the International Society for Heart and Lung Transplantation (ISHLT) guidelines and subsequently underwent heart transplantation at Tongji Hospital, Tongji Medical College, Huazhong University of Science and Technology. The samples were procured from the left ventricles of the hearts during the transplantation procedure. Prior to the collection of samples, written informed consent was obtained from all patients. The collection of samples was conducted in accordance with a human research protocol that had been approved by the Human Research Ethics Committees of Tongji Hospital, Tongji Medical College, Huazhong University of Science and Technology (TJ-IRB202412110) and conformed to the principles set out in the WMA Declaration of Helsinki and the Department of Health and Human Services Belmont Report. Patient information can be found in Appendix Table S3.

### Statistical analysis

For comparison between two groups, two-tailed *t* test was performed. For comparison among three or more groups, one-way ANOVA or two-way ANOVA with post hoc Turkey analyses were performed using an SPSS package. The assumptions of normality were checked using Shapiro–Wilks test, and equal variance was checked using Levene’s test; both were satisfied. *P* values smaller than .05 were considered statistically significant (*). All in vitro experiments were repeated at least three times, and three replicates were estimated to provide 80% power. Randomization and blinding were applied during sample allocation and data analysis. Inclusion criteria were defined prior to experimentation, and no data were excluded unless clear technical errors (e.g., equipment malfunction, failed assays or sample loss) occurred.

## Supplementary information

Appendix

Peer Review File

Source data Fig. 1

Source data Fig. 2

Source data Fig. 3

Source data Fig. 4

Source data Fig. 5

Source data Fig. 6

Source data Fig. 7

Source data Fig. 8

Appendix Figure Source Data Part I

Appendix Figure Source Data II

## Data Availability

RNA-seq data generated for this study have been deposited in the PubMed database with the accession number GSE317712. All the CUT&Tag-seq data generated for this study have been deposited in the PubMed database with the accession number GSE317630. The high-res images along with the metadata underlying Figs. 2G, 2R, 3J, 5G, 5R, and 7L have been deposited in BioStudies (https://www.ebi.ac.uk/biostudies/bioimages/studies/S-BIAD3242). The source data of this paper are collected in the following database record: biostudies:S-SCDT-10_1038-S44321-026-00444-2.

## References

[CR1] Allen-Gondringer A, Gau D, Varghese C, Boone D, Stolz D, Larregina A, Roy P (2023) Vascular endothelial cell-specific disruption of the profilin1 gene leads to severe multiorgan pathology and inflammation causing mortality. PNAS Nexus 2:pgad305 37781098 10.1093/pnasnexus/pgad305 PMC10541205

[CR2] Chen H, Chew G, Devapragash N, Loh JZ, Huang KY, Guo J, Liu S, Tan ELS, Chen S, Tee NGZ et al (2022a) The E3 ubiquitin ligase WWP2 regulates pro-fibrogenic monocyte infiltration and activity in heart fibrosis. Nat Commun 13:7375 36450710 10.1038/s41467-022-34971-6 PMC9712659

[CR3] Chen QT, Zhang ZY, Huang QL, Chen HZ, Hong WB, Lin T, Zhao WX, Wang XM, Ju CY, Wu LZ et al (2022b) HK1 from hepatic stellate cell-derived extracellular vesicles promotes progression of hepatocellular carcinoma. Nat Metab 4:1306–1321 36192599 10.1038/s42255-022-00642-5 PMC9584821

[CR4] Ding N, Yu RT, Subramaniam N, Sherman MH, Wilson C, Rao R, Leblanc M, Coulter S, He M, Scott C et al (2013) A vitamin D receptor/SMAD genomic circuit gates hepatic fibrotic response. Cell 153:601–613 23622244 10.1016/j.cell.2013.03.028 PMC3673534

[CR5] Duffy MJ, Synnott NC, O’Grady S, Crown J (2022) Targeting p53 for the treatment of cancer. Semin Cancer Biol 79:58–67 32741700 10.1016/j.semcancer.2020.07.005

[CR6] Fan Z, Kong M, Miao X, Guo Y, Ren H, Wang J, Wang S, Tang N, Shang L, Zhu Z et al (2021) An E2F5-TFDP1-BRG1 complex mediates transcriptional activation of MYCN in hepatocytes. Front Cell Dev Biol 9:742319 34746136 10.3389/fcell.2021.742319 PMC8569672

[CR7] Ibrahim MK, Salum GM, Bader El Din NG, Dawood RM, Barakat A, Khairy A, El Awady MK (2016) Transcriptional dysregulation of upstream signaling of IFN pathway in chronic HCV type 4 induced liver fibrosis. PLoS ONE 11:e0154512 27135246 10.1371/journal.pone.0154512 PMC4852926

[CR8] Jiang DS, Liu Y, Zhou H, Zhang Y, Zhang XD, Zhang XF, Chen K, Gao L, Peng J, Gong H et al (2014) Interferon regulatory factor 7 functions as a novel negative regulator of pathological cardiac hypertrophy. Hypertension 63:713–722 24396025 10.1161/HYPERTENSIONAHA.113.02653 PMC5349187

[CR9] Kanisicak O, Khalil H, Ivey MJ, Karch J, Maliken BD, Correll RN, Brody MJ, Lin SCJ, Aronow BJ, Tallquist MD et al (2016) Genetic lineage tracing defines myofibroblast origin and function in the injured heart. Nat Commun 7:12260 27447449 10.1038/ncomms12260 PMC5512625

[CR10] Katanasaka Y, Yabe H, Murata N, Sobukawa M, Sugiyama Y, Sato H, Honda H, Sunagawa Y, Funamoto M, Shimizu S et al (2024) Fibroblast-specific PRMT5 deficiency suppresses cardiac fibrosis and left ventricular dysfunction in male mice. Nat Commun 15:2472 38503742 10.1038/s41467-024-46711-z PMC10951424

[CR11] Kaur H, Takefuji M, Ngai CY, Carvalho J, Bayer J, Wietelmann A, Poetsch A, Hoelper S, Conway SJ, Mollmann H et al (2016) Targeted ablation of periostin-expressing activated fibroblasts prevents adverse cardiac remodeling in mice. Circ Res 118:1906–1917 27140435 10.1161/CIRCRESAHA.116.308643

[CR12] Khalil H, Kanisicak O, Prasad V, Correll RN, Fu X, Schips T, Vagnozzi RJ, Liu R, Huynh T, Lee SJ et al (2017) Fibroblast-specific TGF-beta-Smad2/3 signaling underlies cardiac fibrosis. J Clin Investig 127:3770–3783 28891814 10.1172/JCI94753 PMC5617658

[CR13] Khan MS, Shahid I, Bennis A, Rakisheva A, Metra M, Butler J (2024) Global epidemiology of heart failure. Nat Rev Cardiol 21:717–734 38926611 10.1038/s41569-024-01046-6

[CR14] Kong M, Dong W, Xu H, Fan Z, Miao X, Guo Y, Li C, Ye Q, Wang Y, Xu Y (2021a) Choline kinase alpha is a novel transcriptional target of the Brg1 in hepatocyte: implication in liver regeneration. Front Cell Dev Biol 9:705302 34422825 10.3389/fcell.2021.705302 PMC8377418

[CR15] Kong M, Dong W, Zhu Y, Fan Z, Miao X, Guo Y, Li C, Duan Y, Lu Y, Li Z et al (2021b) Redox-sensitive activation of CCL7 by BRG1 in hepatocytes during liver injury. Redox Biol 46:102079 34454163 10.1016/j.redox.2021.102079 PMC8406035

[CR16] Koop AC, Bossers GPL, Ploegstra MJ, Hagdorn QAJ, Berger RMF, Sillje HHW, Bartelds B (2019) Metabolic remodeling in the pressure-loaded right ventricle: shifts in glucose and fatty acid metabolism—a systematic review and meta-analysis. J Am Heart Assoc 8:e012086 31657265 10.1161/JAHA.119.012086 PMC6898858

[CR17] Li H, Bian Y (2024) Fibroblast-derived interleukin-6 exacerbates adverse cardiac remodeling after myocardial infarction. Korean J Physiol Pharmacol 28:285–294 38682176 10.4196/kjpp.2024.28.3.285 PMC11058547

[CR18] Li N, Liu H, Xue Y, Xu Z, Miao X, Guo Y, Li Z, Fan Z, Xu Y (2023) Targetable Brg1-CXCL14 axis contributes to alcoholic liver injury by driving neutrophil trafficking. EMBO Mol Med 15:e16592 36722664 10.15252/emmm.202216592 PMC9994483

[CR19] Liu L, Zhao Q, Kong M, Mao L, Yang Y, Xu Y (2022) Myocardin-related transcription factor A regulates integrin beta 2 transcription to promote macrophage infiltration and cardiac hypertrophy in mice. Cardiovasc Res 118:844–858 33752236 10.1093/cvr/cvab110

[CR20] Lopez B, Ravassa S, Moreno MU, Jose GS, Beaumont J, Gonzalez A, Diez J (2021) Diffuse myocardial fibrosis: mechanisms, diagnosis and therapeutic approaches. Nat Rev Cardiol 18:479–498 33568808 10.1038/s41569-020-00504-1

[CR21] Martin SS, Aday AW, Almarzooq ZI, Anderson CAM, Arora P, Avery CL, Baker-Smith CM, Barone Gibbs B, Beaton AZ, Boehme AK et al (2024) 2024 Heart disease and stroke statistics: a report of US and global data from the American Heart Association. Circulation 149:e347–e913 38264914 10.1161/CIR.0000000000001209 PMC12146881

[CR22] Patrick R, Janbandhu V, Tallapragada V, Tan SSM, McKinna EE, Contreras O, Ghazanfar S, Humphreys DT, Murray NJ, Tran YTH et al (2024) Integration mapping of cardiac fibroblast single-cell transcriptomes elucidates cellular principles of fibrosis in diverse pathologies. Sci Adv 10:eadk8501 38905342 10.1126/sciadv.adk8501 PMC11192082

[CR23] Rashid MM, Hamano M, Iida M, Iwata M, Ko T, Nomura S, Komuro I, Yamanishi Y (2024) Network-based identification of diagnosis-specific trans-omic biomarkers via integration of multiple omics data. Bio Syst 236:105122 10.1016/j.biosystems.2024.105122 38199520

[CR24] Rho H, Terry AR, Chronis C, Hay N (2023) Hexokinase 2-mediated gene expression via histone lactylation is required for hepatic stellate cell activation and liver fibrosis. Cell Metab 35:1406–1423.e1408 37463576 10.1016/j.cmet.2023.06.013 PMC11748916

[CR25] Rickman DS, Schulte JH, Eilers M (2018) The expanding world of N-MYC-driven tumors. Cancer Discov 8:150–163 29358508 10.1158/2159-8290.CD-17-0273

[CR26] Sermasathanasawadi R, Kato N, Muroyama R, Dharel N, Shao RX, Chang JH, Li CZ, Kawabe T, Omata M (2008) Association of interferon regulatory factor-7 gene polymorphism with liver cirrhosis in chronic hepatitis C patients. Liver Int 28:798–806 18397234 10.1111/j.1478-3231.2008.01725.x

[CR27] Shahbazian MD, Grunstein M (2007) Functions of site-specific histone acetylation and deacetylation. Annu Rev Biochem 76:75–100 17362198 10.1146/annurev.biochem.76.052705.162114

[CR28] Shao T, Xue Y, Fang M (2021) Epigenetic repression of chloride channel accessory 2 transcription in cardiac fibroblast: implication in cardiac fibrosis. Front Cell Dev Biol 9:771466 34869368 10.3389/fcell.2021.771466 PMC8633401

[CR29] Shi P, Ma Y, Zhang S (2025) Non-histone lactylation: unveiling its functional significance. Front Cell Dev Biol 13:1535611 39925738 10.3389/fcell.2025.1535611 PMC11802821

[CR30] Shilatifard A (2012) The COMPASS family of histone H3K4 methylases: mechanisms of regulation in development and disease pathogenesis. Annu Rev Biochem 81:65–95 22663077 10.1146/annurev-biochem-051710-134100 PMC4010150

[CR31] Sun H, Wang Y (2014) Interferon regulatory factors in heart: stress response beyond inflammation. Hypertension 63:663–664 24396026 10.1161/HYPERTENSIONAHA.113.02795 PMC4046326

[CR32] Tallquist MD, Molkentin JD (2017) Redefining the identity of cardiac fibroblasts. Nat Rev Cardiol 14:484–491 28436487 10.1038/nrcardio.2017.57 PMC6329009

[CR33] van den Borne SW, Diez J, Blankesteijn WM, Verjans J, Hofstra L, Narula J (2010) Myocardial remodeling after infarction: the role of myofibroblasts. Nat Rev Cardiol 7:30–37 19949426 10.1038/nrcardio.2009.199

[CR34] Wan Q, Kong D, Liu Q, Guo S, Wang C, Zhao Y, Ke ZJ, Yu Y (2021) Congestive heart failure in COX2 deficient rats. Sci China Life Sci 64:1068–1076 32955658 10.1007/s11427-020-1792-5

[CR35] Weisz A, Marx P, Sharf R, Appella E, Driggers PH, Ozato K, Levi BZ (1992) Human interferon consensus sequence binding protein is a negative regulator of enhancer elements common to interferon-inducible genes. J Biol Chem 267:25589–25596 1460054

[CR36] Wu M, Skaug B, Bi X, Mills T, Salazar G, Zhou X, Reveille J, Agarwal SK, Blackburn MR, Mayes MD et al (2019) Interferon regulatory factor 7 (IRF7) represents a link between inflammation and fibrosis in the pathogenesis of systemic sclerosis. Ann Rheum Dis 78:1583–1591 31439591 10.1136/annrheumdis-2019-215208 PMC7167109

[CR37] Wu X, Dong W, Kong M, Ren H, Wang J, Shang L, Zhu Z, Zhu W, Shi X (2021) Down-regulation of CXXC5 de-represses MYCL1 to promote hepatic stellate cell activation. Front Cell Dev Biol 9:680344 34621736 10.3389/fcell.2021.680344 PMC8490686

[CR38] Yang G, Weng X, Zhao Y, Zhang X, Hu Y, Dai X, Liang P, Wang P, Ma L, Sun X et al (2017) The histone H3K9 methyltransferase SUV39H links SIRT1 repression to myocardial infarction. Nat Commun 8:14941 28361889 10.1038/ncomms14941 PMC5381011

[CR39] Yu H, Zhu T, Ma D, Cheng X, Wang S, Yao Y (2024) The role of nonhistone lactylation in disease. Heliyon 10:e36296 39315193 10.1016/j.heliyon.2024.e36296 PMC11417196

[CR40] Zhang D, Tang Z, Huang H, Zhou G, Cui C, Weng Y, Liu W, Kim S, Lee S, Perez-Neut M et al (2019) Metabolic regulation of gene expression by histone lactylation. Nature 574:575–580 31645732 10.1038/s41586-019-1678-1 PMC6818755

[CR41] Zhang XJ, Jiang DS, Li H (2015) The interferon regulatory factors as novel potential targets in the treatment of cardiovascular diseases. Br J Pharmacol 172:5457–5476 25131895 10.1111/bph.12881 PMC4667854

