# Publisher Correction: IRF7 links HK1-dependent histone lactylation to fibroblast activation and cardiac fibrosis

**DOI:** 10.1038/s44321-026-00505-6

**Published:** 2026-09-11

**Authors:** Ming Kong, Chenghao Zhu, Yujia Xue, Wenxuan Hong, Guoqing Zhang, Dingsheng Jiang, Yong Xu, Junli Guo

**Affiliations:** 1 State Key Laboratory of Natural Medicines, Department of Pharmacology, China Pharmaceutical University, Nanjing, China; 2 Department of Cardiology, Affiliated Hospital of Jiangnan University, Wuxi, China; 3 Department of Geriatric Nephrology, Jiangsu Province People’s Hospital, the First Affiliated Hospital of Nanjing Medical University, Nanjing, China; 4 Division of Cardiovascular Surgery, Tongji Hospital, Tongji Medical College, Huazhong University of Science and Technology, Wuhan, China; 5 Institute of Biomedical Research, College of Agriculture and Biology, Liaocheng University, Liaocheng, China; 6 Hainan Provincial Key Laboratory for Tropical Cardiovascular Diseases Research and Key Laboratory of Emergency and Trauma of Ministry of Education, Institute of Cardiovascular Research, Department of Cardiology, the First Affiliated Hospital, Hainan Medical University, Haikou, China

**Correction to:** *EMBO Molecular Medicine* (2026) 18:2599–2616. https://doi.org/10.1038/s44321-026-00444-2 | Published online 21 May 2026

Figure 5 was inadvertently replaced with a duplicate of Figure 2. The correct version of Figure 5 is now shown. This error has been corrected in the HTML and PDF versions of the article.

