# Supplementary material for: IRF7 links HK1-dependent histone lactylation to fibroblast activation and cardiac fibrosis
Source: EMBO Mol Med. 2026 May 21;18(7):2599–616. doi: 10.1038/s44321-026-00444-2 (PMC13365489; doi:10.1038/s44321-026-00444-2)
Supplement: Supplementary file 1 — Appendix [file 44321_2026_444_MOESM1_ESM.pdf]

Kong M et al: Interferon regulatory factor 7 links HK1-dependent histone lactylation to fibroblast activation and cardiac fibrosis

Table of Contents for Appendix

Appendix Figure S1……………………………………………..Page 2
Appendix Figure S2……………………………………………..Page 3
Appendix Figure S3……………………………………………..Page 4
Appendix Figure S4……………………………………………..Page 5
Appendix Figure S5……………………………………………..Page 6
Appendix Figure S6……………………………………………..Page 7
Appendix Figure S7……………………………………………..Page 8
Appendix Figure S8……………………………………………..Page 9
Appendix Figure S9……………………………………………..Page 10
Appendix Figure S10……………………………………………..Page 11
Appendix Figure S11……………………………………………..Page 12
Appendix Figure S12……………………………………………..Page 13
Appendix Table S1………………………………………………..Page 14
Appendix Table S2………………………………………………..Page 15
Appendix Table S3………………………………………………..Page 16
Appendix Table S4………………………………………………..Page 17-20

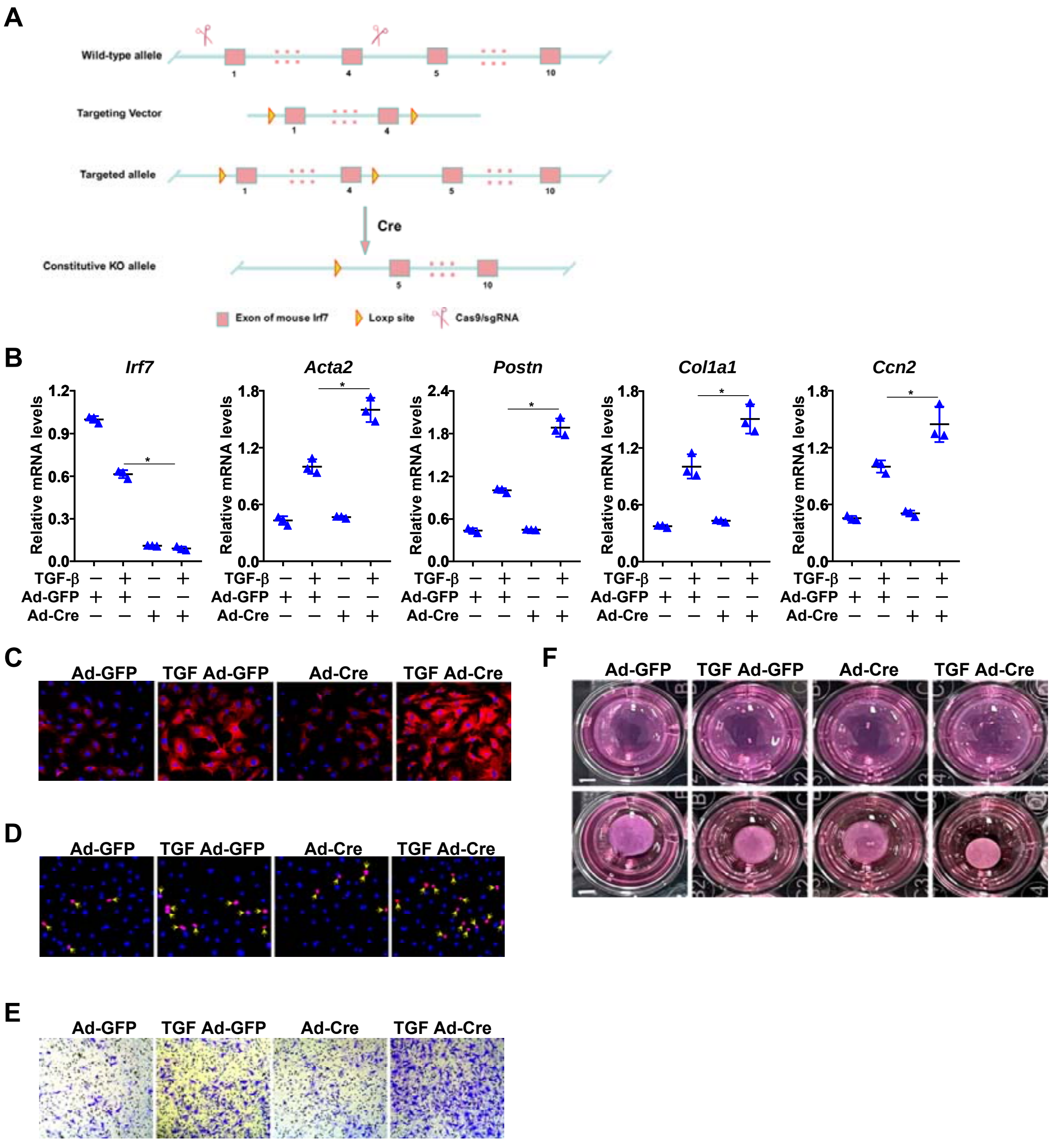

Appendix Figure S1: (**A-F**) Primary cardiac fibroblasts isolated from IRF7$^{f/f}$ mice were transduced with Ad-Cre or Ad-GFP followed by treatment with TGF-β (5ng/ml) for 24h. Targeting scheme of the IRF7$^{f/f}$ mice (A). Myofibroblast markers were examined by qPCR (B). Immunofluorescence staining with an anti-α-SMA antibody (C). EdU incorporation (D). Transwell assay (E). Collagen contraction assay (F). N=3 biological replicates. Data are expressed as mean±S.D. *, $p < 0.05$, one-way ANOVA with post-hoc Scheffe's test.

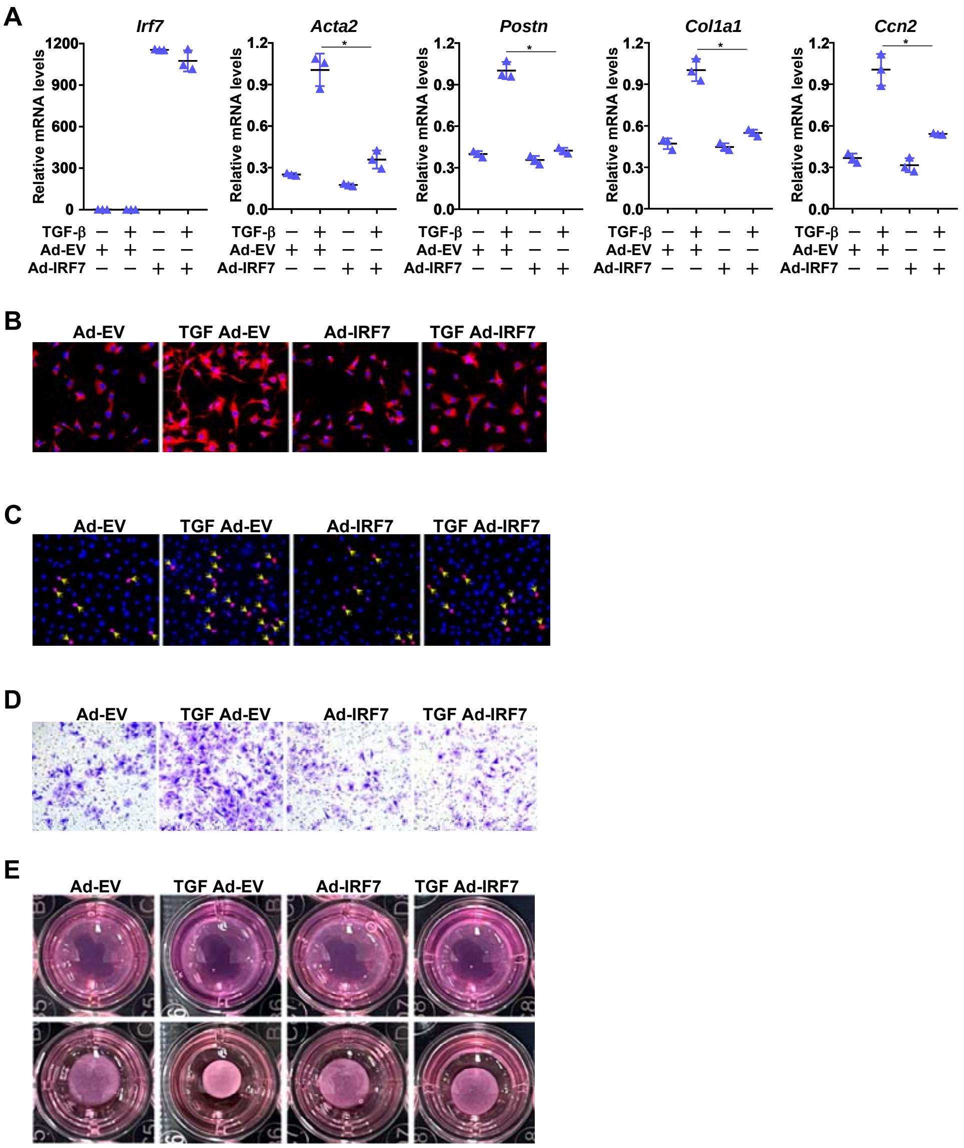

Appendix Figure S2: (**A-F**) Primary murine cardiac fibroblasts were transduced with indicated adenovirus followed by by treatment with TGF-β (5ng/ml) for 24h. Myofibroblast markers were examined by qPCR (A) and immunofluorescence staining (B). EdU incorporation assay (C). Transwell assay (D). Collagen contraction assay (E). N=3 biological replicates. Data are expressed as mean±S.D. *, $p < 0.05$, one-way ANOVA with post-hoc Scheffe's test.

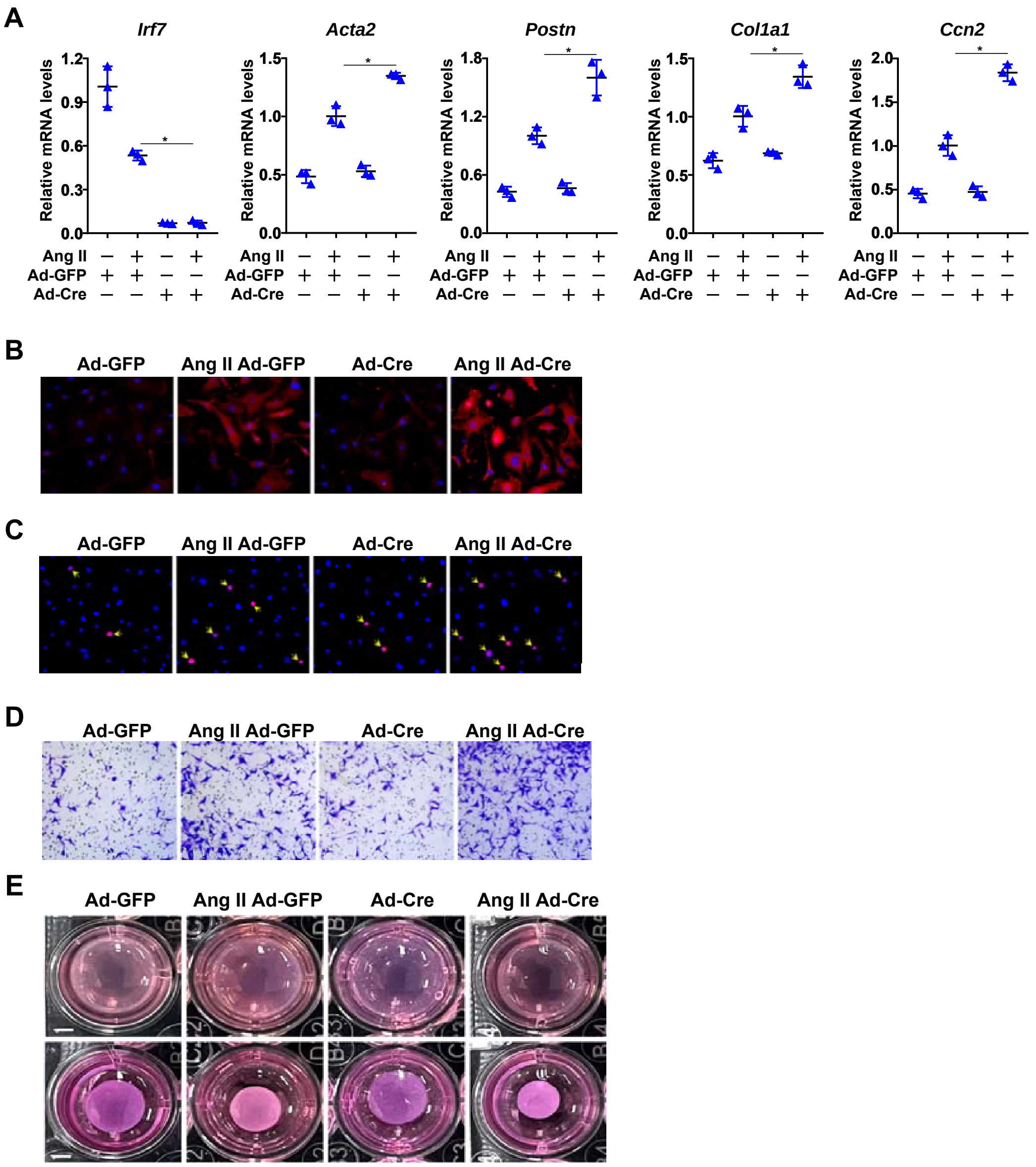

Appendix Figure S3: (**A-E**) Primary cardiac fibroblasts isolated from $IRF7^{f/f}$ mice were transduced with Ad-Cre or Ad-GFP followed by treatment with Ang II (1μM) for 24h. Myofibroblast markers were examined by qPCR (A). Immunofluorescence staining with an anti-α-SMA antibody (B). EdU incorporation (C). Transwell assay (D). Collagen contraction assay (E). N=3 biological replicates. Data are expressed as mean±S.D. *, $p<0.05$, one-way ANOVA with post-hoc Scheffe's test.

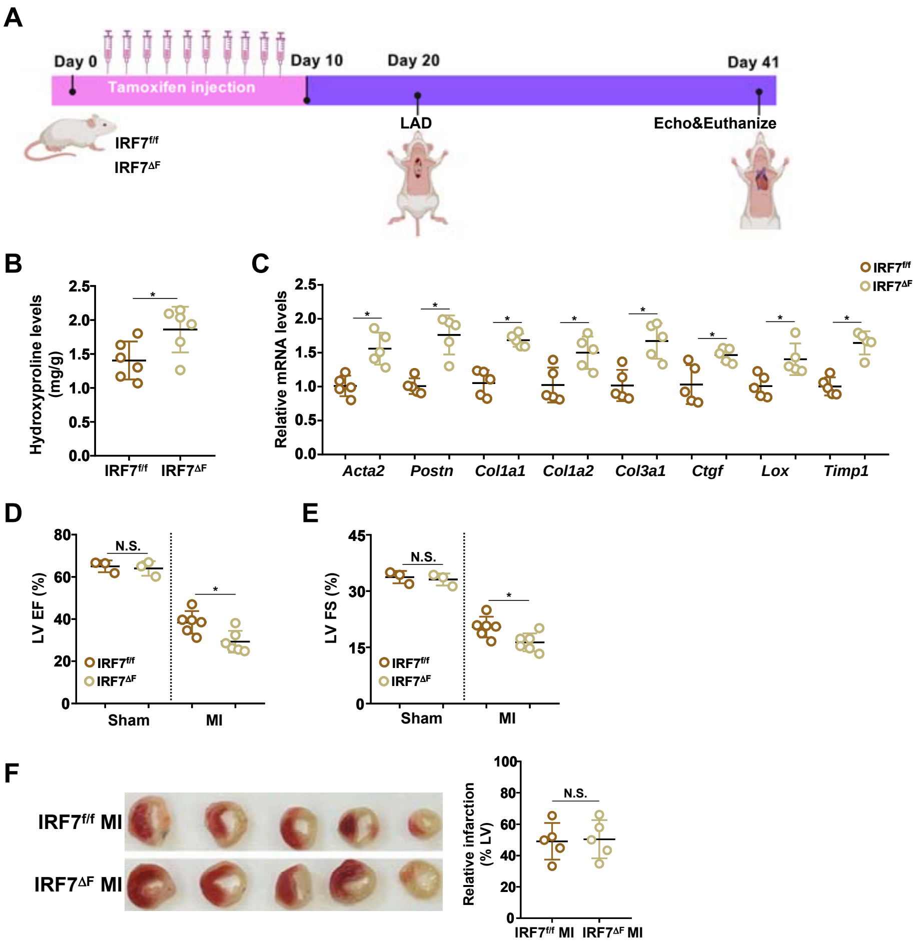

Appendix Figure S4: Myocardial infarction was induced in IRF7$^{f/f}$ mice and IRF7$^{\Delta F}$ mice by permanent ligation of the left anterior descending coronary artery. The mice were sacrificed 21 days after the surgery. (**A**) Scheme of protocol. (**B**) Hydroxyproline levels. (**C**) Myofibroblast markers were examined by qPCR. (**D**) LV EF. (**E**) LV FS. (**F**) Representative images of heart slices stained with TTC. N=3-6 mice for each group. Data are expressed as mean±S.D. *, $p < 0.05$, two-tailed student's test.

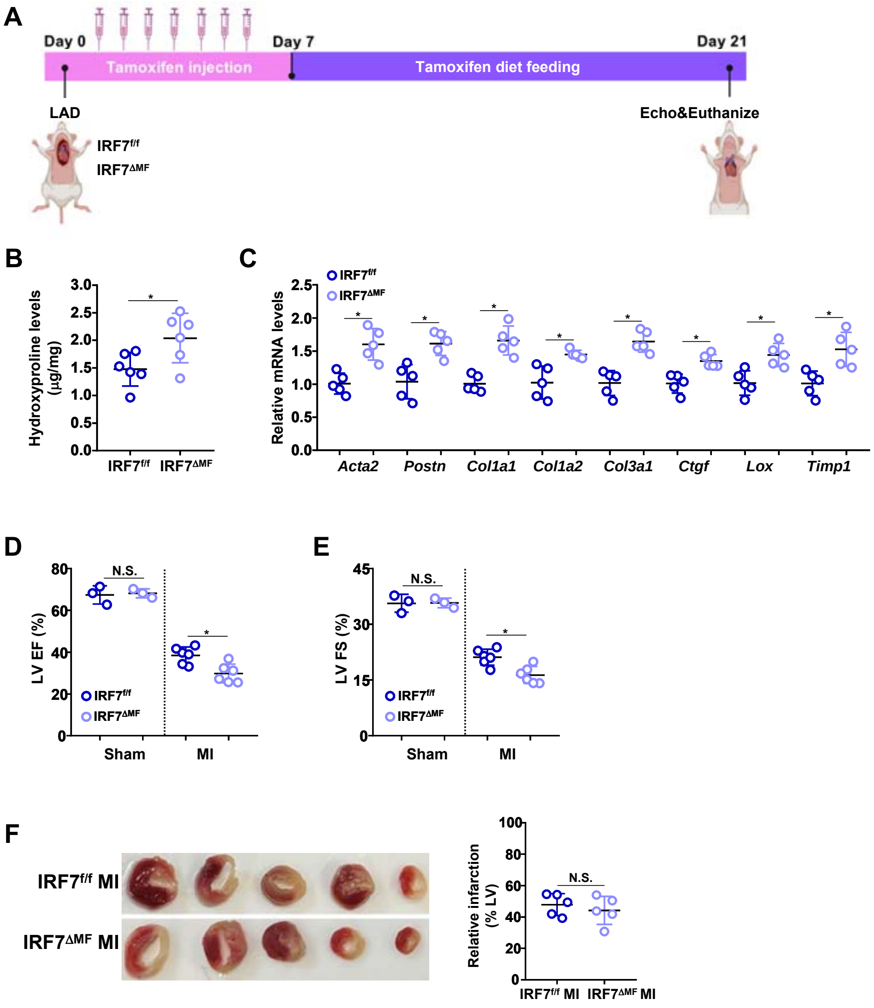

Appendix Figure S5: Myocardial infarction was induced in IRF7$^{f/f}$ mice and IRF7$^{\Delta MF}$ mice by permanent ligation of the left anterior descending coronary artery. The mice were sacrificed 21 days after the surgery. (**A**) Scheme of protocol. (**B**) Hydroxyproline levels. (**C**) Myofibroblast markers were examined by qPCR. (**D**) LV EF. (**E**) LV FS. (**F**) Representative images of heart slices stained with TTC. N=3-6 mice for each group. Data are expressed as mean±S.D. *, $p < 0.05$, two-tailed student's test.

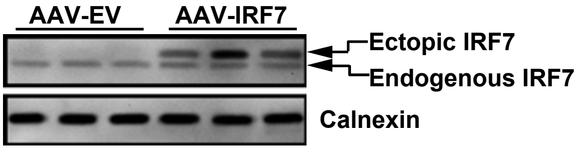

Appendix Figure S6: C57/B6j mice were subjected to the TAC procedure followed by tail vein injection of AAV9 carrying an IRF7 vector (AAV-IRF7) or an empty vector (AAV-EV). IRF7 expression in heart tissues was examined by Western blotting.

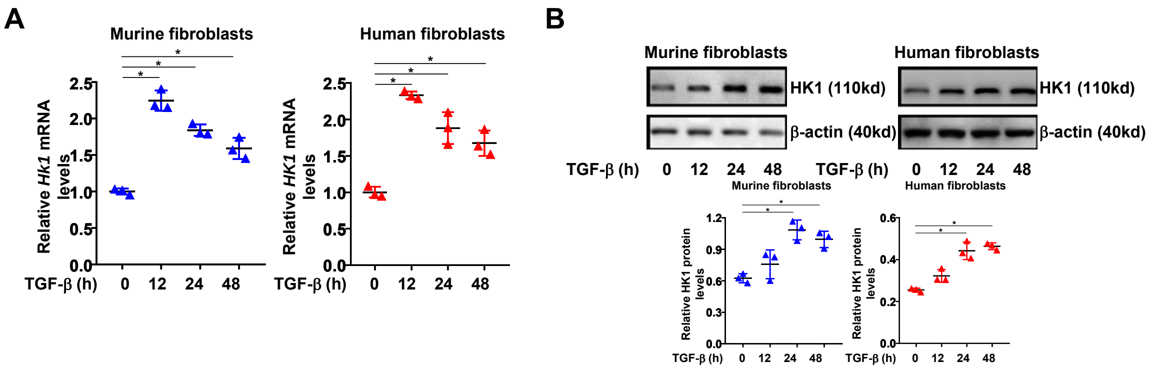

Appendix Figure S7: (**A, B**) Murine and human primary cardiac fibroblasts were treated with TGF-β (5ng/ml) and harvested at different time points. HK1 expression was examined by qPCR and Western blotting. N=3 biological replicates. Data are expressed as mean±S.D. *, $p < 0.05$, one-way ANOVA with post-hoc Scheffe's test.

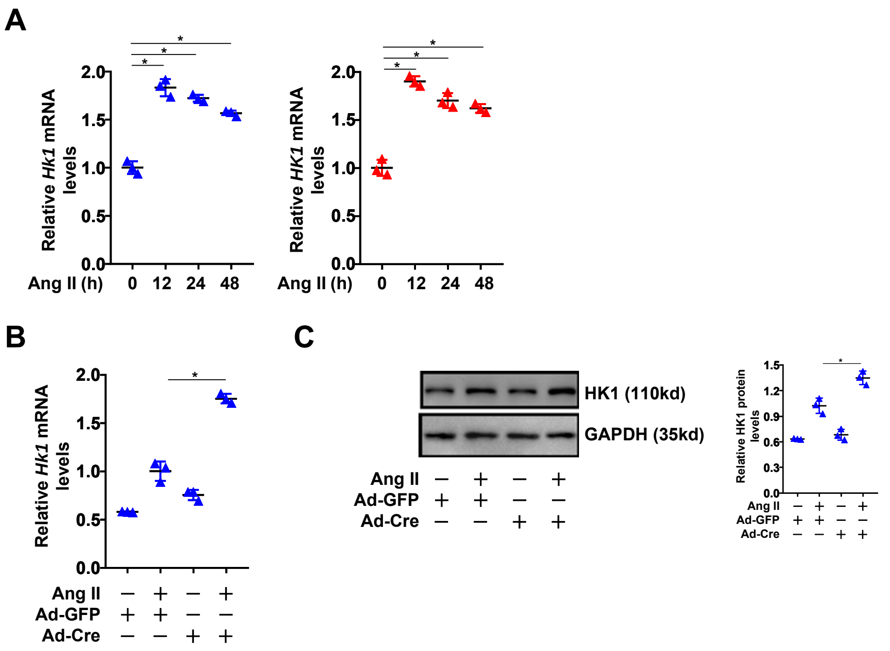

Appendix Figure S8: (**A**) Murine and human primary cardiac fibroblasts were treated with Ang II (1μM) and harvested at different time points. HK1 expression was examined by qPCR. (**B, C**) Primary cardiac fibroblasts isolated from IRF7$^{f/f}$ mice were transduced with Ad-Cre or Ad-GFP followed by treatment with Ang II (1μM) for 24h. HK1 expression was examined by qPCR and Western blotting. N=3 biological replicates. Data are expressed as mean±S.D. *, $p < 0.05$, one-way ANOVA with post-hoc Scheffe's test.

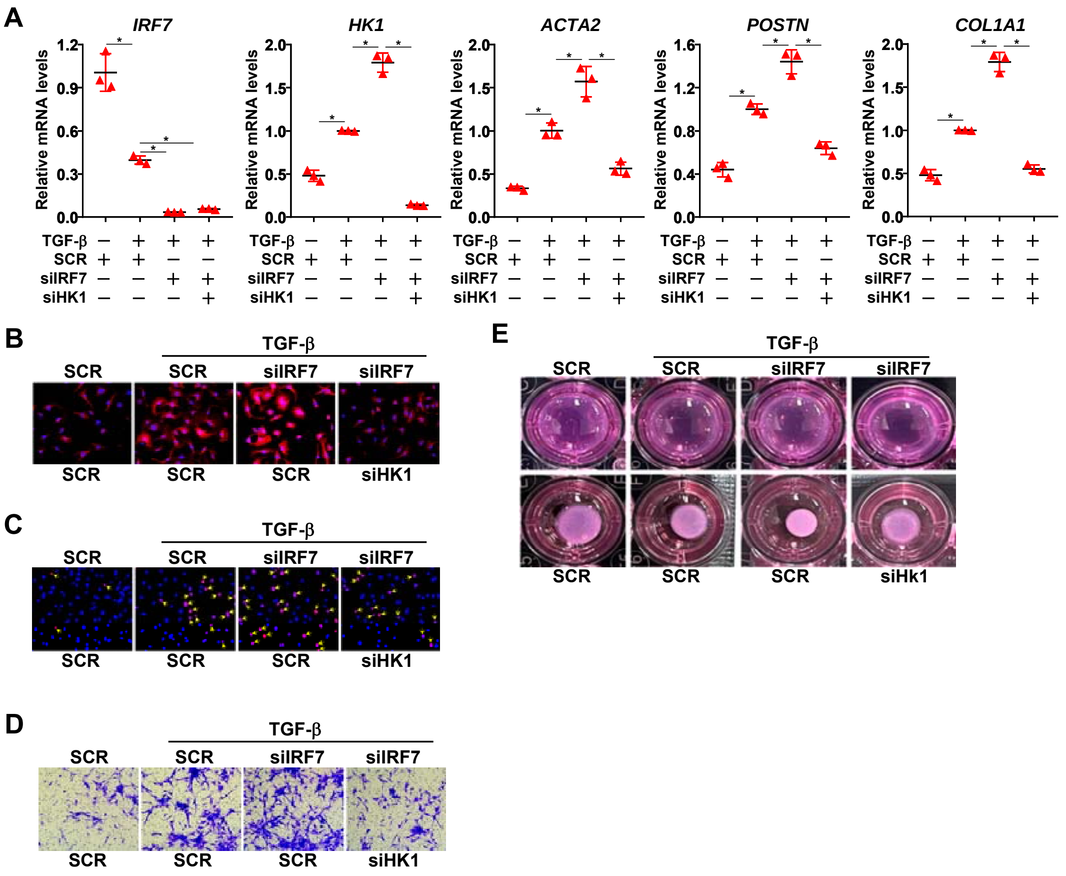

Appendix Figure S9: (**A-E**) Primary human cardiac fibroblasts were transfected with indicated siRNAs followed by treatment with TGF-β (5ng/ml) for 24h. Myofibroblast markers were examined by qPCR (A) and immunofluorescence staining (B). EdU incorporation assay (C). Transwell assay (D). Collagen contraction assay (E). N=3 biological replicates. Data are expressed as mean±S.D. *, $p < 0.05$, one-way ANOVA with post-hoc Scheffe's test.

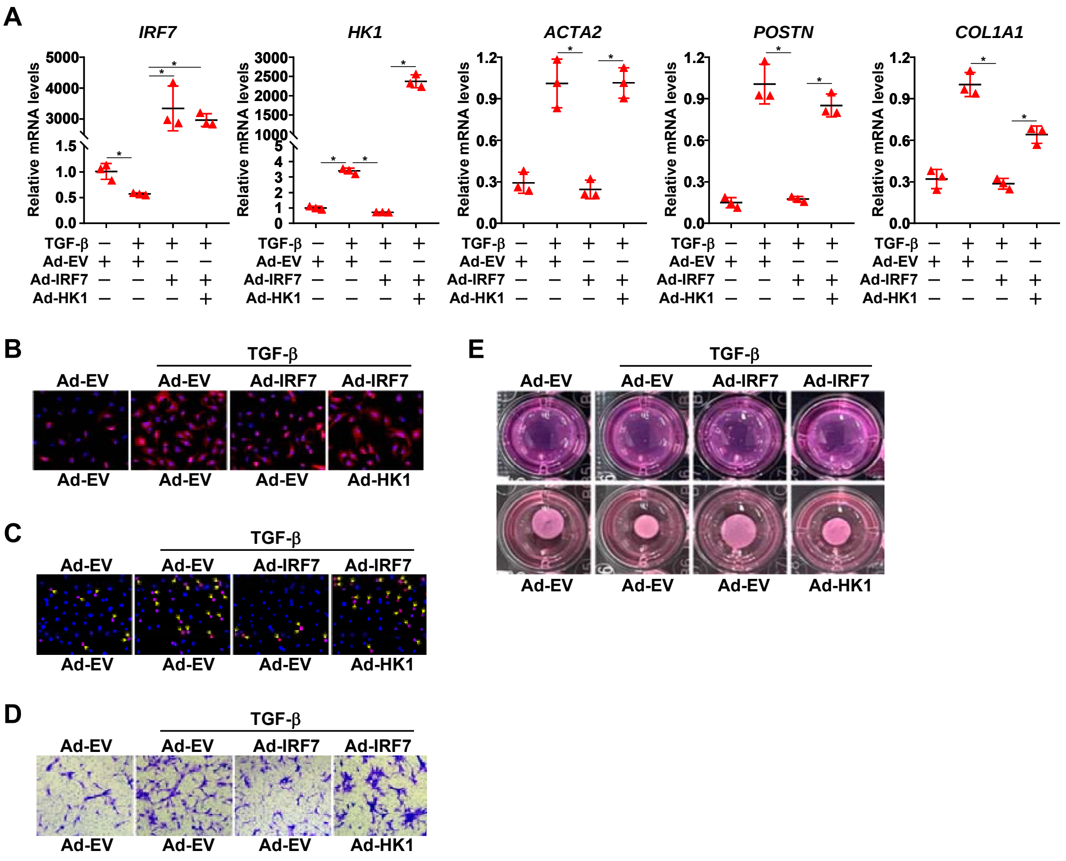

Appendix Figure S10: (**A-E**) Primary human cardiac fibroblasts were transduced with indicated adenovirus followed by treatment with TGF-β (5ng/ml) for 24h. Myofibroblast markers were examined by qPCR (A) and immunofluorescence staining (B). EdU incorporation assay (C). Transwell assay (D). Collagen contraction assay (E). N=3 biological replicates. Data are expressed as mean±S.D. *, $p<0.05$, one-way ANOVA with post-hoc Scheffe's test.

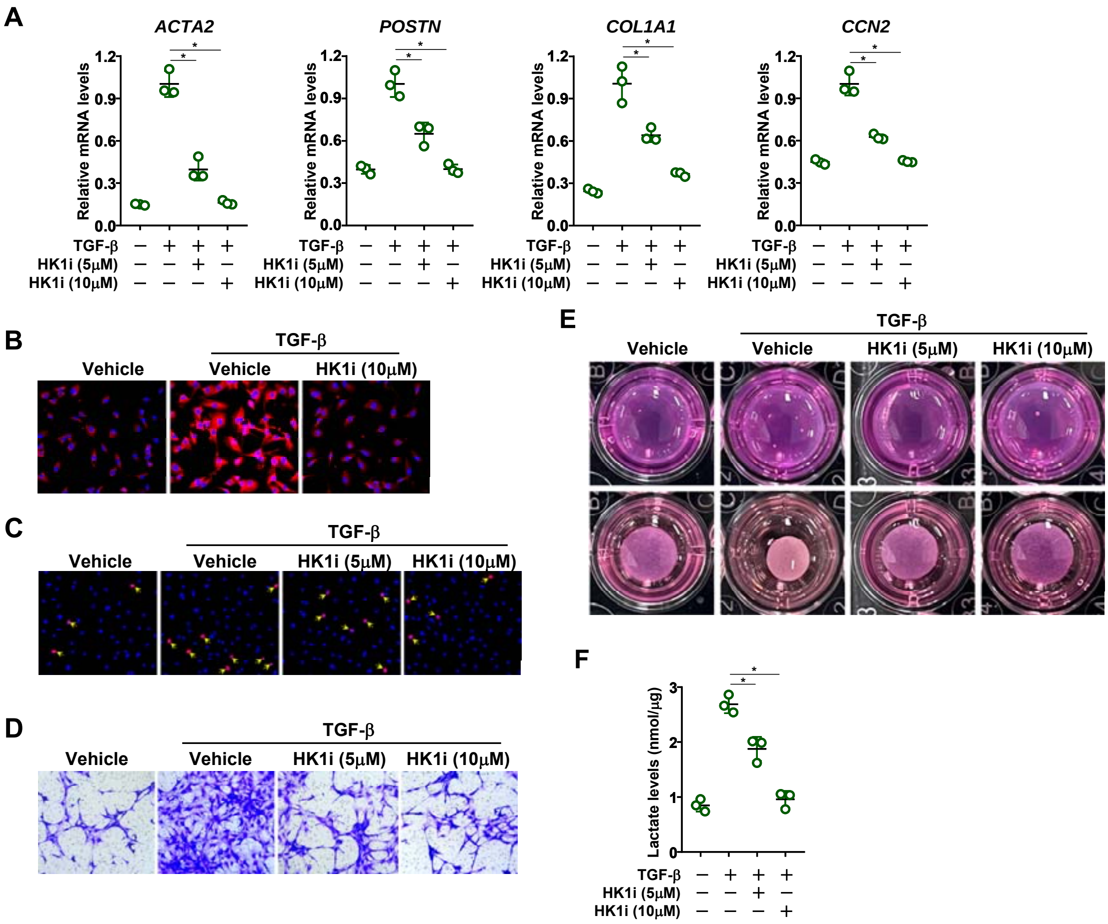

Appendix Figure S11: (**A-F**) Primary human cardiac fibroblasts were treated with TGF-β (5ng/ml) in the presence or absence of an HK1 inhibitor (HK1i) for 24h. Myofibroblast markers were examined by qPCR (A) and immunofluorescence staining (B). EdU incorporation assay (C). Transwell assay (D). Collagen contraction assay (E). Intracellular Lactate levels (F). N=3 biological replicates. Data are expressed as mean±S.D. *, $p < 0.05$, one-way ANOVA with post-hoc Scheffe's test.

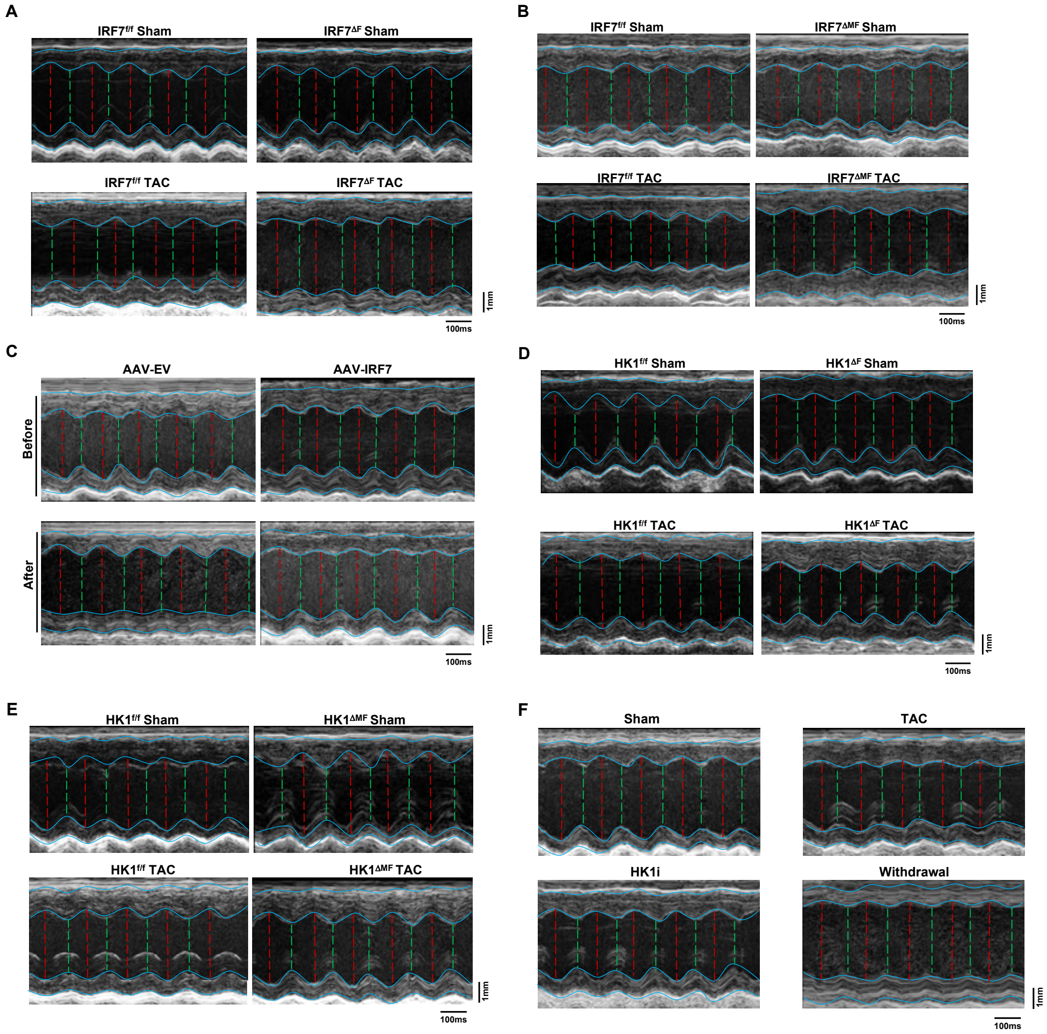

Appendix Figure S12: (**A**) IRF7$^{f/f}$ mice and IRF7$^{\Delta F}$ mice were subjected to the TAC procedure to induce heart failure. Shown here are representative M-mode echocardiography images of the indicated groups. (**B**) IRF7$^{f/f}$ mice and IRF7$^{\Delta MF}$ mice were subjected to the TAC procedure to induce heart failure. Shown here are representative M-mode echocardiography images of the indicated groups. (**C**) C57/B6j mice were subjected to the TAC procedure followed by tail vein injection of AAV9 carrying an IRF7 vector (AAV-IRF7) or an empty vector (AAV-EV). Shown here are representative M-mode echocardiography images of the indicated groups. (**D**) HK1$^{f/f}$ mice and HK1$^{\Delta F}$ mice were subjected to the TAC procedure to induce heart failure. Shown here are representative M-mode echocardiography images of the indicated groups. (**E**) HK1$^{f/f}$ mice and HK1$^{\Delta MF}$ mice were subjected to the TAC procedure to induce heart failure. Shown here are representative M-mode echocardiography images of the indicated groups. (**F**) C57/B6j mice were subjected to the TAC procedure followed by administration with HK1i. Shown here are representative M-mode echocardiography images of the indicated groups.

Appendix Table S1: QPCR Primers Sequences

Gene	Forward primer	Reverse primer
Mouse *Col1a1*	GCTCCTCTTAGGGGCCACT	ATTGGGGACCCTTAGGCCAT
Mouse *Col3a1*	CTGTAACATGGAAACTGGGGAAA	CCATAGCTGAACTGAAAACCACC
Mouse *Acta2*	CCCAGACATCAGGGAGTAATGG	TCTATCGGATACTTCAGCGTCA
Mouse *Col1a2*	TCGTGCCTAGCAACATGCC	TTTGTCAGAATACTGAGCAGCAA
Mouse *Ctgf*	GGCCTCTTCTGCGATTTCG	GCAGCTTGACCCTTCTCGG
Mouse *Postn*	CCTGCCCTTATATGCTCTGCT	AAACATGGTCAATAGGCATCACT
Mouse *Lox*	CAGCCACATAGATCGCATGGT	GCCGTATCCAGGTCGGTTC
Mouse *Irf7*	TCCAGTTGATCCGCATAAGGT	CTTCCCTATTTTCCGTGGCTG
Mouse *Hk1*	AACGGCCTCCGTCAAGATG	GCCGAGATCCAGTGCAATG
Mouse *Timp1*	CGAGACCACCTTATACCAGCG	ATGACTGGGGTGTAGGCGTA
Human *COL1A1*	GAGGGCCAAGACGAAGACATC	CAGATCACGTCATCGCACAAC
Human *COL3A1*	GGAGCTGGCTACTTCTCGC	GGGAACATCCTCCTTCAACAG
Human *ACTA2*	AAAAGACAGCTACGTGGGTGA	GCCATGTTCTATCGGGTACTTC
Human *POSTN*	CTCATAGTCGTATCAGGGGTCG	ACACAGTCGTTTTCTGTCCAC
Human *IRF7*	GCTGGACGTGACCATCATGTA	GGGCCGTATAGGAACGTGC
Human *TIMP1*	CTTCTGCAATTCCGACCTCGT	ACGCTGGTATAAGGTGGTCTG
Human *HK1*	GCTCTCCGATGAAACTCTCATAG	GGACCTTACGAATGTTGGCAA

Appendix Table S2: Antibody Information

Antigen	Vendor (catalog#)	Application
β-actin	Sigma (A2228)	IB (1:5000)
HK1	Proteintech (19662-1)	IB (1: 1000)
c-JUN	Abcam (Ab32137)	CUT&Tag-seq (1:100)
SMAD3	Abcam (Ab208182)	CUT&Tag-seq (1:100)
NFATC2	Novus Biologicals (NB300-504)	CUT&Tag-seq (1:100)
H3K9Lc	PTM-Biolabs (PTM-1419RM)	IB (1:1000), CUT&Tag-seq (1:100)
H3K12Lc	PTM-Biolabs (PTM-1411RM)	IB (1:1000)
H3K14Lc	PTM-Biolabs (PTM-1414RM)	IB (1:1000)
H3K18Lc	PTM-Biolabs (PTM-1406RM)	IB (1:1000)
α-SMA	Abcam (ab150301)	IF (1:100)

Appendix Table S3: Patient information

ID	Gender (M/F)	Age (Yr)	cTnT (ng/L)	Total cholesterol (mM)	LV EF (%)
1	M	56	19	4.48	25
2	M	54	270	4.41	36
3	F	68	16	6.38	31
4	M	59	71	3.19	11
5	F	8	22	3.39	40
6	M	51	29	3.29	37
7	F	67	47	3.20	26
8	M	51	48	1.68	26
9	M	51	51	2.15	39

Appendix Table S4: Exact p values

Figure #	Statistical method	Groups	Exact *p* value
Figure 1A IRF7 mRNA	One way ANOVA	SCR TGF vs siIRF7#1 TGF	2.20E-05
	One way ANOVA	SCR TGF vs siIRF7#2 TGF	2.20E-05
Figure 1A ACTA2 mRNA	One way ANOVA	SCR TGF vs siIRF7#1 TGF	0.01
	One way ANOVA	SCR TGF vs siIRF7#2 TGF	0.001
Figure 1A POSTN mRNA	One way ANOVA	SCR TGF vs siIRF7#1 TGF	0.0015
	One way ANOVA	SCR TGF vs siIRF7#2 TGF	1.50E-05
Figure 1A COL1A1 mRNA	One way ANOVA	SCR TGF vs siIRF7#1 TGF	0.0085
	One way ANOVA	SCR TGF vs siIRF7#2 TGF	0.017
Figure 1A COL3A1 mRNA	One way ANOVA	SCR TGF vs siIRF7#1 TGF	0.0021
	One way ANOVA	SCR TGF vs siIRF7#2 TGF	3.70E-05
Figure 1A CCN2 mRNA	One way ANOVA	SCR TGF vs siIRF7#1 TGF	3.10E-04
	One way ANOVA	SCR TGF vs siIRF7#2 TGF	8.20E-05
Figure 1F ACTA2 mRNA	One way ANOVA	Ad-GFP TGF vs Ad-IRF7 TGF	0.0007
Figure 1F POSTN mRNA	One way ANOVA	Ad-GFP TGF vs Ad-IRF7 TGF	7.60E-05
Figure 1F COL1A1 mRNA	One way ANOVA	Ad-GFP TGF vs Ad-IRF7 TGF	0.00032
Figure 1F CCN2 mRNA	One way ANOVA	Ad-GFP TGF vs Ad-IRF7 TGF	0.00089
Figure 2G PSR staining	two-tailed student’s test	CKO TAC vs WT TAC	0.0031
Figure 2G Masson's staining	two-tailed student’s test	CKO TAC vs WT TAC	0.04
Figure 2H hydroxyproline	two-tailed student’s test	CKO TAC vs WT TAC	2.20E-05
Figure 2I Acta2 mRNA	two-tailed student’s test	CKO TAC vs WT TAC	0.00032
Figure 2I Postn mRNA	two-tailed student’s test	CKO TAC vs WT TAC	3.50E-06
Figure 2I Col1a1 mRNA	two-tailed student’s test	CKO TAC vs WT TAC	0.0013
Figure 2I Col1a2 mRNA	two-tailed student’s test	CKO TAC vs WT TAC	0.0011
Figure 2I Col3a1 mRNA	two-tailed student’s test	CKO TAC vs WT TAC	0.0026
Figure 2I Ctgf mRNA	two-tailed student’s test	CKO TAC vs WT TAC	0.001
Figure 2I Lox mRNA	two-tailed student’s test	CKO TAC vs WT TAC	2.70E-05
Figure 2I Timp1 RNA	two-tailed student’s test	CKO TAC vs WT TAC	0.00015
Figure 2J	two-tailed student’s test	CKO TAC vs WT TAC	0.0056
Figure 2K	two-tailed student’s test	CKO TAC vs WT TAC	0.006
Figure 2R PSR staining	two-tailed student’s test	CKO TAC vs WT TAC	0.037
Figure 2R Masson's staining	two-tailed student’s test	CKO TAC vs WT TAC	0.019
Figure 2S hydroxyproline	two-tailed student’s test	CKO TAC vs WT TAC	0.00021
Figure 2T Acta2 mRNA	two-tailed student’s test	CKO TAC vs WT TAC	0.002
Figure 2T Postn mRNA	two-tailed student’s test	CKO TAC vs WT TAC	8.90E-06
Figure 2T Col1a1 mRNA	two-tailed student’s test	CKO TAC vs WT TAC	0.00043
Figure 2T Col1a2 mRNA	two-tailed student’s test	CKO TAC vs WT TAC	0.00068
Figure 2T Col3a1 mRNA	two-tailed student’s test	CKO TAC vs WT TAC	0.00058
Figure 2T Ctgf mRNA	two-tailed student’s test	CKO TAC vs WT TAC	0.001
Figure 2T Lox mRNA	two-tailed student’s test	CKO TAC vs WT TAC	0.001
Figure 2T Timp1 RNA	two-tailed student’s test	CKO TAC vs WT TAC	0.0021
Figure 2U	two-tailed student’s test	CKO TAC vs WT TAC	0.001
Figure 2V	two-tailed student’s test	CKO TAC vs WT TAC	0.001
Figure 3B	two-tailed student’s test	AAV-IRF7 vs AAV-EV	0.016
Figure 3J PSR staining	two-tailed student’s test	AAV-IRF7 vs AAV-EV	0.0031
Figure 3J Masson's staining	two-tailed student’s test	AAV-IRF7 vs AAV-EV	0.0044
Figure 3K hydroxyproline	two-tailed student’s test	AAV-IRF7 vs AAV-EV	6.40E-05
Figure 3L Acta2 mRNA	two-tailed student’s test	AAV-IRF7 vs AAV-EV	2.90E-05
Figure 3L Postn mRNA	two-tailed student’s test	AAV-IRF7 vs AAV-EV	0.00026
Figure 3L Col1a1 mRNA	two-tailed student’s test	AAV-IRF7 vs AAV-EV	1.00E-05
Figure 3L Col1a2 mRNA	two-tailed student’s test	AAV-IRF7 vs AAV-EV	0.0022
Figure 3L Col3a1 mRNA	two-tailed student’s test	AAV-IRF7 vs AAV-EV	1.50E-05
Figure 3L Lox mRNA	two-tailed student’s test	AAV-IRF7 vs AAV-EV	0.0029
Figure 3L Timp1 RNA	two-tailed student’s test	AAV-IRF7 vs AAV-EV	0.00041
Figure 3M	two-tailed student’s test	AAV-IRF7 vs AAV-EV	0.00023
Figure 3N	two-tailed student’s test	AAV-IRF7 vs AAV-EV	0.0002
Figure 4J human HK1 mRNA	One way ANOVA	Ad-GFP TGF vs Ad-IRF7 TGF	0.00024
Figure 4J mouse Hk1 mRNA	One way ANOVA	Ad-GFP TGF vs Ad-IRF7 TGF	0.00016
Figure 4K human HK1 protein	One way ANOVA	Ad-GFP TGF vs Ad-IRF7 TGF	0.0036
Figure 4K mouse HK1 protein	One way ANOVA	Ad-GFP TGF vs Ad-IRF7 TGF	0.023
Figure 4L human HK1 mRNA	One way ANOVA	SCR TGF vs siIRF7#1 TGF	0.00088
	One way ANOVA	SCR TGF vs siIRF7#2 TGF	0.0025
Figure 4L mouse Hk1 mRNA	One way ANOVA	Ad-GFP TGF vs Ad-Cre TGF	0.0016
Figure 4M human HK1 protein	One way ANOVA	SCR TGF vs siIRF7#1 TGF	0.0043
	One way ANOVA	SCR TGF vs siIRF7#2 TGF	0.016
Figure 4M mouse HK1 protein	One way ANOVA	Ad-GFP TGF vs Ad-Cre TGF	0.048
Figure 4N	One way ANOVA	wild type IRF7 low vs empty vector	0.0028
	One way ANOVA	wild type IRF7 mid vs empty vector	0.00022
	One way ANOVA	wild type IRF7 high vs empty vector	5.20E-05
Figure 4O	two-tailed student’s test	minus TGF vs plus TGF	0.002

Figure 5G PSR staining	two-tailed student's test	CKO TAC vs WT TAC	0.031
Figure 5G Masson's staining	two-tailed student's test	CKO TAC vs WT TAC	0.0024
Figure 5H hydroxyproline	two-tailed student's test	CKO TAC vs WT TAC	1.50E-03
Figure 5I Acta2 mRNA	two-tailed student's test	CKO TAC vs WT TAC	0.008
Figure 5I Postn mRNA	two-tailed student's test	CKO TAC vs WT TAC	0.00089
Figure 5I Col1a1 mRNA	two-tailed student's test	CKO TAC vs WT TAC	0.00047
Figure 5I Col1a2 mRNA	two-tailed student's test	CKO TAC vs WT TAC	0.00023
Figure 5I Col3a1 mRNA	two-tailed student's test	CKO TAC vs WT TAC	3.50E-05
Figure 5I Ctgf mRNA	two-tailed student's test	CKO TAC vs WT TAC	0.00057
Figure 5I Lox mRNA	two-tailed student's test	CKO TAC vs WT TAC	0.0052
Figure 5I Timp1 RNA	two-tailed student's test	CKO TAC vs WT TAC	0.00022
Figure 5J	two-tailed student's test	CKO TAC vs WT TAC	0.00097
Figure 5K	two-tailed student's test	CKO TAC vs WT TAC	0.00083
Figure 5R PSR staining	two-tailed student's test	CKO TAC vs WT TAC	0.00059
Figure 5R Masson's staining	two-tailed student's test	CKO TAC vs WT TAC	0.0006
Figure 5S hydroxyproline	two-tailed student's test	CKO TAC vs WT TAC	0.0064
Figure 5T Acta2 mRNA	two-tailed student's test	CKO TAC vs WT TAC	0.004
Figure 5T Postn mRNA	two-tailed student's test	CKO TAC vs WT TAC	0.0014
Figure 5T Col1a1 mRNA	two-tailed student's test	CKO TAC vs WT TAC	0.00054
Figure 5T Col1a2 mRNA	two-tailed student's test	CKO TAC vs WT TAC	0.0029
Figure 5T Col3a1 mRNA	two-tailed student's test	CKO TAC vs WT TAC	0.0016
Figure 5T Ctgf mRNA	two-tailed student's test	CKO TAC vs WT TAC	0.0064
Figure 5T Lox mRNA	two-tailed student's test	CKO TAC vs WT TAC	0.003
Figure 5T Timp1 RNA	two-tailed student's test	CKO TAC vs WT TAC	0.003
Figure 5U	two-tailed student's test	CKO TAC vs WT TAC	0.00059
Figure 5V	two-tailed student's test	CKO TAC vs WT TAC	0.00034
Figure 6A human lactate	One way ANOVA	Ad-EV minus TGF vs plus TGF	1.50E-05
	One way ANOVA	Ad-EV TGF vs Ad-IRF7 TGF	1.70E-05
	One way ANOVA	Ad-IRF7 TGF vs Ad-HK1 Ad-IRF7 TGF	4.40E-05
Figure 6A mouse lactate	One way ANOVA	Ad-EV minus TGF vs plus TGF	0.00031
	One way ANOVA	Ad-EV TGF vs Ad-IRF7 TGF	0.00014
	One way ANOVA	Ad-IRF7 TGF vs Ad-HK1 Ad-IRF7 TGF	8.10E-06
Figure 6B human lactate	One way ANOVA	SCR minus TGF vs plus TGF	0.00027
	One way ANOVA	SCR TGF vs siIRF7 TGF	0.00025
	One way ANOVA	siIRF7 TGF vs siIRF7 siHK1 TGF	0.00085
Figure 6B mouse lactate	One way ANOVA	Ad-GFP minus vs plus TGF	0.00048
	One way ANOVA	Ad-GFP TGF vs Ad-Cre TGF	0.00016
	One way ANOVA	Ad-Cre TGF vs Ad-Cre siHK1 TGF	0.00016
Figure 6C H3K9Lc protein	One way ANOVA	SCR minus TGF vs plus TGF	0.0032
	One way ANOVA	SCR TGF vs siHK1 TGF	0.0067
Figure 6C H3K18Lc protein	One way ANOVA	SCR minus TGF vs plus TGF	0.004
	One way ANOVA	SCR TGF vs siHK1 TGF	0.039
Figure 6C H3K9Ac protein	One way ANOVA	SCR minus TGF vs plus TGF	0.0041
	One way ANOVA	SCR minus TGF vs siHK1 TGF	0.008
Figure 6C H3K4Me3 protein	One way ANOVA	SCR minus TGF vs plus TGF	0.0041
	One way ANOVA	SCR minus TGF vs siHK1 TGF	0.0043
Figure 6C HK1 protein	One way ANOVA	SCR minus TGF vs plus TGF	0.0026
	One way ANOVA	SCR TGF vs siHK1 TGF	0.0007
Figure 7A Acta2 mRNA	One way ANOVA	HK1i low TGF vs TGF	0.017
	One way ANOVA	HK1i mid TGF vs TGF	0.0019
	One way ANOVA	HK1i high TGF vs TGF	0.00055
Figure 7A Postn mRNA	One way ANOVA	HK1i low TGF vs TGF	0.022
	One way ANOVA	HK1i mid TGF vs TGF	0.0031
	One way ANOVA	HK1i high TGF vs TGF	2.40E-03
Figure 7A Col1a1 mRNA	One way ANOVA	HK1i low TGF vs TGF	0.00071
	One way ANOVA	HK1i mid TGF vs TGF	0.00014
	One way ANOVA	HK1i high TGF vs TGF	1.60E-05
Figure 7A Ccn2 mRNA	One way ANOVA	HK1i low TGF vs TGF	0.00067
	One way ANOVA	HK1i mid TGF vs TGF	4.90E-05
	One way ANOVA	HK1i high TGF vs TGF	5.30E-07
Figure 7F	One way ANOVA	HK1i low TGF vs TGF	0.00018
	One way ANOVA	HK1i mid TGF vs TGF	4.50E-05
	One way ANOVA	HK1i high TGF vs TGF	7.80E-05
Figure 7H	One way ANOVA	TAC vs sham 3w	0.0062
	One way ANOVA	TAC HK1i vs sham 3w	7.60E-06
	One way ANOVA	TAC HK1i withdrawal vs sham 3w	1.50E-05
	One way ANOVA	TAC vs sham 6w	3.10E-05
	One way ANOVA	TAC HK1i vs sham 6w	2.00E-05
	One way ANOVA	TAC HK1i withdrawal vs sham 6w	0.00027
	One way ANOVA	TAC vs sham 8w	0.0019
	One way ANOVA	TAC HK1i vs sham 8w	0.00012
	One way ANOVA	TAC HK1i withdrawal vs sham 8w	1.10E-05

Figure 7I	One way ANOVA	TAC vs sham 3w	0.016
	One way ANOVA	TAC HK1i vs sham 3w	8.70E-05
	One way ANOVA	TAC HK1i withdrawal vs sham 3w	6.00E-05
	One way ANOVA	TAC vs sham 6w	5.00E-07
	One way ANOVA	TAC HK1i vs sham 6w	4.20E-05
	One way ANOVA	TAC HK1i withdrawal vs sham 6w	3.40E-06
	One way ANOVA	TAC vs sham 8w	8.10E-05
	One way ANOVA	TAC HK1i vs sham 8w	2.10E-05
	One way ANOVA	TAC HK1i withdrawal vs sham 8w	6.80E-05
Figure 7L PSR staining	One way ANOVA	TAC vs TAC HK1i	0.015
	One way ANOVA	TAC HK1i vs TAC HK1i withdrawal	0.0087
Figure 7L Masson's staining	One way ANOVA	TAC vs TAC HK1i	0.00073
	One way ANOVA	TAC HK1i vs TAC HK1i withdrawal	0.0036
Figure 7M	One way ANOVA	TAC vs TAC HK1i	0.013
	One way ANOVA	TAC HK1i vs TAC HK1i withdrawal	3.10E-05
Figure 7N Acta2 mRNA	One way ANOVA	TAC vs TAC HK1i	0.00067
	One way ANOVA	TAC HK1i vs TAC HK1i withdrawal	0.00018
Figure 7N Postn mRNA	One way ANOVA	TAC vs TAC HK1i	0.00017
	One way ANOVA	TAC HK1i vs TAC HK1i withdrawal	0.00099
Figure 7N Col1a1 mRNA	One way ANOVA	TAC vs TAC HK1i	0.00017
	One way ANOVA	TAC HK1i vs TAC HK1i withdrawal	0.0017
Figure 7N Col1a2 mRNA	One way ANOVA	TAC vs TAC HK1i	1.20E-05
	One way ANOVA	TAC HK1i vs TAC HK1i withdrawal	0.00013
Figure 7N Col3a1 mRNA	One way ANOVA	TAC vs TAC HK1i	1.20E-05
	One way ANOVA	TAC HK1i vs TAC HK1i withdrawal	0.00081
Figure 7N Ccn2 mRNA	One way ANOVA	TAC vs TAC HK1i	7.40E-05
	One way ANOVA	TAC HK1i vs TAC HK1i withdrawal	0.03
Figure 7O	One way ANOVA	TAC vs TAC HK1i	4.80E-06
	One way ANOVA	TAC HK1i vs TAC HK1i withdrawal	2.80E-06
Figure 7P	One way ANOVA	TAC vs TAC HK1i 6w	2.20E-06
	One way ANOVA	TAC HK1i vs TAC HK1i withdrawal	1.20E-07
Figure 7Q	One way ANOVA	TAC vs TAC HK1i	3.00E-06
	One way ANOVA	TAC HK1i vs TAC HK1i withdrawal	8.70E-07
Figure 8A COL1A1 mRNA	two-tailed student’s test	HF vs healthy	0.0049
Figure 8A IRF7 mRNA	two-tailed student’s test	HF vs healthy	0.000099
Figure 8A HK1 mRNA	two-tailed student’s test	HF vs healthy	0.00092
Figure 8B IRF7 protein	two-tailed student’s test	HF vs healthy	0.0002
Figure 8B HK1 protein	two-tailed student’s test	HF vs healthy	0.0016
Figure S1B Irf7 mRNA	One way ANOVA	Ad-GFP TGF vs Ad-Cre TGF	7.60E-06
Figure S1B Acta2 mRNA	One way ANOVA	Ad-GFP TGF vs Ad-Cre TGF	0.0023
Figure S1B Postn mRNA	One way ANOVA	Ad-GFP TGF vs Ad-Cre TGF	0.0003
Figure S1B Col1a1 mRNA	One way ANOVA	Ad-GFP TGF vs Ad-Cre TGF	0.012
Figure S1B Ccn2 mRNA	One way ANOVA	Ad-GFP TGF vs Ad-Cre TGF	0.017
Figure S2A Acta2 mRNA	One way ANOVA	Ad-GFP TGF vs Ad-IRF7 TGF	0.0011
Figure S2A Postn mRNA	One way ANOVA	Ad-GFP TGF vs Ad-IRF7 TGF	0.0001
Figure S2A Col1a1 mRNA	One way ANOVA	Ad-GFP TGF vs Ad-IRF7 TGF	0.00072
Figure S2A Ccn2 mRNA	One way ANOVA	Ad-GFP TGF vs Ad-IRF7 TGF	0.0022
Figure S3A Irf7 mRNA	One way ANOVA	Ad-GFP Ang II vs Ad-Cre Ang II	2.80E-05
Figure S3A Acta2 mRNA	One way ANOVA	Ad-GFP Ang II vs Ad-Cre Ang II	0.0026
Figure S3A Postn mRNA	One way ANOVA	Ad-GFP Ang II vs Ad-Cre Ang II	0.0069
Figure S3A Col1a1 mRNA	One way ANOVA	Ad-GFP Ang II vs Ad-Cre Ang II	0.011
Figure S3A Ccn2 mRNA	One way ANOVA	Ad-GFP Ang II vs Ad-Cre Ang II	0.00068
Figure S4B	two-tailed student’s test	WT MI vs CKO MI	0.028
Figure S4C Acta2 mRNA	two-tailed student’s test	WT MI vs CKO MI	0.0024
Figure S4C Postn mRNA	two-tailed student’s test	WT MI vs CKO MI	0.00062
Figure S4C Col1a1 mRNA	two-tailed student’s test	WT MI vs CKO MI	0.00017
Figure S4C Col1a2 mRNA	two-tailed student’s test	WT MI vs CKO MI	0.016
Figure S4C Col3a1 mRNA	two-tailed student’s test	WT MI vs CKO MI	0.003
Figure S4C Ctgf mRNA	two-tailed student’s test	WT MI vs CKO MI	0.013
Figure S4C Lox mRNA	two-tailed student’s test	WT MI vs CKO MI	0.015
Figure S4C Timp1 RNA	two-tailed student’s test	WT MI vs CKO MI	0.00017
Figure S4D	two-tailed student’s test	WT MI vs CKO MI	0.013
Figure S4E	two-tailed student’s test	WT MI vs CKO MI	0.022
Figure S5B	two-tailed student’s test	WT MI vs CKO MI	0.029
Figure S5C Acta2 mRNA	two-tailed student’s test	WT MI vs CKO MI	0.0016
Figure S5C Postn mRNA	two-tailed student’s test	WT MI vs CKO MI	0.0035
Figure S5C Col1a1 mRNA	two-tailed student’s test	WT MI vs CKO MI	0.00042
Figure S5C Col1a2 mRNA	two-tailed student’s test	WT MI vs CKO MI	0.0057
Figure S5C Col3a1 mRNA	two-tailed student’s test	WT MI vs CKO MI	0.00046
Figure S5C Ctgf mRNA	two-tailed student’s test	WT MI vs CKO MI	0.0024
Figure S5C Lox mRNA	two-tailed student’s test	WT MI vs CKO MI	0.0058
Figure S5C Timp1 RNA	two-tailed student’s test	WT MI vs CKO MI	0.0069
Figure S5D	two-tailed student’s test	WT MI vs CKO MI	0.0054
Figure S5E	two-tailed student’s test	WT MI vs CKO MI	0.0038

Figure S7A mouse Hk1 mRNA	One way ANOVA	12h vs 0h	0.00012
	One way ANOVA	24h vs 0h	8.30E-05
	One way ANOVA	48h vs 0h	0.0025
Figure S7A human HK1 mRNA	One way ANOVA	12h vs 0h	1.40E-05
	One way ANOVA	24h vs 0h	0.0027
	One way ANOVA	48h vs 0h	0.0035
Figure S7B mouse HK1 protein	One way ANOVA	24h vs 0h	0.0017
	One way ANOVA	48h vs 0h	4.00E-05
Figure S7B human HK1 protein	One way ANOVA	24h vs 0h	0.0016
	One way ANOVA	48h vs 0h	0.002
Figure S8A mouse Hk1 mRNA	One way ANOVA	12h vs 0h	0.00021
	One way ANOVA	24h vs 0h	7.80E-05
	One way ANOVA	48h vs 0h	0.00017
Figure S8A human HK1 mRNA	One way ANOVA	12h vs 0h	9.40E-05
	One way ANOVA	24h vs 0h	0.00045
	One way ANOVA	48h vs 0h	0.00035
Figure S8B	One way ANOVA	Ad-GFP Ang II vs Ad-Cre Ang II	0.00031
Figure S8C	One way ANOVA	Ad-GFP Ang II vs Ad-Cre Ang II	0.0093
Figure S9A IRF7 mRNA	One way ANOVA	SCR minus TGF vs plus TGF	0.0014
	One way ANOVA	TGF SCR vs TGF siIRF7	2.40E-05
	One way ANOVA	TGF SCR vs TGF siIRF7 siHK1	3.30E-05
Figure S9A HK1 mRNA	One way ANOVA	SCR minus TGF vs plus TGF	0.00015
	One way ANOVA	TGF SCR vs TGF siIRF7	0.00026
	One way ANOVA	TGF siIRF7 vs TGF siIRF7 siHK1	1.40E-05
Figure S9A ACTA2 mRNA	One way ANOVA	SCR minus TGF vs plus TGF	0.00022
	One way ANOVA	TGF SCR vs TGF siIRF7	0.0075
	One way ANOVA	TGF siIRF7 vs TGF siIRF7 siHK1	0.00079
Figure S9A POSTN mRNA	One way ANOVA	SCR minus TGF vs plus TGF	0.00032
	One way ANOVA	TGF SCR vs TGF siIRF7	0.0033
	One way ANOVA	TGF siIRF7 vs TGF siIRF7 siHK1	0.00038
Figure S9A COL1A1 mRNA	One way ANOVA	SCR minus TGF vs plus TGF	7.20E-05
	One way ANOVA	TGF SCR vs TGF siIRF7	0.00017
	One way ANOVA	TGF siIRF7 vs TGF siIRF7 siHK1	4.10E-05
Figure S10A IRF7 mRNA	One way ANOVA	Ad-EV minus TGF vs plus TGF	0.025
	One way ANOVA	Ad-EV TGF vs Ad-IRF7 TGF	0.0013
	One way ANOVA	Ad-EV TGF vs Ad-HK1 Ad-IRF7 TGF	1.60E-05
Figure S10A HK1 mRNA	One way ANOVA	Ad-EV minus TGF vs plus TGF	8.10E-05
	One way ANOVA	Ad-EV TGF vs Ad-IRF7 TGF	1.00E-05
	One way ANOVA	Ad-IRF7 TGF vs Ad-HK1 Ad-IRF7 TGF	1.70E-05
Figure S10A ACTA2 mRNA	One way ANOVA	Ad-EV TGF vs Ad-IRF7 TGF	0.0021
	One way ANOVA	Ad-IRF7 TGF vs Ad-HK1 Ad-IRF7 TGF	0.00049
Figure S10A POSTN mRNA	One way ANOVA	Ad-EV TGF vs Ad-IRF7 TGF	0.00057
	One way ANOVA	Ad-IRF7 TGF vs Ad-HK1 Ad-IRF7 TGF	0.00016
Figure S10A COL1A1 mRNA	One way ANOVA	Ad-EV TGF vs Ad-IRF7 TGF	0.0002
	One way ANOVA	Ad-IRF7 TGF vs Ad-HK1 Ad-IRF7 TGF	0.0011
Figure S11A ACTA2 mRNA	One way ANOVA	HK1i low TGF vs TGF	0.00098
	One way ANOVA	HK1i high TGF vs TGF	9.70E-05
Figure S11A POSTN mRNA	One way ANOVA	HK1i low TGF vs TGF	0.0072
	One way ANOVA	HK1i high TGF vs TGF	0.00044
Figure S11A COL1A1 mRNA	One way ANOVA	HK1i low TGF vs TGF	0.01
	One way ANOVA	HK1i high TGF vs TGF	0.00011
Figure S11A CCN2	One way ANOVA	HK1i low TGF vs TGF	0.0014
	One way ANOVA	HK1i high TGF vs TGF	0.0003
Figure S11F	One way ANOVA	HK1i low TGF vs TGF	0.0068
	One way ANOVA	HK1i high TGF vs TGF	0.00018
