# Supplementary material for: IRF7 links HK1-dependent histone lactylation to fibroblast activation and cardiac fibrosis
Source: EMBO Mol Med. 2026 May 21;18(7):2599–616. doi: 10.1038/s44321-026-00444-2 (PMC13365489; doi:10.1038/s44321-026-00444-2)
Supplement: Supplementary file 2 — Peer Review File [file 44321_2026_444_MOESM2_ESM.pdf]

EMBO Molecular Medicine

IRF7 links HK1-dependent histone lactylation to fibroblast activation and cardiac fibrosis

Ming Kong, Chenghao Zhu, Yujia Xue, Wenxuan Hong, Guoqing Zhang, Ding-Sheng Jiang, Yong Xu, and Junli Guo

Corresponding author(s): Ming Kong (mingkong@cpu.edu.cn) , Yong Xu (yjxu@cpu.edu.cn), Junli Guo (hy0301002@muhn.edu.cn)

Review Timeline:

Submission Date:	24th Sep 25
Editorial Decision:	21st Oct 25
Revision Received:	28th Jan 26
Editorial Decision:	11th Feb 26
Revision Received:	2nd Mar 26
Editorial Decision:	25th Mar 26
Revision Received:	31st Mar 26
Editorial Decision:	9th Apr 26
Revision Received:	14th Apr 26
Accepted:	27th Apr 26

Editor: Lise Roth

Transaction Report:

(Note: With the exception of the correction of typographical or spelling errors that could be a source of ambiguity, letters and reports are not edited. Depending on transfer agreements, referee reports obtained elsewhere may or may not be included in this compilation. Referee reports are anonymous unless the Referee chooses to sign their reports.)

21st Oct 2025

Dear Dr. Kong,

Thank you for the submission of your manuscript to EMBO Molecular Medicine. We have now received feedback from the three reviewers who agreed to evaluate your manuscript.

As you will see from the enclosed reports, the reviewers agree that the findings are of potential interest, but they also raise major and partially overlapping concerns related to the weak mechanistic insight, the lack of adequate controls, and some experimental inconsistencies. Based on the nature of the concerns and the amount of time and work that would be needed to address them, considering also that at EMBO Press we encourage a single round of revisions in a limited time frame, we prefer to return the manuscript to you at this point with the decision that we cannot offer to publish it.

However, given the potential interest of the findings, we would welcome a new manuscript on the same topic if you obtained data that would address the referees' concerns in full.

To be completely clear, however, I would like to stress that if you were to send a new manuscript, this would be treated as a new submission rather than a revision and would be reviewed afresh, in particular with respect to the literature and the novelty of your findings at the time of resubmission. If you decide to follow this route, please make sure you nevertheless upload a letter of response to the referees' comments.

I am sorry that I could not bring better news this time and hope that the referee comments are helpful in your continued work in this area.

With kind regards,

Lise Roth

Lise Roth, Ph.D
Senior Editor
EMBO Molecular Medicine

***** Reviewer's comments *****

Referee #1 (Comments on Novelty/Model System for Author):

1. Lack of appropriate controls: In the in vivo models induced by surgical procedures, appropriate negative control groups were not included or presented in parallel. The same issue is observed in several in vitro experiments. All relevant control data should be provided throughout the figures.

Referee #1 (Remarks for Author):

Zhu et al. present a study proposing a novel role of IRF7 in cardiac fibrosis. In their manuscript entitled "IRF7 links HK1-dependent histone lactylation to fibroblast activation and cardiac fibrosis", the authors demonstrate that loss of IRF7 exacerbates fibrotic remodeling following TAC or LAD ligation, whereas IRF7 overexpression attenuates fibrosis under similar pathological stimuli. Genomic analyses suggest that IRF7 directly regulates HK1 transcription, thereby influencing intracellular lactate levels. Loss of IRF7 subsequently promotes histone lactylation, which in turn enhances fibrotic gene expression. While the study addresses an interesting mechanism linking metabolism and epigenetic regulation in cardiac fibrosis, several issues need to be carefully addressed before the manuscript can be further considered:

1. Lack of appropriate controls: In the in vivo models induced by surgical procedures, appropriate negative control groups were not included or presented in parallel. The same issue is observed in several in vitro experiments. All relevant control data should be provided throughout the figures.
2. Mechanistic interpretation of histone lactylation: The manuscript should discuss how histone H3 lysine lactylation modulates transcriptional activity. As lysine acetylation generally promotes transcription, the authors need to clarify whether lactylation exerts similar or distinct effects.
3. Ethical approval disclosure: The manuscript must include the approval numbers for both IACUC (animal study) and IRB (human sample) protocols.
4. Inconsistency in experimental schedules: The experimental timelines presented in Figures 2A and 2K, as well as Figures 5A

and 5K, differ from each other without clear explanation. The rationale for these discrepancies should be clarified.
5. Need for ChIP validation: The data shown in Figure 4N should be replaced or supplemented with ChIP results to confirm the direct binding of IRF7 to the HK1 promoter.
6. Inconsistent experimental comparisons: The rationale for certain comparisons, such as that shown in Figure 1A, is unclear. Specifically, why was TGF-β treatment applied only to the siIRF7#2 condition? Similar inconsistencies are noted in other figures and should be justified.
7. Missing loading control: In Figure 6C, total histone H3 should be included as a loading control for the histone lactylation analysis.

Referee #2 (Comments on Novelty/Model System for Author):

This study used different cardiac heart failure models including TAC and myocardial infarction with target genes knockout and overexpression, demonstrated the regulatory effects of IRF7 and its downstream HK1 in cardiac fibroblasts and fibrosis. The amount of data and the results are impressive. Moreover, an inhibitor of HK1's effect was tested and also IRF7 and HK1's expression were examined in clinical samples, highlights the translational potential of the results.

Referee #2 (Remarks for Author):

This study examined the role of IRF7 and its downstream target HK1 in cardiac fibrosis. Two cardiac heart failure models including TAC and myocardial infarction with target genes knockout and overexpression were used. The amount of data and the results are impressive. Moreover, an inhibitor of HK1's effect was tested and also IRF7 and HK1's expression were examined in clinical samples, highlights the translational potential of the results. Despite these, there are several concerns need to be addressed:
1. The major concern of the study is that the link between IRF7 and HK1 is weak. Although there are data showing that IFR7 knockout or overexpression leads to changes of HK1 in their models. However, how IFR7 regulates HK1 in cardiac fibroblasts is still not clear. Whether that is through promoting HK1 transcription (and by how?) or other mechanism, is unclear. Also, whether manipulation of HK1 will affect IFR7's effects on fibrosis is also not clear.
2. The knockout mice were generated using either Col1a2 or postn cre mice, a discussion is needed to point out this is not deletion of IRF7 or HK1 in all fibroblasts but activated fibroblasts (vs. TCF21-Cre)
3. It looks like the tamoxifen system is used differently in different model, is there any specific reason for this? Fig.1A vs Fig.K.
4. For several experiments presented, such as Fig.1F-H, Fig2 E-K, control groups are missing. E.g., does IFR7 knockdown or overexpression affect fibrosis gene expression?
5. Please provide representative images of echocardiography.
6. For AAV overexpression, please provide protein expression data to see how much the target was increased.
7. For the MI model, please provide data for the changes of infraction and also presentative images for the histological staining of the hearts.

Referee #3 (Comments on Novelty/Model System for Author):

Although genomic analyses are quantitatively performed, protein level analyses are not quantitatively analysed. Need substantial analyses.

Referee #3 (Remarks for Author):

This study is intersting. One serious concenrn is that quantitative statistical analyses at the protein level (i.e., immunohistochemistry, picrosirus red staining and western blotting bands) are generally lacking. Also, immunoblot bands should be published not only as cropped images but also as images of the whole membranes.

As a service to authors, EMBO provides authors with the possibility to transfer a manuscript that one journal cannot offer to publish to another EMBO publication. The full manuscript and if applicable, reviewers reports are automatically sent to the receiving journal to allow for fast handling and a prompt decision on your manuscript. For more details of this service, and to transfer your manuscript to another EMBO title please click on Link Not Available

Please do not share this URL as it will give anyone who clicks it access to your account.

Referee #1 (Comments on Novelty/Model System for Author):

1. Lack of appropriate controls: In the in vivo models induced by surgical procedures, appropriate negative control groups were not included or presented in parallel. The same issue is observed in several in vitro experiments. All relevant control data should be provided throughout the figures.

Referee #1 (Remarks for Author):

Zhu et al. present a study proposing a novel role of IRF7 in cardiac fibrosis. In their manuscript entitled "IRF7 links HK1-dependent histone lactylation to fibroblast activation and cardiac fibrosis", the authors demonstrate that loss of IRF7 exacerbates fibrotic remodeling following TAC or LAD ligation, whereas IRF7 overexpression attenuates fibrosis under similar pathological stimuli. Genomic analyses suggest that IRF7 directly regulates HK1 transcription, thereby influencing intracellular lactate levels. Loss of IRF7 subsequently promotes histone lactylation, which in turn enhances fibrotic gene expression. While the study addresses an interesting mechanism linking metabolism and epigenetic regulation in cardiac fibrosis, several issues need to be carefully addressed before the manuscript can be further considered:

We thank the reviewer for his/her insightful comments and constructive critiques. Please see below our point-by-point response to the reviewer's omments.

1. Lack of appropriate controls: In the in vivo models induced by surgical procedures, appropriate negative control groups were not included or presented in parallel. The same issue is observed in several in vitro experiments. All relevant control data should be provided throughout the figures.

Response: We appreciate the reviewer's concern and we thank the reviewer for his/her suggestion. We have since performed additional control experiments. The data are summarized in revised Figure 1A, Figure 1B, Figure 1C, Figure 1D, Figure 1E, Figure 2F, Figure 2G, Figure 2P, Figure 2Q, Figure 5B, Figure 5C, Figure 5D, Figure 5E, Figure 5F, Figure 5G, Figure 5I, Figure 5J, Figure 5L, Figure 5M, Figure 5N, Figure 5O, Figure 5P, Figure 5Q, Figure 5S, and Figure 5T. We hope that the reviewer will find the additions of new controls satisfactory.

2. Mechanistic interpretation of histone lactylation: The manuscript should discuss how histone H3 lysine lactylation modulates transcriptional activity. As lysine acetylation generally promotes transcription, the authors need to clarify whether lactylation exerts similar or distinct effects.

Response: We thank the reviewer for raising this intriguing yet critical point. Indeed, histone acetylation has traditionally been believed to be the predominant form of modification that defines active transcription before the discovery of lysine lactylation. Therefore, a key

question left unaddressed by our study is whether and, if so, how a dynamic interplay between lysine acetylation and lysine lactylation coordinately regulates transcription in cardiac fibroblasts and beyond. The reviewer would probably agree that addressing this question would entail years of work that go far beyond the scope of the present study. On the other hand, we freely admit that in this regard the present study merely puts the notion that lysine lactylation functions as key layer of transcriptional activation in the context of cardiac fibrosis without breaking further ground. We have explicitly discussed this limitation in the expanded Discussion section.

3. Ethical approval disclosure: The manuscript must include the approval numbers for both IACUC (animal study) and IRB (human sample) protocols.

Response: We thank the reviewer for his/her suggestion. The Methods section has been updated to include approval numbers for both animal and human studies.

4. Inconsistency in experimental schedules: The experimental timelines presented in Figures 2A and 2K, as well as Figures 5A and 5K, differ from each other without clear explanation. The rationale for these discrepancies should be clarified.

Response: We appreciate the reviewer's concern. The apparent discrepancy may have resulted from the different protocols used to induce Cre recombination in the two (*Col1a2*-Cre^{ERT2} and *Postn*-Cre^{ERT2}) mouse strains. As a matter of fact, the duration of the TAC procedure for both models are the same (6 weeks or 42 days) in both models. Both Cre strains are widely used in the field and we simply followed previously established protocols. We have provided more detailed descriptions and relevant references in the Methods section.

5. Need for ChIP validation: The data shown in Figure 4N should be replaced or supplemented with ChIP results to confirm the direct binding of IRF7 to the HK1 promoter.

Response: We thank the reviewer for his/her suggestion. As requested, we have performed ChIP assays to evaluate direct binding of IRF7 to the *Hk1* promoter. As shown in the new Figure 4O, IRF7 can directly bind to the *Hk1* promoter in quiescent cardiac fibroblasts, which can be inhibited by TGF-β treatment.

6. Inconsistent experimental comparisons: The rationale for certain comparisons, such as that shown in Figure 1A, is unclear. Specifically, why was TGF-β treatment applied only to the siIRF7#2 condition? Similar inconsistencies are noted in other figures and should be justified.

Response: We appreciate the reviewer's concern. The confusion referred to by the reviewer in Figure 1A appears to be a human error in which some of the the groups ere mislabeled and has been corrected. We apologize for the confusion. We have carefully examined all the figures to ensure that they are correctly and intuitively labeled.

7. Missing loading control: In Figure 6C, total histone H3 should be included as

a loading control for the histone lactylation analysis.

Response: We thank the reviewer for his/her suggestion. Blots for total histone H3 and histone H4 have been included in the revised Figure 6C.

Referee #2 (Comments on Novelty/Model System for Author):

This study used different cardiac heart failure models including TAC and myocardial infarction with target genes knockout and overexpression, demonstrated the regulatory effects of IRF7 and its downstream HK1 in cardiac fibroblasts and fibrosis. The amount of data and the results are impressive. Moreover, an inhibitor of HK1's effect was tested and also IRF7 and HK1's expression were examined in clinical samples, highlights the translational potential of the results.

Referee #2 (Remarks for Author):

This study examined the role of IRF7 and its downstream target HK1 in cardiac fibrosis. Two cardiac heart failure models including TAC and myocardial infarction with target genes knockout and overexpression were used. The amount of data and the results are impressive. Moreover, an inhibitor of HK1's effect was tested and also IRF7 and HK1's expression were examined in clinical samples, highlights the translational potential of the results.
We thank the reviewer for his/her insightful comments and constructive critiques and for his/her enthusiastic support. Please see below our point-by-point response to the reviewer's comments.

Despite these, there are several concerns need to be addressed:
1. The major concern of the study is that the link between IRF7 and HK1 is weak. Although there are data showing that IFR7 knockout or overexpression leads to changes of HK1 in their models. However, how IFR7 regulates HK1 in cardiac fibroblasts is still not clear. Whether that is through promoting HK1 transcription (and by how?) or other mechanism, is unclear. Also, whether manipulation of HK1 will affect IFR7's effects on fibrosis is also not clear.
Response: We thank the reviewer for his/her suggestions.
(1) We have ChIP assay data to show that IRF7 can directly bind to the *Hk1* promoter in quiescent cardiac fibroblasts, which can be inhibited by TGF-β treatment (Figure 4O). In addition, reporter assay demonstrates that IRF7 can directly repress the *Hk1* promoter activity, which relies on a single IRF binding motif located around -1800 relative to the transcription start site (Figure 4N).
(2) To demonstrate the functional interplay between IRF7 and HK1 (whether HK1 manipulation would affect IRF7's effects on fibrosis), we performed rescue/supplementation experiments. As shown in supplementary Figure 9, HK1 knockdown blunts the amplification of TGF-β induced fibroblast activation by IRF7 knockdown. As shown in supplementary Figure 10, HK1 over-expression relieves suppression of TGF-β induced fibroblast activation by IRF7 over-expression. These data suggest that IRF7 may regulate fibroblast activation through repressing HK1 transcription.

2. The knockout mice were generated using either Col1a2 or postn cre mice, a

discussion is needed to point out this is not deletion of IRF7 or HK1 in all fibroblasts but activated fibroblasts (vs. TCF21-Cre)

Reponse: We appreciate the reviewer's concern. The Col1a2-Cre-ERT2 strain has been widely used as a driver for fibroblast-specific gene targeting including in the heart (PMID: 24899689, 25317562, 27867037, 29160304, 29601781, 33500351, 34132780, 34324438, 36438502, 37227779, 37437652, 38503742, 38682176). That being said, we recognize that there are now other Cre drivers (e.g., Tcf21-MCM) that offer increased specificity and efficiency for gene targeting in quiescent cardiac fibroblasts. We have added a few sentences in the Discussion section to explicitly spell out the limitation.

3. It looks like the tamoxifen system is used differently in different model, is there any specific reason for this? Fig.1A vs Fig.K.

Reponse: We appreciate the reviewer's concern. Both Cre strains are widely used in the field and we simply followed previously established protocols. We have provided more detailed descriptions and relevant references in the Methods section.

4. For several experiments presented, such as Fig.1F-H, Fig2 E-K, control groups are missing. E.g., does IFR7 knockdown or overexpression affect fibrosis gene expression?

Response: We appreciate the reviewer's concern and we thank the reviewer for his/her suggestion. We have since performed additional control experiments. The data are summarized in revised Figure 1A, Figure 1B, Figure 1C, Figure 1D, Figure 1E, Figure 2F, Figure 2G, Figure 2P, Figure 2Q, Figure 5B, Figure 5C, Figure 5D, Figure 5E, Figure 5F, Figure 5G, Figure 5I, Figure 5J, Figure 5L, Figure 5M, Figure 5N, Figure 5O, Figure 5P, Figure 5Q, Figure 5S, and Figure 5T. These data collectively suggest that IRF7 manipulation by itself has a minimal impact on cardiac fibrosis.

5. Please provide representative images of echocardiography.

Reponse: We thank the reviewer for his/her suggestion. Representative echo images are now presented in the new Appendix Figure S12.

6. For AAV overexpression, please provide protein expression data to see how much the target was increased.

Reponse: We thank the reviewer for his/her suggestion. We have performed Western blotting to show the over-expression efficiency of AAV-IRF7 and to differentiate endogenous/exogenous IRF7 in heart tissues. The new data are now summarized in the new Appendix Figure S6.

7. For the MI model, please provide data for the changes of infraction and also presentative images for the histological staining of the hearts.

Reponse: We thank the reviewer for his/her suggestion. We have performed TTC staining to evaluate infarct size of the heart. The new data, which indicate that fibroblast/myofibroblast IRF7 deletion did not influence infarct size, are are now presented as the new Appendix Figure S4F and Figure S5F.

Referee #3 (Comments on Novelty/Model System for Author):

Although genomic analyses are quantitatively performed, protein level analyses are not quantitatively analysed. Need substantial analyses.

Referee #3 (Remarks for Author):

This study is intersting.

We thank the reviewer for his/her insightful comments and constructive critiques. Please see below our point-by-point response to the reviewer's comments.

One serious concenrn is that quantitative statistical analyses at the protein level (i.e., immunohistochemistry, picrosirus red staining and western blotting bands) are generally lacking. Also, immunoblot bands should be published not only as cropped images but also as images of the whole membranes.

Response: We thank the reviewer for his/her suggestion.

(1) We have performed and provided quantification for all histological stainings in the revised manuscript.

(2) Per journal policy, full-length uncropped blots will be published along with all the other raw data should our manuscript be accepted for publication.

11th Feb 2026

Dear Dr. Kong,

Thank you for submitting your revised manuscript to EMBO Molecular Medicine. The manuscript has been reviewed by the three original referees. As you will see below, while referees #2 and #3 are overall satisfied with the revisions, referee #1 raises a major concern regarding the heart failure model.

We would therefore like to invite you to revise the manuscript further to address this remaining important issue, as well as the other referees' concerns.

As EMBO Press usually only allows one round of revisions, please be aware that this will be your last opportunity to address these issues. The revised manuscript will be reviewed again, and we cannot guarantee a positive outcome at this stage.

Should you find that the requested revisions are not feasible within the constraints outlined here and prefer, therefore, to submit your paper elsewhere, we would welcome a message to this effect.

I look forward to seeing a revised form of your manuscript as soon as possible.

With kind regards,

Lise Roth

Lise Roth, Ph.D
Senior Editor
EMBO Molecular Medicine

When preparing your revised manuscript, please refer to our guidelines: https://link.springer.com/journal/44321/submission-guidelines#cms-Revised-submissions. We perform an initial quality control of all revised manuscripts before re-review; failure to include requested items will delay the evaluation of your revision.

We require:

1) A .docx formatted version of the manuscript text (including legends for main figures, EV figures and tables). Please make sure that the changes are highlighted to be clearly visible.

2) Individual production quality figure files as .eps, .tif, .jpg (one file per figure). For guidance, download the 'Figure Guide PDF': https://media.springernature.com/original/springer-cms/rest/v1/content/27825798/data/v1.

3) A .docx formatted letter INCLUDING the reviewers' reports and your detailed point-by-point responses to their comments. As part of the EMBO Press transparent editorial process, the point-by-point response is part of the Review Process File (RPF), which will be published alongside your paper.

4) A complete author checklist, which you can download from our author guidelines. Please insert information in the checklist that is also reflected in the manuscript. The completed author checklist will also be part of the RPF.

5) Please note that all corresponding authors are required to supply an ORCID ID for their name upon submission of a revised manuscript.

6) It is mandatory to include a 'Data Availability' section after the Materials and Methods. Before submitting your revision, primary datasets produced in this study need to be deposited in an appropriate public database, and the accession numbers and database listed under 'Data Availability'. Please remember to provide a reviewer password if the datasets are not yet public.

In case you have no data that requires deposition in a public database, please state so in this section. Note that the Data Availability Section is restricted to new primary data that are part of this study.

7) For data quantification: please specify the name of the statistical test used to generate error bars and P values, the number (n) of independent experiments (specify technical or biological replicates) underlying each data point and the test used to calculate p-values in each figure legend. The figure legends should contain a basic description of n, P and the test applied. Graphs must include a description of the bars and the error bars (s.d., s.e.m.).

8) At EMBO Press we ask authors to provide source data for the main manuscript figures. You will receive a separate email with instructions for providing source data with your revised manuscript, including how to upload and organize the files.

9) Our journal encourages inclusion of *data citations in the reference list* to directly cite datasets that were re-used and obtained from public databases. Data citations in the article text are distinct from normal bibliographical citations and should directly link to the database records from which the data can be accessed. In the main text, data citations are formatted as follows: "Data ref: Smith et al, 2001" or "Data ref: NCBI Sequence Read Archive PRJNA342805, 2017". In the Reference list, data citations must be labeled with "[DATASET]". A data reference must provide the database name, accession number/identifiers and a resolvable link to the landing page from which the data can be accessed at the end of the reference.

10) We replaced Supplementary Information with Expanded View (EV) Figures and Tables that are collapsible/expandable online. A maximum of 5 EV Figures can be typeset. EV Figures should be cited as 'Figure EV1, Figure EV2" etc... in the text and their respective legends should be included in the main text after the legends of regular figures.

- For the figures that you do NOT wish to display as Expanded View figures, they should be bundled together with their legends in a single PDF file called *Appendix*, which should start with a short Table of Content. Appendix figures should be referred to in the main text as: "Appendix Figure S1, Appendix Figure S2" etc.

- Additional Tables/Datasets should be labeled and referred to as Table EV1, Dataset EV1, etc. Legends have to be provided in a separate tab in case of .xls files. Alternatively, the legend can be supplied as a separate text file (README) and zipped together with the Table/Dataset file.

11) The paper explained: EMBO Molecular Medicine articles are accompanied by a summary of the articles to emphasize the major findings in the paper and their medical implications for the non-specialist reader. Please provide a draft summary of your article highlighting
- the medical issue you are addressing,
- the results obtained and
- their clinical impact.

This may be edited to ensure that readers understand the significance and context of the research. Please refer to any of our published articles for an example.

12) Author contributions: You will be asked to provide CRediT (Contributor Role Taxonomy) terms in the submission system. These replace a narrative author contribution section in the manuscript.

13) A Conflict of Interest statement should be provided in the main text.

14) Every published paper includes a 'Synopsis' to further enhance discoverability. Synopses are displayed on the journal webpage and are freely accessible to all readers. They include a short stand first (maximum of 300 characters, including space) as well as 2-5 one-sentences bullet points that summarizes the paper. Please write the bullet points to summarize the key NEW findings. They should be designed to be complementary to the abstract - i.e. not repeat the same text. We encourage inclusion of key acronyms and quantitative information (maximum of 30 words / bullet point). Please use the passive voice. Please attach these in a separate file or send them by email, we will incorporate them accordingly.

Please also suggest a striking image or visual abstract to illustrate your article as a PNG file 550 px wide x 300-600 px high.

15) Include a Reagents and Tools Table as part of the Methods section, which can be downloaded from our author guidelines.

EMBO Molecular Medicine has a "scooping protection" policy, whereby similar findings that are published by others during review or revision are not a criterion for rejection. Should you decide to submit a revised version, I do ask that you get in touch after three months if you have not completed it, to update us on the status.

***** Reviewer's comments *****

Referee #1 (Remarks for Author):

The authors have appropriately addressed many of the issues raised during the previous round of review by adding additional experiments and supplementary data, thereby improving the overall quality and completeness of the manuscript. While the reviewer generally finds the revised version acceptable, the following points require a clear response and/or additional clarification.

First, as described in Figs. 2, 3, 5, and 7, the authors maintained TAC for at least 6 weeks to induce cardiac fibrosis and non-ischemic heart failure, and they present HW/BW and EF data suggesting successful induction of fibrosis and cardiac dysfunction. In practice, however, after ~6 weeks of TAC (although the extent can vary depending on the degree of constriction), the left ventricular chamber typically becomes dilated and the overall heart size increases. In contrast, the representative gross heart/tissue images provided by the authors do not show apparent LV chamber dilation or an increase in overall heart size; rather, they appear similar to sham-operated controls. The authors should comment on this discrepancy and clarify whether the presented images are representative of the TAC cohort. If available, the authors are encouraged to provide additional representative images and/or quantitative morphometric indices (e.g., LV chamber dimension/area, wall thickness, heart cross-sectional area) to support the phenotypic interpretation. Please check the reference (https://www.frontiersin.org/journals/cardiovascular-medicine/articles/10.3389/fcvm.2023.1110032/full)

Second, histone lysine lactylation is a relatively recent post-translational modification. Therefore, rather than limiting the discussion to a conceptual level, it would strengthen the manuscript to provide at least an overview of how canonical histone marks are altered in parallel. The reviewer requests that the authors include representative data (even if limited in scope) showing whether major histone acetylation and methylation marks are changed in the same experimental setting (e.g., selected acetylation/methylation marks by immunoblotting or other appropriate assays), and incorporate these findings into the discussion.

Referee #2 (Comments on Novelty/Model System for Author):

Several cardiac injury models are used that improve the confidence of the findings.

Referee #2 (Remarks for Author):

Thanks for addressing the comments with new data.
One minor point: If it is possible, it would be great to incorporate echo images into main figures, rather than group them together as a supplement.

Referee #3 (Comments on Novelty/Model System for Author):

This revised manuscript has been well improved with solid results. I have no more concerns.

Referee #3 (Remarks for Author):

The authors sufficiently responded to my concerns.

Referee #1 (Remarks for Author):

The authors have appropriately addressed many of the issues raised during the previous round of review by adding additional experiments and supplementary data, thereby improving the overall quality and completeness of the manuscript. While the reviewer generally finds the revised version acceptable, the following points require a clear response and/or additional clarification.

We thank the reviewer for his/her continued interest in our manuscript and for his/her insightful comments and suggestions to improve our manuscript. Please see below our point-by-point response to the reviewer's comments and concerns.

First, as described in Figs. 2, 3, 5, and 7, the authors maintained TAC for at least 6 weeks to induce cardiac fibrosis and non-ischemic heart failure, and they present HW/BW and EF data suggesting successful induction of fibrosis and cardiac dysfunction. In practice, however, after ~6 weeks of TAC (although the extent can vary depending on the degree of constriction), the left ventricular chamber typically becomes dilated and the overall heart size increases. In contrast, the representative gross heart/tissue images provided by the authors do not show apparent LV chamber dilation or an increase in overall heart size; rather, they appear similar to sham-operated controls. The authors should comment on this discrepancy and clarify whether the presented images are representative of the TAC cohort. If available, the authors are encouraged to provide additional representative images and/or quantitative morphometric indices (e.g., LV chamber dimension/area, wall thickness, heart cross-sectional area) to support the phenotypic interpretation. Please check the reference (https://www.frontiersin.org/journals/cardiovascular-medicine/articles/10.3389/fcvm.2023.1110032/full)

We appreciate the reviewer's concern and we apologize for the confusion. The upper panels of these figures referred to by the reviewer were resized for aesthetic purposes and not intended for direct comparison of hypertrophy (note that no scale bar was provided for the upper panels). We have since adjusted the upper panels of Figure 2G, Figure 2R, Figure 3J, Figure 5G, Figure 5R, and Figure 7L to show the actual size of the heart with scale bars added for direct comparison of hypertrophy. In addition, we have included additional echo measurement of hypertrophy (LV mass), which are presented as the new Figure 2F, Figure 2Q, Figure 3E, Figure 5F, and Figure 5Q. We hope these changes will be satisfactory to address the reviewer's concern.

Second, histone lysine lactylation is a relatively recent post-translational modification. Therefore, rather than limiting the discussion to a conceptual level, it would strengthen the manuscript to provide at least an overview of how canonical histone marks are altered in parallel. The reviewer requests that the authors include representative data (even if limited in scope) showing whether major histone acetylation and methylation marks are changed in the same experimental setting (e.g., selected acetylation/methylation marks by immunoblotting or other appropriate assays), and incorporate these findings into the discussion.

We thank the reviewer for his/her suggestion. As requested, we have performed additional Western blotting experiments to examine the changes in H3K9 acetylation, H3K27 acetylation, and H3K4 trimethylation under the same setting. As shown in the updated Figure 6C, neither H3K9/H3K27 acetylation nor H3K4 trimethylation underwent the same alteration as H3K9 lactylation. We have also discussed these data further in the expanded Discussion section.

Referee #2 (Comments on Novelty/Model System for Author):

Several cardiac injury models are used that improve the confidence of the findings.

Referee #2 (Remarks for Author):

Thanks for addressing the comments with new data.
One minor point: If it is possible, it would be great to incorporate echo images into main figures, rather than group them together as a supplement.
We thank the reviewer for his/her time and effort invested in evaluating our manuscript. We are glad that the reviewer is satisfied with our revised manuscript. Because the figures are already crowded as they are we elect to leave the representative echo images in the Appendix.

Referee #3 (Comments on Novelty/Model System for Author):

This revised manuscript has been well improved with solid results. I have no more concerns.

Referee #3 (Remarks for Author):

The authors sufficiently responded to my concerns.

We thank the reviewer for his/her time and effort invested in evaluating our manuscript. We are glad that the reviewer is satisfied with our revised manuscript.

25th Mar 2026

Dear Dr. Kong,

Thank you for submitting your revised study and for providing the additional source data. Your manuscript has been reviewed by referee #1, and we will be able to accept your manuscript once the following minor editorial matters are addressed:

1/ Manuscript text:
- Please remove the red font text and only keep in track changes mode any new modification.
- Please provide an institutional email for Junli Guo.
- Please carefully check your text for language and grammar, and make sure to use a scientific language. If applicable, use of an AI-based editing tool must be mentioned.
- Methods:
o Please provide antibody dilutions.
o Human samples: Please fill the checklist in the fields 'ethics approval', and 'Declaration of Helsinki'. In the methods, statement on the declaration of Helsinki should be mentioned in the paragraph "Human heart specimens".
o Statistics: provide a statement on blinding, randomization, sample size, and inclusion/exclusion criteria, and fill the checklist accordingly.
o Cell lines: if cell lines were used (as indicated in the checklist), origin and statements on authentication and mycoplasma contamination should be provided.
- Data Availability: please provide specific URLs for GSE317630, GSE317712 datasets.
- Acknowledgements: Fundings 82400729 and 82370633 are missing in our submission system, please add.
- Please rename "Conflict of interest" to "Disclosure and competing interests statement", and remove duplicate from the title page.

2/ Figures:
- We encourage you to make some of your Appendix figures into Expanded View (EV) Figures and Tables that are collapsible/expandable online. EV Figures should be cited as 'Figure EV1, Figure EV2" etc... in the text and their respective legends should be included in the main text after the legends of regular figures. For the figures that you do NOT wish to display as Expanded View figures, they should be bundled together with their legends in a single PDF file called *Appendix*, which should start with a short Table of Content. Appendix figures should be referred to in the main text as: "Appendix Figure S1, Appendix Figure S2" etc.
- Additional Tables/Datasets should be labeled and referred to as Table EV1, Dataset EV1, etc. Legends have to be provided in a separate tab in case of .xls files. Alternatively, the legend can be supplied as a separate text file (README) and zipped together with the Table/Dataset file.
- Please note that figure re-use is allowed, but should be mentioned in the figure legends.
- Please carefully check the composition of figure 1B and 1G. If changes are introduced, please provide an explanation.
- Please carefully check the zoom in squares provided in Figure 2G, top pictures (as well as in other figures that have been modified from the previous version), as the selected areas do not seem to reflect the zoom-in versions (bottom pictures).
- Please make sure all figure panels are referenced in the text. Currently, callouts are missing for Appendix Table S1 and Figure 3N. Appendix figure citations should be "Appendix Figure S1" etc.; please correct the callouts for "Suppl, Table I" and "Suppl Table II".
- In figure 1, please correct the panel label from K to J.
- If you use an image data base for scientific iconography (e.g., BioRender), it should be indicated in the manuscript.
- Please address the queries from our data editors in the figure legends:
1. Please indicate the statistical test used for data analysis in the legends of figures 6E, G, H; 8C, D, F.
2. Please note that the box plots need to be defined in terms of minima, maxima, centre, bounds of box and whiskers, and percentile in the legend of figure 6G
3. Please note that information related to n is missing in the legends of figures 6G, 8E
4. Please note that the error bars are not defined in the legends of figures 2B, C, D, E, F, H, G, I, J, K, M, N, O, P, Q, R, S, T, U, V; 3B, C, D, E, F, G, H, I, J, K, L, M, N; 5B, C, D, E, F, G, H, I, J, K, M, N, O, P, Q, R, S, T, U, V; 7H-Q; 8A, B, E
5. Please note that the scale bar needs to be defined for figures 1B-D, G-I

3/ Thank you for providing source data. Please upload them as one file per figure and make sure they are provided for each panel. For the files that are too big (such as the ones you kindly provided to use for figure check), please upload them on a repository such as Biostudies, and provide the link in the Data Availability Section. Please provide original Western blot images for Figure 4.

4/ I have introduced minor edits in your Paper Explained, please let me know if you agree or amend as you see fit:
Problem
Diffuse myocardial fibrosis impairs heart function, leading to heart failure (HF). The trans-differentiation of quiescent cardiac

fibroblasts into myofibroblasts is a key process in cardiac fibrosis.
Results
We present a new mechanism through which the transcription factor IRF7 connects HK1-mediated histone lactylation to fibroblasts - myofibroblasts transition and cardiac fibrosis. Genetic and/or pharmacological manipulation of the IRF7-HK1 axis in mice improves cardiac fibrosis and prevents heart failure. Notably, a correlation between IRF7, HK1 and myofibroblast activation was identified in heart tissue samples taken from patients with heart failure (HF).
Impact
These data offer new insights and translational potential for HF interventions.

5/ Please provide a visual abstract and a synopsis text:
Synopses are displayed on the journal webpage and are freely accessible to all readers. They include a short stand first (maximum of 300 characters, including space) as well as 2-5 one-sentences bullet points that summarizes the paper (maximum of 30 words / bullet point). Please write the bullet points to summarize the key NEW findings. They should be designed to be complementary to the abstract - i.e. not repeat the same text.

Please also suggest a striking image or visual abstract to illustrate your article as a tiff/jpeg/PNG file 550 px wide x 300-600 px high.

6/ As part of the EMBO Publications transparent editorial process initiative (see our Editorial at http://embomolmed.embopress.org/content/2/9/329), EMBO Molecular Medicine will publish online a Review Process File (RPF) to accompany accepted manuscripts.
This file will be published in conjunction with your paper and will include the anonymous referee reports, your point-by-point response and all pertinent correspondence relating to the manuscript. Let us know whether you agree with the publication of the RPF and as here.
Please note that the Authors checklist will be published at the end of the RPF.

I look forward to receiving your revised manuscript.

Yours sincerely,

Lise Roth

Lise Roth, PhD
Senior Editor
EMBO Molecular Medicine

***** Reviewer's comments *****

Referee #1 (Remarks for Author):

The current version of the manuscript and the main figures appear, at least apparently, to be well organized.

1/ Manuscript text:
- Please remove the red font text and only keep in track changes mode any new modification.
Done.

- Please provide an institutional email for Junli Guo.
Provided.

- Please carefully check your text for language and grammar, and make sure to use a scientific language. If applicable, use of an AI-based editing tool must be mentioned.
Done.

- Methods:
o Please provide antibody dilutions.
Provided in Appendix Table S2.

o Human samples: Please fill the checklist in the fields 'ethics approval', and 'Declaration of Helsinki'. In the methods, statement on the declaration of Helsinki should be mentioned in the paragraph "Human heart specimens".
Updated.

o Statistics: provide a statement on blinding, randomization, sample size, and inclusion/exclusion criteria, and fill the checklist accordingly.
Done.

o Cell lines: if cell lines were used (as indicated in the checklist), origin and statements on authentication and mycoplasma contamination should be provided.
Not applicable.

- Data Availability: please provide specific URLs for GSE317630, GSE317712 datasets.
Done.

- Acknowledgements: Fundings 82400729 and 82370633 are missing in our submission system, please add.
Added.

- Please rename "Conflict of interest" to "Disclosure and competing interests statement", and remove duplicate from the title page.
Renamed and removed.

2/ Figures:
- We encourage you to make some of your Appendix figures into Expanded View (EV) Figures and Tables that are collapsible/expandable online. EV Figures should be cited as 'Figure EV1, Figure EV2" etc... in the text and their respective legends should be

included in the main text after the legends of regular figures. For the figures that you do NOT wish to display as Expanded View figures, they should be bundled together with their legends in a single PDF file called *Appendix*, which should start with a short Table of Content. Appendix figures should be referred to in the main text as: "Appendix Figure S1, Appendix Figure S2" etc.
Renamed.

- Additional Tables/Datasets should be labeled and referred to as Table EV1, Dataset EV1, etc. Legends have to be provided in a separate tab in case of .xls files. Alternatively, the legend can be supplied as a separate text file (README) and zipped together with the Table/Dataset file.
Not applicable.

- Please note that figure re-use is allowed, but should be mentioned in the figure legends.
Not applicable.

- Please carefully check the composition of figure 1B and 1G. If changes are introduced, please provide an explanation.
Checked.

- Please carefully check the zoom in squares provided in Figure 2G, top pictures (as well as in other figures that have been modified from the previous version), as the selected areas do not seem to reflect the zoom-in versions (bottom pictures).
Checked and corrected.

- Please make sure all figure panels are referenced in the text. Currently, callouts are missing for Appendix Table S1 and Figure 3N.
Corrected.

Appendix figure citations should be "Appendix Figure S1" etc.; please correct the callouts for "Suppl, Table I" and "Suppl Table II".
Corrected.

- In figure 1, please correct the panel label from K to J.
Corrected.

- If you use an image data base for scientific iconography (e.g., BioRender), it should be indicated in the manuscript.
Not applicable.

- Please address the queries from our data editors in the figure legends:
1. Please indicate the statistical test used for data analysis in the legends of figures 6E, G, H; 8C, D, F.

Added.

2. Please note that the box plots need to be defined in terms of minima, maxima, centre, bounds of box and whiskers, and percentile in the legend of figure 6G
Defined.

3. Please note that information related to n is missing in the legends of figures 6G, 8E
Added.

4. Please note that the error bars are not defined in the legends of figures 2B, C, D, E, F, H, G, I, J, K, M, N, O, P, Q, R, S, T, U, V; 3B, C, D, E, F, G, H, I, J, K, L, M, N; 5B, C, D, E, F, G, H, I, J, K, M, N, O, P, Q, R, S, T, U, V; 7H-Q; 8A, B, E
Defined in the legends.

5. Please note that the scale bar needs to be defined for figures 1B-D, G-I
Defined in the legend.

3/ Thank you for providing source data. Please upload them as one file per figure and make sure they are provided for each panel. For the files that are too big (such as the ones you kindly provided to use for figure check), please upload them on a repository such as Biostudies, and provide the link in the Data Availability Section. Please provide original Western blot images for Figure 4.
Complied.

4/ I have introduced minor edits in your Paper Explained, please let me know if you agree or amend as you see fit:
Problem
Diffuse myocardial fibrosis impairs heart function, leading to heart failure (HF). The trans-differentiation of quiescent cardiac fibroblasts into myofibroblasts is a key process in cardiac fibrosis.
Results
We present a new mechanism through which the transcription factor IRF7 connects HK1-mediated histone lactylation to fibroblasts-myofibroblasts transition and cardiac fibrosis. Genetic and/or pharmacological manipulation of the IRF7-HK1 axis in mice mitigates cardiac fibrosis and prevents heart failure. Notably, a correlation between IRF7, HK1 and myofibroblast activation was identified in heart tissue samples taken from patients with heart failure (HF).
Impact
These data offer new insights and translational potential for HF intervention.
Adopted.

5/ Please provide a visual abstract and a synopsis text:
Synopses are displayed on the journal webpage and are freely accessible to all readers. They include a short stand first (maximum of 300 characters, including space) as well

as 2-5 one-sentences bullet points that summarizes the paper (maximum of 30 words / bullet point). Please write the bullet points to summarize the key NEW findings. They should be designed to be complementary to the abstract - i.e. not repeat the same text.
Please also suggest a striking image or visual abstract to illustrate your article as a tiff/jpeg/PNG file 550 px wide x 300-600 px high.
Provided.

6/ As part of the EMBO Publications transparent editorial process initiative (see our Editorial at http://embomolmed.embopress.org/content/2/9/329), EMBO Molecular Medicine will publish online a Review Process File (RPF) to accompany accepted manuscripts.
This file will be published in conjunction with your paper and will include the anonymous referee reports, your point-by-point response and all pertinent correspondence relating to the manuscript. Let us know whether you agree with the publication of the RPF and as here.
Please note that the Authors checklist will be published at the end of the RPF.
Consented.

9th Apr 2026

Dear Dr. Kong,

Thank you for submitting your revised files. A few editorial matters remain to be resolved before I can accept your manuscript:

1. I introduced minor modifications in your abstract, please let me know if you agree:
"In response to various stimuli, quiescent resident cardiac fibroblasts undergo metabolic, morphological, and functional alterations, transitioning into myofibroblasts that mediate cardiac fibrosis. In the present study, we investigated the role of interferon regulatory factor 7 (IRF7) in fibroblast activation and cardiac fibrosis. Knockdown of IRF7 in quiescent cardiac fibroblasts potentiated myofibroblast transition, whereas over-expression of IRF7 suppressed it. Furthermore, targeted deletion of IRF7 in fibroblasts and myofibroblasts exacerbated cardiac fibrosis and impaired heart function in animal models of heart failure. Integrated transcriptomic analysis revealed hexokinase 1 (HK1) as an IRF7 downstream target. Consistently, genetic deletion or pharmacological inhibition of HK1 protected mice from adverse cardiac remodeling. Mechanistically, HK1 contributed to the cellular lactate pool, promoting histone H3K9 lactylation and enabling the transcription of pro-fibrogenic molecules. Finally, the relevance of the IRF7-HK1 axis was verified in human heart specimens. In conclusion, our data unveil an IRF7-HK1 axis that contributes to the metaboloepigenetic reprogramming of fibroblast activation and cardiac fibrosis. Targeting this axis may provide new therapeutic solutions for heart failure intervention."

As mentioned before, please carefully check your text, and make sure to use a scientific language (avoid langague such as "chief effector", which should be changed for major effector, or "hunt", which should be changed for search, etc).

2. Methods:
a. Statistics: please define the inclusion/exclusion criteria (currently referred to as "clear technical errors").
b. Checklist: you filled in the section on cell materials/cell lines, but mentioned this was not applicable. Please correct as needed.
c. Datasets must be publicly accessible before acceptance of the manuscript.

3. Synopsis:
a. The visual abstract and panel 8G are identical, please remove Figure 8G to keep this visual abstract.
b. Synopsis text: I introduced minor edits, please let me know if you agree:

"The activation of quiescent fibroblasts contributes to adverse ventricular remodeling following myocardial injury, which can lead to heart failure. The transcription factor IRF7 links HK1-dependent histone lactylation to fibroblast activation and cardiac fibrosis.
- IRF7 deletion promotes fibroblast activation and aggravates cardiac fibrosis.
- IRF7 over-expression dampens fibroblast activation and attenuates cardiac fibrosis
- IRF7 binds to the HK1 promoter and represses HK1 transcription.
- Genetic deletion or pharmaceutical inhibition of HK1 mitigates cardiac fibrosis.
- HK1-dependent H3K9 lactylation facilitates recruitment of SMAD3, c-JUN, and NFATC2 to orchestrate pro-fibrogenic transcription.

4. Figures:
a. Scale bars are missing in Figure 1 and Figure 7 (and not defined for Figure 7), please correct.
b. Figure 2 contains 2 panels P, no panel R, please correct.

5. Source Data:
a. Figure 5: there are no data provided for 5F, two files provided for 5I.
b. Please deposit the raw imaging data provided during figure check on a repository such as Biostudies, and provide the access link in the Data Availability section.

I look forward to receiving your revised manuscript.

Yours sincerely,

Lise Roth

Lise Roth, PhD
Senior Editor
EMBO Molecular Medicine

1. I introduced minor modifications in your abstract, please let me know if you agree:
"In response to various stimuli, quiescent resident cardiac fibroblasts undergo metabolic, morphological, and functional alterations, transitioning into myofibroblasts that mediate cardiac fibrosis. In the present study, we investigated the role of interferon regulatory factor 7 (IRF7) in fibroblast activation and cardiac fibrosis. Knockdown of IRF7 in quiescent cardiac fibroblasts potentiated myofibroblast transition, whereas over-expression of IRF7 suppressed it. Furthermore, targeted deletion of IRF7 in fibroblasts and myofibroblasts exacerbated cardiac fibrosis and impaired heart function in animal models of heart failure. Integrated transcriptomic analysis revealed hexokinase 1 (HK1) as an IRF7 downstream target. Consistently, genetic deletion or pharmacological inhibition of HK1 protected mice from adverse cardiac remodeling. Mechanistically, HK1 contributed to the cellular lactate pool, promoting histone H3K9 lactylation and enabling the transcription of pro-fibrogenic molecules. Finally, the relevance of the IRF7-HK1 axis was verified in human heart specimens. In conclusion, our data unveil an IRF7-HK1 axis that contributes to the metaboloepigenetic reprogramming of fibroblast activation and cardiac fibrosis. Targeting this axis may provide new therapeutic solutions for heart failure intervention."
As mentioned before, please carefully check your text, and make sure to use a scientific language (avoid langague such as "chief effector", which should be changed for major effector, or "hunt", which should be changed for search, etc).
Changes accepted.

2. Methods:
a. Statistics: please define the inclusion/exclusion criteria (currently referred to as "clear technical errors").
Criteria more clearly Defined.

b. Checklist: you filled in the section on cell materials/cell lines, but mentioned this was not applicable. Please correct as needed.
Corrected.

c. Datasets must be publicly accessible before acceptance of the manuscript.
Freely accessible as of April 9, 2026.

3. Synopsis:
a. The visual abstract and panel 8G are identical, please remove Figure 8G to keep this visual abstract.
Figure 8G removed.
b. Synopsis text: I introduced minor edits, please let me know if you agree:

"The activation of quiescent fibroblasts contributes to adverse ventricular remodeling following myocardial injury, which can lead to heart failure. The transcription factor IRF7 links HK1-dependent histone lactylation to fibroblast activation and cardiac fibrosis.
- IRF7 deletion promotes fibroblast activation and aggravates cardiac fibrosis.
- IRF7 over-expression dampens fibroblast activation and attenuates cardiac fibrosis

- IRF7 binds to the HK1 promoter and represses HK1 transcription.
- Genetic deletion or pharmaceutical inhibition of HK1 mitigates cardiac fibrosis.
- HK1-dependent H3K9 lactylation facilitates recruitment of SMAD3, c-JUN, and NFATC2 to orchestrate pro-fibrogenic transcription.

Changes accepted.

4. Figures:

a. Scale bars are missing in Figure 1 and Figure 7 (and not defined for Figure 7), please correct.

Corrected.

b. Figure 2 contains 2 panels P, no panel R, please correct.

Corrected.

5. Source Data:

a. Figure 5: there are no data provided for 5F, two files provided for 5I.

Corrected.

b. Please deposit the raw imaging data provided during figure check on a repository such as Biostudies, and provide the access link in the Data Availability section.

Deposited.

27th Apr 2026

Dear Dr. Kong,

We are pleased to inform you that your manuscript is accepted for publication and is now being sent to our publisher to be included in the next available issue of EMBO Molecular Medicine.

Your manuscript will be processed for publication by EMBO Press. It will be copy edited and you will receive page proofs prior to publication. Please note that you will be contacted by Springer Nature Author Services to complete licensing and payment information.

Please note that I had to introduce numerous edits to your Introduction and Discussion sections. I would therefore ask you to carefully review the manuscript during the proofreading stage to ensure that no errors were inadvertently introduced.

You may qualify for financial assistance for your publication charges - either via a Springer Nature fully open access agreement or an EMBO initiative. Check your eligibility: https://link.springer.com/journal/44321/how-to-publish-with-us

Should you be planning a Press Release on your article, please get in contact with embo_production@springernature.com as early as possible in order to coordinate publication and release dates.

If you have any questions, please do not hesitate to contact the Editorial Office. Thank you for your contribution to EMBO Molecular Medicine.

Yours sincerely,

Lise Roth, Ph.D
Senior Editor
EMBO Molecular Medicine

>>> Please note that it is EMBO Molecular Medicine policy for the transcript of the editorial process (containing referee reports and your response letter) to be published as an online supplement to each paper. If you do NOT want this, you will need to inform the Editorial Office via email immediately. More information is available here: https://link.springer.com/partners/embo-press/editorial-policies#Peer%20review

EMBO Press Author Checklist

Corresponding Author Name: Ming Kong	
Journal Submitted to: EMBO Mol Med	
Manuscript Number: EMM-2025-22683-V2	

USEFUL LINKS FOR COMPLETING THIS FORM
The EMBO Journal - Author Guidelines
EMBO Reports - Author Guidelines
Molecular Systems Biology - Author Guidelines
EMBO Molecular Medicine - Author Guidelines

Reporting Checklist for Life Science Articles (updated January
This checklist is adapted from Materials Design Analysis Reporting (MDAR) Checklist for Authors. MDAR establishes a minimum set of requirements in transparent reporting in the life sciences (see Statement of Task: 10.31222/osf.io/9sm4x). Please follow the journal's guidelines in preparing your
Please note that a copy of this checklist will be published alongside your article.

Abridged guidelines for figures
1. Data
The data shown in figures should satisfy the following conditions:
- the data were obtained and processed according to the field's best practice and are presented to reflect the results of the experiments in an accurate and unbiased manner.
- ideally, figure panels should include only measurements that are directly comparable to each other and obtained with the same assay.
- plots include clearly labeled error bars for independent experiments and sample sizes. Unless justified, error bars should not be shown for technical
- if n<5, the individual data points from each experiment should be plotted. Any statistical test employed should be justified.
- Source Data should be included to report the data underlying figures according to the guidelines set out in the authorship guidelines on Data

2. Captions
Each figure caption should contain the following information, for each panel where they are relevant:
- a specification of the experimental system investigated (eg cell line, species name).
- the assay(s) and method(s) used to carry out the reported observations and measurements.
- an explicit mention of the biological and chemical entity(ies) that are being measured.
- an explicit mention of the biological and chemical entity(ies) that are altered/varied/perturbed in a controlled manner.
- the exact sample size (n) for each experimental group/condition, given as a number, not a range;
- a description of the sample collection allowing the reader to understand whether the samples represent technical or biological replicates (including how many animals, litters, cultures, etc.).
- a statement of how many times the experiment shown was independently replicated in the laboratory.
- definitions of statistical methods and measures:
  - common tests, such as t-test (please specify whether paired vs. unpaired), simple χ2 tests, Wilcoxon and Mann-Whitney tests, can be unambiguously identified by name only, but more complex techniques should be described in the methods section;
  - are tests one-sided or two-sided?
  - are there adjustments for multiple comparisons?
  - exact statistical test results, e.g., P values = x but not P values < x;
  - definition of 'center values' as median or average;
  - definition of error bars as s.d. or s.e.m.

Please complete ALL of the questions below.
Select "Not Applicable" only when the requested information is not relevant for your study.

Materials

Newly Created Materials	Information included in the manuscript?	In which section is the information available? (Reagents and Tools Table, Materials and Methods, Figures, Data Availability Section)
New materials and reagents need to be available; do any restrictions apply?	Not Applicable	

Antibodies	Information included in the manuscript?	In which section is the information available? (Reagents and Tools Table, Materials and Methods, Figures, Data Availability Section)
For **antibodies** provide the following information:<br>- Commercial antibodies: RRID (if possible) or supplier name, catalogue number and or/clone number<br>- Non-commercial: RRID or citation	Yes	Materials and Methods

DNA and RNA sequences	Information included in the manuscript?	In which section is the information available? (Reagents and Tools Table, Materials and Methods, Figures, Data Availability Section)
**Short novel DNA or RNA including primers, probes**: provide the sequences.	Yes	Materials and Methods

Cell materials	Information included in the manuscript?	In which section is the information available? (Reagents and Tools Table, Materials and Methods, Figures, Data Availability Section)
**Cell lines:** Provide species information, strain. Provide accession number in repository **OR** supplier name, catalog number, clone number, and/**OR** RRID.	Not Applicable	
**Primary cultures:** Provide species, strain, sex of origin, genetic modification status.	Yes	Materials and Methods
Report if the cell lines were recently **authenticated** (e.g., by STR profiling) and tested for mycoplasma contamination.	Not Applicable	

Experimental animals	Information included in the manuscript?	In which section is the information available? (Reagents and Tools Table, Materials and Methods, Figures, Data Availability Section)
**Laboratory animals or Model organisms:** Provide species, strain, sex, age, genetic modification status. Provide accession number in repository **OR** supplier name, catalog number, clone number, **OR** RRID.	Yes	Materials and Methods
**Animal observed in or captured from the field:** Provide species, sex, and age where possible.	Not Applicable	
Please detail **housing and husbandry conditions**.	Yes	Materials and Methods

Plants and microbes	Information included in the manuscript?	In which section is the information available? (Reagents and Tools Table, Materials and Methods, Figures, Data Availability Section)
**Plants:** provide species and strain, ecotype and cultivar where relevant, unique accession number if available, and source (including location for collected wild specimens).	Not Applicable	
**Microbes:** provide species and strain, unique accession number if available, and source.	Not Applicable	

Human research participants	Information included in the manuscript?	In which section is the information available? (Reagents and Tools Table, Materials and Methods, Figures, Data Availability Section)
If collected and within the bounds of privacy constraints report on age, sex and gender or ethnicity for all study participants.	Yes	Materials and Methods

Core facilities	Information included in the manuscript?	In which section is the information available? (Reagents and Tools Table, Materials and Methods, Figures, Data Availability Section)
If your work benefited from core facilities, was their service mentioned in the acknowledgments section?	Not Applicable	

Design

Study protocol	Information included in the manuscript?	In which section is the information available? (Reagents and Tools Table, Materials and Methods, Figures, Data Availability Section)
If study protocol has been **pre-registered, provide DOI in the manuscript**. For clinical trials, provide the trial registration number **OR** cite DOI.	Not Applicable	
Report the **clinical trial registration number** (at ClinicalTrials.gov or equivalent), where applicable.	Not Applicable	

Laboratory protocol	Information included in the manuscript?	In which section is the information available? (Reagents and Tools Table, Materials and Methods, Figures, Data Availability Section)
Provide DOI OR other citation details if **external detailed step-by-step protocols** are available.	Yes	Materials and Methods

Experimental study design and statistics	Information included in the manuscript?	In which section is the information available? (Reagents and Tools Table, Materials and Methods, Figures, Data Availability Section)
Include a statement about **sample size** estimate even if no statistical methods were used.	Yes	Materials and Methods
Were any steps taken to minimize the effects of subjective bias when allocating animals/samples to treatment (e.g. **randomization procedure**)? If yes, have they been described?	Yes	Materials and Methods
Include a statement about **blinding** even if no blinding was done.	Yes	Materials and Methods
Describe **inclusion/exclusion criteria** if samples or animals were excluded from the analysis. Were the criteria pre-established?<br><br>If sample or data points were omitted from analysis, report if this was due to attrition or intentional exclusion and provide justification.	Yes	Materials and Methods
For every figure, are **statistical tests** justified as appropriate? Do the data meet the assumptions of the tests (e.g., normal distribution)? Describe any methods used to assess it. Is there an estimate of variation within each group of data? Is the variance similar between the groups that are being statistically compared?	Yes	Materials and Methods

Sample definition and in-laboratory replication	Information included in the manuscript?	In which section is the information available? (Reagents and Tools Table, Materials and Methods, Figures, Data Availability Section)
In the figure legends: state number of times the experiment was **replicated** in laboratory.	Yes	Figure legends
In the figure legends: define whether data describe **technical or biological replicates**.	Yes	Figure legends

Ethics

Ethics	Information included in the manuscript?	In which section is the information available? (Reagents and Tools Table, Materials and Methods, Figures, Data Availability Section)
Studies involving **human participants**: State details of **authority granting ethics approval** (IRB or equivalent committee(s), provide reference number for approval.	Yes	Materials and Methods
Studies involving **human participants**: Include a statement confirming that **informed consent** was obtained from all subjects and that the experiments conformed to the principles set out in the WMA Declaration of Helsinki and the Department of Health and Human Services Belmont Report.	Yes	Materials and Methods
Studies involving **human participants:** For publication of **patient photos**, include a statement confirming that consent to publish was obtained.	Not Applicable	
Studies involving experimental **animals**: State details of **authority granting ethics approval** (IRB or equivalent committee(s), provide reference number for approval. Include a statement of compliance with ethical regulations.	Yes	Materials and Methods
Studies involving **specimen and field samples:** State if relevant **permits** obtained, provide details of authority approving study; if none were required, explain why.	Yes	Materials and Methods

Dual Use Research of Concern (DURC)	Information included in the manuscript?	In which section is the information available? (Reagents and Tools Table, Materials and Methods, Figures, Data Availability Section)
Could your study fall under dual use research restrictions? Please check biosecurity documents and list of **select agents and toxins** (CDC): https://www.selectagents.gov/sat/list.htm	Not Applicable	
If you used a select agent, is the security level of the lab appropriate and reported in the manuscript?	Not Applicable	
If a study is subject to dual use research of concern regulations, is the name of the **authority granting approval and reference number** for the regulatory approval provided in the manuscript?	Not Applicable	

Reporting

The MDAR framework recommends adoption of discipline-specific guidelines, established and endorsed through community initiatives. Journals have their own policy about requiring specific guidelines and recommendations to complement MDAR.

Adherence to community standards	Information included in the manuscript?	In which section is the information available? (Reagents and Tools Table, Materials and Methods, Figures, Data Availability Section)
State if relevant guidelines or checklists (e.g., **ICMJE, MIBBI, ARRIVE, PRISMA**) have been followed or provided.	Not Applicable	
For **tumor marker prognostic studies,** we recommend that you follow the **REMARK** reporting guidelines (see link list at top right). See author guidelines, under 'Reporting Guidelines'. Please confirm you have followed these guidelines.	Not Applicable	
For **phase II and III randomized controlled trials**, please refer to the **CONSORT** flow diagram (see link list at top right) and submit the CONSORT checklist (see link list at top right) with your submission. See author guidelines, under 'Reporting Guidelines'. Please confirm you have submitted this list.	Not Applicable	

Data Availability

Data availability	Information included in the manuscript?	In which section is the information available? (Reagents and Tools Table, Materials and Methods, Figures, Data Availability Section)
Have **primary datasets** been deposited according to the journal's guidelines (see 'Data Deposition' section) and the respective accession numbers provided in the Data Availability Section?	Yes	Data Availability Section
Were **human clinical and genomic datasets** deposited in a public access-controlled repository in accordance to ethical obligations to the patients and to the applicable consent agreement?	Not Applicable	
Are **computational models** that are central and integral to a study available without restrictions in a machine-readable form? Were the relevant accession numbers or links provided?	Not Applicable	
If publicly available data were reused, provide the respective **data citations in the reference list**.	Yes	Figure legends
